# Autoantibodies neutralizing type I IFNs are present in ~ 4% of uninfected individuals over 70 years and account for ~ 20% of COVID-19 deaths

**DOI:** 10.1126/sciimmunol.abl4340

**Published:** 2021-08-19

**Authors:** Paul Bastard, Adrian Gervais, Tom Le Voyer, Jérémie Rosain, Quentin Philippot, Jérémy Manry, Eleftherios Michailidis, Hans-Heinrich Hoffmann, Shohei Eto, Marina Garcia-Prat, Lucy Bizien, Alba Parra-Martínez, Rui Yang, Liis Haljasmägi, Mélanie Migaud, Karita Särekannu, Julia Maslovskaja, Nicolas de Prost, Yacine Tandjaoui-Lambiotte, Charles-Edouard Luyt, Blanca Amador-Borrero, Alexandre Gaudet, Julien Poissy, Pascal Morel, Pascale Richard, Fabrice Cognasse, Jesus Troya, Sophie Trouillet-Assant, Alexandre Belot, Kahina Saker, Pierre Garçon, Jacques G. Rivière, Jean-Christophe Lagier, Stéphanie Gentile, Lindsey B. Rosen, Elana Shaw, Tomohiro Morio, Junko Tanaka, David Dalmau, Pierre-Louis Tharaux, Damien Sene, Alain Stepanian, Bruno Megarbane, Vasiliki Triantafyllia, Arnaud Fekkar, James R. Heath, José Luis Franco, Juan-Manuel Anaya, Jordi Solé-Violán, Luisa Imberti, Andrea Biondi, Paolo Bonfanti, Riccardo Castagnoli, Ottavia M. Delmonte, Yu Zhang, Andrew L. Snow, Steven M. Holland, Catherine Biggs, Marcela Moncada-Vélez, Andrés Augusto Arias, Lazaro Lorenzo, Soraya Boucherit, Boubacar Coulibaly, Dany Anglicheau, Anna M. Planas, Filomeen Haerynck, Sotirija Duvlis, Robert L. Nussbaum, Tayfun Ozcelik, Sevgi Keles, Ahmed A. Bousfiha, Jalila El Bakkouri, Carolina Ramirez-Santana, Stéphane Paul, Qiang Pan-Hammarström, Lennart Hammarström, Annabelle Dupont, Alina Kurolap, Christine N. Metz, Alessandro Aiuti, Giorgio Casari, Vito Lampasona, Fabio Ciceri, Lucila A. Barreiros, Elena Dominguez-Garrido, Mateus Vidigal, Mayana Zatz, Diederik van de Beek, Sabina Sahanic, Ivan Tancevski, Yurii Stepanovskyy, Oksana Boyarchuk, Yoko Nukui, Miyuki Tsumura, Loreto Vidaur, Stuart G. Tangye, Sonia Burrel, Darragh Duffy, Lluis Quintana-Murci, Adam Klocperk, Nelli Y. Kann, Anna Shcherbina, Yu-Lung Lau, Daniel Leung, Matthieu Coulongeat, Julien Marlet, Rutger Koning, Luis Felipe Reyes, Angélique Chauvineau-Grenier, Fabienne Venet, Guillaume Monneret, Michel C. Nussenzweig, Romain Arrestier, Idris Boudhabhay, Hagit Baris-Feldman, David Hagin, Joost Wauters, Isabelle Meyts, Adam H. Dyer, Sean P. Kennelly, Nollaig M. Bourke, Rabih Halwani, Narjes Saheb Sharif-Askari, Karim Dorgham, Jérome Sallette, Souad Mehlal Sedkaoui, Suzan AlKhater, Raúl Rigo-Bonnin, Francisco Morandeira, Lucie Roussel, Donald C. Vinh, Sisse Rye Ostrowski, Antonio Condino-Neto, Carolina Prando, Anastasiia Bonradenko, András N. Spaan, Laurent Gilardin, Jacques Fellay, Stanislas Lyonnet, Kaya Bilguvar, Richard P. Lifton, Shrikant Mane, HGID Lab, COVID Clinicians, COVID-STORM Clinicians, NIAID Immune Response to COVID Group, NH-COVAIR Study Group, Danish CHGE, Danish Blood Donor Study, St. James’s Hospital, SARS CoV2 Interest group, French COVID Cohort Study Group, Imagine COVID-Group, The Milieu Intérieur Consortium, CoV-Contact Cohort, Amsterdam UMC Covid-19 Biobank Investigators, COVID Human Genetic Effort, CONSTANCES cohort, 3C-Dijon Study, Cerba Health-Care, Etablissement du Sang study group, Mark S. Anderson, Bertrand Boisson, Vivien Béziat, Shen-Ying Zhang, Evangelos Vandreakos, Olivier Hermine, Aurora Pujol, Pärt Peterson, Trine H. Mogensen, Lee Rowen, James Mond, Stéphanie Debette, Xavier de Lamballerie, Xavier Duval, France Mentré, Marie Zins, Pere Soler-Palacin, Roger Colobran, Guy Gorochov, Xavier Solanich, Sophie Susen, Javier Martinez-Picado, Didier Raoult, Marc Vasse, Peter K. Gregersen, Lorenzo Piemonti, Carlos Rodríguez-Gallego, Luigi D. Notarangelo, Helen C. Su, Kai Kisand, Satoshi Okada, Anne Puel, Emmanuelle Jouanguy, Charles M. Rice, Pierre Tiberghien, Qian Zhang, Aurélie Cobat, Laurent Abel, Jean-Laurent Casanova

**Affiliations:** 1 Laboratory of Human Genetics of Infectious Diseases, Necker Branch, INSERM U1163, Necker Hospital for Sick Children, Paris, France; 2 University of Paris, Imagine Institute, Paris, France; 3 St. Giles Laboratory of Human Genetics of Infectious Diseases, Rockefeller Branch, The Rockefeller University, New York, NY, USA; 4 Laboratory of Virology and Infectious Disease, The Rockefeller University, New York, NY, USA; 5 Department of Pediatrics, Graduate School of Biomedical and Health Sciences, Hiroshima University, Hiroshima, Japan; 6 Pediatric Infectious Diseases and Immunodeficiencies Unit, Hospital Universitari Vall d’Hebron, Vall d’Hebron Research Institute, Vall d’Hebron Barcelona Hospital Campus, Universitat Autònoma de Barcelona (UAB), Barcelona, Catalonia, Spain; 7 Institute of Biomedicine and Translational Medicine, University of Tartu, Tartu, Estonia; 8 Service de Médecine Intensive Réanimation, Hôpitaux Universitaires Henri Mondor, Assistance Publique-Hôpitaux de Paris (AP-HP); 9 Groupe de Recherche Clinique CARMAS, Faculté de Santé de Créteil, Université Paris Est Créteil, 51, Avenue du Maréchal de Lattre de Tassigny, 94010 Créteil Cedex, France; 10 Avicenne Hospital, Assistance Publique Hôpitaux de Paris, Bobigny, INSERM U1272 Hypoxia & Lung, Bobigny, France; 11 Sorbonne Université, Assistance Publique–Hôpitaux de Paris, Hôpital Pitié–Salpêtrière, Médecine Intensive Réanimation, AP-HP, Paris, France; 12 INSERM UMRS_1166-iCAN, Institute of Cardiometabolism and Nutrition, Paris, France; 13 Internal Medicine Department, Lariboisière Hospital, AP-HP, Paris University, Paris, France; 14 University of Lille, U1019-UMR9017-Center for Infection and Immunity of Lille, Lille, France; 15 CNRS, UMR9017, Lille, France; 16 INSERM, U1019, Lille, France; 17 Institut Pasteur de Lille, Lille, France; 18 CHU Lille, Pôle de Réanimation, Hôpital Roger Salengro, Lille, France; 19 Etablissement Français du Sang, La Plaine-St Denis, France; 20 UMR 1098 RIGHT, Inserm, EFS, Université de Franche-Comté, Besançon, France; 21 SAINBIOSE, INSERM U1059, University of Lyon, Université Jean-Monnet-Saint-Etienne; 22 Etablissement Français du Sang, Auvergne Rhône-Alpes, St-Etienne, St-Etienne, France; 23 Department of Internal Medicine, Infanta Leonor University Hospital, Madrid, Spain; 24 Hospices Civils de Lyon, Lyon, France; International Center of Research in Infectiology, Lyon University, INSERM U1111, CNRS UMR 5308, ENS, UCBL, Lyon, France; 25 Joint Research Unit, Hospices Civils de Lyon-bio Mérieux, Hospices Civils de Lyon, Lyon Sud Hospital, Pierre-Bénite, France; International Center of Research in Infectiology, Lyon University, INSERM U1111, CNRS UMR 5308, ENS, UCBL, Lyon, France; 26 CNRS UMR 5308, ENS, UCBL, Lyon, France; National Referee Centre for Rheumatic, and Autoimmune and Systemic Diseases in Children (RAISE), Lyon, France; Lyon; Immunopathology Federation LIFE, Hospices Civils de Lyon, Lyon, France; 27 Intensive Care Unit, Grand Hôpital de l’Est Francilien Site de Marne-la-Vallée, Jossigny, France; 28 Institut Hospitalo-Universitaire Méditerranée Infection , Marseille, France; 29 Aix Marseille Université, IRD, APHM, MEPHI, Marseille, France; 30 Service d’Evaluation Médicale, Hôpitaux Universitaires de Marseille Assistance Publique Hôpitaux de Marseille (APHM), Marseille, France; 31 Aix Marseille University, School of Medicine - La Timone Medical Campus, EA 3279: CEReSS - Health Service Research and Quality of Life Center, Marseille, France; 32 Laboratory of Clinical Immunology and Microbiology, Division of Intramural Research, NIAID, NIH, Bethesda, MD, USA; 33 Department of Pediatrics and Developmental Biology, Graduate School of Medical and Dental Sciences, Tokyo Medical and Dental University (TMDU), Tokyo, Japan; 34 Department of Epidemiology, Infectious Disease Control and Prevention, Graduate School of Biomedical and Health Sciences, Hiroshima University, Hiroshima, Japan; 35 Hospital Universitari Mutua Tarrassa, Tarrasa, Spain; 36 Paris Cardiovascular Center, PARCC, Inserm, Université de Paris, Paris, France; 37 Service d’Hématologie Biologique, Hôpital Lariboisière, Assistance Publique-Hôpitaux de Paris and EA3518, Institut Universitaire d’Hématologie-Hôpital Saint Louis, Université Paris Diderot, Paris, France; 38 Réanimation Médicale et Toxicologique, Hôpital Lariboisière (AP-HP), Université Paris-Diderot, INSERM Unité Mixte de Recherche Scientifique (UMRS) 1144; 39 Laboratory of Immunobiology, Center for Clinical, Experimental Surgery, and Translational Research, Biomedical Research Foundation of the Academy of Athens, 11527 Athens, Greece; 40 Service de Parasitologie-Mycologie, Groupe Hospitalier Pitié Salpêtrière, AP-HP, Paris, France; 41 Institute for Systems Biology, Seattle, WA 98109, USA; 42 Primary Immunodeficiencies Group, Department of Microbiology and Parasitology, School of Medicine, University of Antioquia UDEA, Medellín, Colombia; 43 Center for Autoimmune Disease Research, School of Medicine and Health Sciences, Universidad del Rosario, Bogota, Colombia; 44 Critical Care Unit , University Hospital of Gran Canaria Dr. Negrín, Canarian Health System, Las Palmas de Gran Canaria, Spain; 45 CIBER de Enfermedades Respiratorias (CIBERES), Instituto de Salud Carlos III, Madrid, Spain; 46 CREA Laboratory, Diagnostic Department, ASST Spedali Civili di Brescia, Brescia, Italy; 47 Pediatric Department and Centro Tettamanti-European Reference Network PaedCan, EuroBloodNet, MetabERN-University of Milano-Bicocca-Fondazione MBBM-Ospedale, San Gerardo, Monza, Italy; 48 Department of Infectious Diseases, San Gerardo Hospital–University of Milano-Bicocca, Monza, Italy; 49 Department of Pediatrics, Fondazione IRCCS Policlinico San Matteo, University of Pavia, Pavia, Italy; 50 NIAID Clinical Genomics Program, National Institutes of Health, Bethesda, USA; 51 Department of Pharmacology & Molecular Therapeutics, Uniformed Services University of the Health Sciences, Bethesda, MD, USA; 52 Department of Pediatrics, British Columbia Children’s Hospital, The University of British Columbia, Vancouver, BC, Canada; 53 Primary Immunodeficiencies Group, University of Antioquia UdeA, Medellin, Colombia; 54 School of Microbiology, University of Antioquia UdeA, Medellin, Colombia; 55 Department of Nephrology and Transplantation, Necker University Hospital - APHP, Paris, France; INEM INSERM U 1151 - CNRS UMR 8253, Paris University, Paris, France; 56 Institute for Biomedical Research, Spanish Research Council, Barcelona, Spain; 57 Institut d’Investigacions Biomèdiques August Pi i Sunyer (IDIBAPS), Barcelona, Spain; 58 Department of Paediatric Immunology and Pulmonology, Center for Primary Immunodeficiency Ghent, Jeffrey Modell Diagnosis and Research Center, Ghent University Hospital, Ghent, Belgium; 59 Faculty of Medical Sciences, University “Goce Delchev”, Stip, Republic of Northern Macedonia; 60 Institute of public health of Republic of North Macedonia; 61 Cancer Genetics and Prevention Program, University of California San Francisco, San Francisco, USA; 62 Department of Molecular Biology and Genetics, Bilkent University, Bilkent - Ankara, Turkey; 63 Meram Medical Faculty, Necmettin Erbakan University, Meram Medical Faculty, Konya, Turkey; 64 Clinical Immunology Unit, Department of Pediatric Infectious Disease, CHU Ibn Rushd and LICIA, Laboratoire d’Immunologie Clinique, Inflammation et Allergie, Faculty of Medicine and Pharmacy, Hassan II University, Casablanca, Morocco; 65 Department of Immunology, CIC1408, GIMAP CIRI INSERM U1111, University Hospital of Saint-Etienne, Saint-Etienne, France; 66 Department of Biosciences and Nutrition, Karolinska Institutet, Stockholm, Sweden; 67 Université de Lille, Inserm, CHU Lille, Institut Pasteur de Lille, U1011- EGID, F-59000 Lille, France; 68 The Genetics Institute, Tel Aviv Sourasky Medical Center, Tel Aviv University, Tel Aviv, Israel; 69 Feinstein Institutes for Medical Research, Northwell Health, Manhasset, NY, USA; 70 Pathogenesis and Therapy of Primary Immunodeficiencies Unit, San Raffaele, Milano, Italy; 71 Diabetes Research Institute, IRCCS San Raffaele Scientific Institute, Milan, Italy; 72 Hematology and Bone Marrow Transplantation Unit, IRCCS San Raffaele Scientific Institute, Milano, Italy; 73 Department of Immunology, Institute of Biomedical Sciences, University of Sao Paulo, Sao Paulo, Brazil; 74 Fundación Rioja Salud - Centro de Investigación Biomédica de La Rioja, Logrono, Spain; 75 University of Sao Paulo, Sao Paulo, Brazil; 76 Department of Neurology, Amsterdam Neuroscience, Amsterdam, The Netherlands; 77 Department of Internal Medicine II, Medical University of Innsbruck, Innsbruck, Austria; 78 Shupyk National Healthcare University of Ukraine, Kyiv, Ukraine; 79 Department of Children’s Diseases and Pediatric Surgery, I.Horbachevsky Ternopil National Medical University, Ternopil, Ukraine; 80 Department of Infection Control and Prevention, Medical Hospital, Tokyo Medical and Dental University (TMDU), Tokyo, Japan; 81 Intensive Care Department, Donostia University Hospital, San Sebastian, Spain; 82 Centro de Investigación en Red de Enfermedades Respiratorias-CIBERES - Instituto de Salud Carlos III, Madrid, España; 83 Garvan Institute of Medical Research, Sydney, Australia; 84 Sorbonne Université, INSERM U1136, Institut Pierre Louis d’Epidémiologie et de Santé Publique (iPLESP), AP-HP, Hôpital Pitié Salpêtrière, Service de Virologie, Paris, France; 85 Translational Immunology Lab, Institut Pasteur; 86 Human Evolutionary Genetics Unit, Institut Pasteur, CNRS UMR 2000, Paris, France; 87 Chair of Human Genomics and Evolution, Collège de France, Paris, France; 88 Department of Immunology, 2nd Faculty of Medicine, Charles University and University Hospital in Motol, Prague, Czech Republic; 89 Dmitry Rogachev National Medical Research Center of Pediatric Hematology, Oncology and Immunology, Moscow, Russia; 90 Department of Paediatrics & Adolescent Medicine, The University of Hong Kong, Hong Kong, China; 91 Division of Geriatric Medicine, Tours University Medical Center, Tours, France; 92 INSERM U1259, MAVIVH, Université de Tours, Tours, France; 93 Service de Bactériologie-Virologie-Hygiène, CHU de Tours, Tours, France; 94 Department of Microbiology, Universidad de La Sabana, Chia, Colombia; 95 Department of Critical Care Medicine, Clinica Universidad de La Sabana, Chia, Colombia; 96 Service de Biologie Médicale, CHI Robert Ballanger, Aulnay sous Bois, France; 97 Laboratoire d’Immunologie, Hospices Civils de Lyon, Hôpital Edouard Herriot, Lyon, France; 98 Centre International de Recherche en Infectiologie (CIRI), Inserm U1111, CNRS, UMR5308, Ecole Normale Supérieure de Lyon, Université Claude Bernard-Lyon 1, Lyon, France; 99 EA 7426 « Pathophysiology of Injury-Induced Immunosuppression », Université Claude Bernard Lyon 1 - Hospices Civils de Lyon, Hôpital Edouard Herriot - BioMérieux, Lyon, France; 100 Laboratory of Molecular Immunology, Rockefeller University, New York, NY, USA; 101 Howard Hughes Medical Institute, New York, NY, USA; 102 Sackler Faculty of Medicine, Tel Aviv University, Tel Aviv, Israel; 103 Allergy and Clinical Immunology Unit, Department of Medicine, Tel Aviv Sourasky Medical Center, Tel Aviv, Israel; 104 Medical Intensive care Unit, University Hospitals Leuven, Leuven, Belgium; 105 Laboratory of Inborn Errors of Immunity, Department of Immunology, Microbiology and Transplantation, KU Leuven, Leuven, Belgium; 106 Department of Pediatrics, Jeffrey Modell Diagnostic and Research Network Center, University Hospitals Leuven, Leuven, Belgium; 107 Department of Age-Related Healthcare, Tallaght University Hospital & Department of Medical Gerontology, School of Medicine, Trinity College Dublin; 108 Department of Medical Gerontology, School of Medicine, Trinity College Dublin; 109 Sharjah Institute for Medical Research, College of Medicine, University of Sharjah, Sharjah, United Arab Emirates; 110 Sorbonne Université, Inserm, Centre d’Immunologie et des Maladies Infectieuses, (CIMI- Paris), Paris, France; 111 Cerba Health Care, Issy-les-Moulineaux, France; 112 Department of Pediatrics, King Fahad Hospital of the University, Al-Khobar, Saudi Arabia; 113 College of Medicine, Imam Abdulrahman Bin Faisal University, Dammam, Saudi Arabia; 114 Department of Clinical Laboratory, Hospital Universitari de Bellvitge, IDIBELL, Barcelona, Spain; 115 Department of Immunology, Hospital Universitari de Bellvitge, IDIBELL, Barcelona, Spain; 116 Department of Medicine, Division of Infectious Diseases, McGill University Health Centre, Montréal, Québec, Canada; 117 Infectious Disease Susceptibility Program, Research Institute-McGill University Health Centre, Montréal, Québec, Canada; 118 Department of Clinical Immunology, Rigshospitalet, Copenhagen University Hospital, Copenhagen, Denmark; 119 Faculdades Pequeno Príncipe, Instituto de Pesquisa Pelé Pequeno Príncipe, Curitiba, Brazil; 120 Department of Medical Microbiology, University Medical Center Utrecht, Utrecht, Netherlands; 121 Service de Médecine Interne, Hôpital universitaire Jean-Verdier, AP-HP, Bondy, France; 122 INSERM U1138, Centre de Recherche des Cordeliers, Paris, France; 123 School of Life Sciences, Ecole Polytechnique Fédérale de Lausanne, Lausanne, Switzerland; 124 Precision Medicine Unit, Lausanne University Hospital and University of Lausanne, Lausanne, Switzerland; 125 Swiss Institue of Bioinformatics, Lausanne, Switzerland; 126 Imagine Institute, Université de Paris, INSERM UMR 1163, Paris, France; 127 Yale Center for Genome Analysis, Yale School of Medicine, New Haven, CT, USA; 128 Department of Genetics, Yale University School of Medicine, New Haven, CT, USA; 129 Department of Neurosurgery, Yale University School of Medicine, New Haven, CT, USA; 130 Department of Medical Genetics, Acibadem University School of Medicine, Istanbul, Turkey; 131 Department of Genetics, Yale University School of Medicine, New Haven, CT, USA; 132 Laboratory of Human Genetics and Genomics, The Rockefeller University, New York, NY; 133 Diabetes Center, University of California, San Francisco, CA, USA; 134 Center for Clinical, Experimental Surgery and Translational Research, Biomedical Research Foundation of the Academy of Athens, Athens, Greece; 135 Department of Hematology, Necker Hospital, AP-HP, Paris, France; 136 Neurometabolic Diseases Laboratory, IDIBELL-Hospital Duran i Reynals, CIBERER U759, and Catalan Institution of Research and Advanced Studies (ICREA), Barcelona, Spain; 137 Department of Infectious Diseases, Aarhus University Hospital, Skejby, Denmark; 138 Department of Biomedicine, Aarhus University, Aarhus, Denmark; 139 ADMA Biologics Inc., Ramsey NJ; 140 University of Bordeaux, INSERM, Bordeaux Population Health Center, UMR1219, F-33000 Bordeaux, France; 141 Bordeaux University Hospital, Department of Neurology, Institute of Neurodegenerative Diseases, F-33000 Bordeaux, France; 142 IHU Méditerranée Infection, Unité des Virus Émergents, UVE: Aix Marseille University, IRD 190, INSERM 1207, Marseille, France; 143 Inserm CIC 1425, Paris, France; 144 Université de Paris, IAME UMR-S 1137, INSERM, Paris, France; 145 AP-HP, Département Epidémiologie Biostatistiques et Recherche Clinique, Hôpital Bichat, Paris, France; 146 AP-HP, Bichat Claude Bernard Hospital, Infectious and Tropical Diseases Department, Paris, France; 147 Université de Paris, Université Paris-Saclay, UVSQ, Inserm UMS11, Villejuif, France; 148 Immunology Division, Genetics Department, Hospital Universitari Vall d’Hebron, Vall d’Hebron Research Institute, Vall d’Hebron Barcelona Hospital Campus, UAB, Barcelona, Catalonia, Spain; 149 Département d’Immunologie, Assistance Publique Hôpitaux de Paris (AP-HP), Hôpital Pitié-Salpétrière, Paris, France; 150 Department of Internal Medicine, Hospital Universitari de Bellvitge, IDIBELL, Barcelona, Spain; 151 IrsiCaixa AIDS Research Institute and Institute for Health Science Research Germans Trias i Pujol (IGTP), Badalona, Spain; 152 Infectious Diseases and Immunity, Center for Health and Social Care Research (CESS), Faculty of Medicine, University of Vic-Central University of Catalonia (UVic-UCC), Vic, Spain; 153 Catalan Institution for Research and Advanced Studies (ICREA), Barcelona, Spain; 154 Service de Biologie Clinique & UMR-S 1176, Hopital Foch, Suresnes, France; 155 Hospital Universitario de Gran Canaria Dr Negrín, Canarian Health System, Canary Islands, Spain; 156 Department of Clinical Sciences, University Fernando Pessoa Canarias, Las Palmas de Gran Canaria, Canary Islands, Spain; 157 Department of Pathology and Laboratory Medicine, Perelman School of Medicine, University of Pennsylvania, Philadelphia, PA

## Abstract

Circulating autoantibodies (auto-Abs) neutralizing high concentrations (10ng/mL, in plasma diluted 1 to 10) of IFN-α and/or -ω are found in about 10% of patients with critical COVID-19 pneumonia, but not in subjects with asymptomatic infections. We detect auto-Abs neutralizing 100-fold lower, more physiological, concentrations of IFN-α and/or -ω (100pg/mL, in 1/10 dilutions of plasma) in 13.6% of 3,595 patients with critical COVID-19, including 21% of 374 patients > 80 years, and 6.5% of 522 patients with severe COVID-19. These antibodies are also detected in 18% of the 1,124 deceased patients (aged 20 days-99 years; mean: 70 years). Moreover, another 1.3% of patients with critical COVID-19 and 0.9% of the deceased patients have auto-Abs neutralizing high concentrations of IFN-β. We also show, in a sample of 34,159 uninfected subjects from the general population, that auto-Abs neutralizing high concentrations of IFN-α and/or -ω are present in 0.18% of individuals aged between 18 and 69 years, 1.1% between 70 and 79 years, and 3.4% > 80 years. Moreover, the proportion of subjects carrying auto-Abs neutralizing lower concentrations is greater in a subsample of 10,778 uninfected individuals: 1% of individuals < 70 years, 2.3% between 70 and 80 years, and 6.3% > 80 years. By contrast, auto-Abs neutralizing IFN-β do not become more frequent with age. Auto-Abs neutralizing type I IFNs predate SARS-CoV-2 infection and sharply increase in prevalence after the age of 70 years. They account for about 20% of both critical COVID-19 cases in the over-80s, and total fatal COVID-19 cases.

## One-sentence summary

Autoantibodies neutralizing type I IFNs increase in prevalence over 60 years of age and underlie about 20% of all fatal COVID-19 cases.

## Introduction

Since the start of the COVID-19 pandemic in December 2019, more than 200 million people have been infected with SARS-CoV-2, resulting in at least 4 million deaths, and probably closer to 7 to 9 million deaths worldwide. Interindividual clinical variability in the course of acute infection is vast, extending from silent or mild infection in about 90% of subjects to pneumonia and respiratory failure, both requiring hospitalization, in less than 10% and 2% of cases, respectively. Age is the major epidemiological risk factor for hospitalization or death from pneumonia, the risk doubling with every five years of age (1, 2). The frequencies of critical disease and death from COVID-19 are higher in men than in women (3–5). With the COVID Human Genetic Effort (6), we previously reported that inborn errors of TLR3- and IRF7-dependent type I IFN induction and amplification can underlie life-threatening COVID-19 pneumonia in a small subset of patients (7, 8). Autosomal dominant disorders were found in 19 patients, but our cohort also included four previously healthy unrelated adults aged 25 to 50 years with autosomal recessive, complete IRF7 (*N*=2) or IFNAR1 (*N*=2) deficiency. These findings indicated that type I IFN immunity is essential for protective immunity to respiratory infection with SARS-CoV-2 but surprisingly redundant otherwise. We also reported that an autoimmune phenocopy of inborn errors of type I IFN-dependent immunity can underlie critical COVID-19 pneumonia (9). Indeed, autoantibodies (auto-Abs) neutralizing 10ng/mL IFN-α2 and/or -ω were found in the blood of at least 10% of an international cohort of patients with life-threatening COVID-19 pneumonia, but in none of the tested individuals with asymptomatic or paucisymptomatic infection (9). These auto-Abs were detected in serum or plasma diluted 1/10. The auto-Abs in the patients’ undiluted blood can therefore probably neutralize as much as 100ng/mL IFN-α2 and/or -ω. The 17 subtypes of type I IFNs, including 13 IFN-α subtypes, IFN-ω, IFN-β, IFN-ε, and IFN-κ, bind to the same heterodimeric receptor (IFNAR1 and IFNAR2). (10). The 13 IFN-α subtypes and IFN-ω are closely related phylogenetically, while IFN-β, IFN-ε, and IFN-κ are more distant (9). The auto-Abs to IFN-α2 and/or -ω were mostly found in men (95%) and in the elderly (half the patients with antibodies being over the age of 65 years) (9). These findings were later replicated in independent cohorts from Amsterdam, Lyon, Madrid, New Haven, and San Francisco (11–16).

These auto-Abs against type I IFNs were found in about 0.3% of a general population sample of 1,227 subjects collected before the pandemic and aged 20 to 69 years, suggesting that they predated SARS-CoV-2 infection and caused critical COVID-19 rather than being triggered by it (9). Moreover, production of these antibodies can be genetically driven, and can begin during early childhood, as attested by their presence in almost all patients with autoimmune polyendocrine syndrome type-1 (APS-1) due to germline mutations of *AIRE* (17–19). APS-1 patients are, indeed, at very high risk of developing severe or critical COVID-19 pneumonia (20, 21). These auto-Abs are also found in patients with combined immunodeficiency and hypomorphic mutations of *RAG1* or *RAG2* (22), in men with immunodysregulation polyendocrinopathy enteropathy X-linked (IPEX) and mutations of *FOXP3* (23), and in women with incontinentia pigmenti and heterozygous null mutations of X-linked *NEMO* (9). They are also seen in patients treated with IFN-α or IFN-β (24, 25), in patients with systemic lupus erythematosus (26, 27), thymoma (28), or with myasthenia gravis (29, 30). Finally, they underlie a third of adverse reactions to the 17D live attenuated vaccine against yellow fever virus (YFV), further suggesting that they were present in these patients, as in patients with critical COVID-19, before viral infection (31). Remarkably, for all patients tested, the auto-Abs neutralized the protective effect of ~400 pg/mL IFN-α2 against SARS-CoV-2 or YFV-17D *in vitro*, even when the plasma was diluted by >1/1,000 (9). As blood IFN-α concentrations during acute asymptomatic or paucisymptomatic SARS-CoV-2 infection typically range from 1 to 100pg/mL (32, 33), and IFN-α levels in the respiratory tract might be even lower yet protective, we hypothesized that auto-Abs neutralizing concentrations of type I IFNs below 10ng/mL may underlie life-threatening COVID-19 pneumonia in more than 10% of cases. We also hypothesized that the prevalence of auto-Abs against type I IFNs in the general, uninfected, population may increase with age and that these antibodies may be more common in men than in women.

## Results

### High and intermediate levels of IgG auto-Abs against IFN-α2 and/or IFN-ω in ~20% of patients with critical COVID-19

**Fig. 1.**
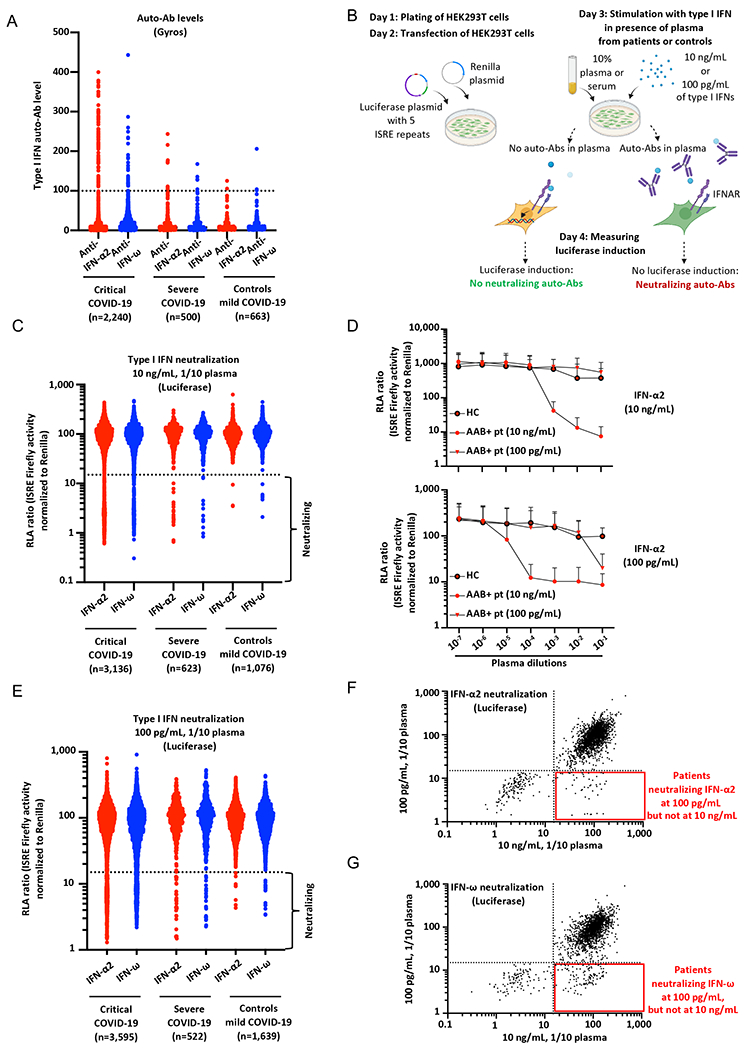
Neutralizing auto-Abs against IFN-α2 and/or IFN-ω in patients with life-threatening COVID-19. **(A)** Gyros (high-throughput automated ELISA) results for auto-Abs against IFN-α2 and/or IFN-ω in patients with critical COVID-19 (*N*=2,240), severe COVID-19 (*N*=500), or asymptomatic/mild SARS-CoV-2 infection (*N*=663). **(B)** Schematic representation of the neutralization assay developed in HEK293T cells, using a luciferase system. ISRE: interferon-sensitive response elements. **(C)** Results for the neutralization of 10ng/mL IFN-α2 or IFN-ω in the presence of plasma 1/10 from patients with critical COVID-19 (*N*=3,136), severe COVID-19 (*N*=623), or controls with mild/asymptomatic infection (*N*=1,076). Relative luciferase activity is shown (ISRE dual luciferase activity, with normalization against *Renilla* luciferase activity) after stimulation with 10ng/mL IFN-α2 or IFN-ω in the presence of plasma 1/10. RLA: relative luciferase activity. **(D)** RLA after stimulation with IFN-α2 at a concentration of 10ng/mL or 100pg/mL, with various dilutions of plasma from a positive control (from 1/10 to 1/10^7^) neutralizing 10ng/mL of type I IFNs (AAB+ pt, 10ng/mL), a patient neutralizing 100pg/mL of type I IFNs but not 10ng/mL (AAB+ pt, 100pg/mL), and a healthy control (HC). AAB: auto-Ab. Pt: patient. **(E)** Neutralization of 100pg/mL IFN-α2 or IFN-ω in the presence of plasma 1/10 from patients with critical COVID-19 (*N*=3,595), severe COVID-19 (*N*=522), or controls with asymptomatic/mild infection (*N*=1,639). **(F)** Plot showing luciferase induction after stimulation with 10ng/mL or 100pg/mL IFN-α2, in the presence of plasma from patients with critical COVID-19. Dotted lines indicate neutralizing levels, defined as induction levels below 15% of the mean value for controls tested the same day. Patients with antibodies neutralizing both 10ng/mL and 100pg/mL IFN-α2 are shown in the bottom left corner, whereas the patients in the bottom right corner had antibodies capable of neutralizing only 100pg/mL IFN-α2. **(G)** Plot showing luciferase induction after stimulation with 10ng/mL or 100pg/mL IFN-ω, for patients with critical COVID-19.

We recruited a cohort of 3,595 patients hospitalized with critical COVID-19 pneumonia (hereafter referred to as “critical patients”, and defined as pneumonia in patients with critical disease, including (i) pulmonary, with high-flow oxygen (> 6L/min) or mechanical ventilation (continuous positive airway pressure, bilevel positive airway pressure, intubation), (ii) cardiovascular shock, or (iii) any other organ failure requiring admission to an intensive care unit), including 566 patients of our previously described cohort of 987 patients with critical COVID-19 pneumonia for whom residual samples were available (9), 623 individuals with severe COVID-19 pneumonia (with less than 6 L of oxygen supplementation, hereafter referred to as “severe patients”), and 1,639 individuals with asymptomatic or paucisymptomatic (mild) upper respiratory tract SARS-CoV-2 infection (the “controls”, infected with SARS-CoV-2 (as demonstrated by a positive PCR and/or serological test and/or displaying typical manifestations, such as anosmia/ageusia after exposure to a confirmed COVID-19 case) who remained asymptomatic or developed mild, self-healing, ambulatory disease with no evidence of pneumonia), including 427 samples from the initial control cohort of 663 individuals (9). The patients originated from 38 different countries, across all continents. We did not include patients with moderate pneumonia, who did not receive oxygen therapy (7, 9). We searched for auto-Abs against IFN-α2 and -ω, by establishing novel, sensitive, and robust assays for the detection of circulating IgG auto-Abs. We used Gyros technology (34), a high-throughput automated enzyme-linked immunosorbent assay (ELISA)-like assay capable of detecting a large range of auto-Ab levels (Fig. S1A). We confirmed that the Gyros technique was as sensitive as the techniques previously used (ELISA and Luminex), and that all tested patients with high levels of anti-IFN-α2 and/or anti-IFN-ω auto-Abs on ELISA, as reported in our previous studies (defined as an optical density > 0.5) had high levels of auto-Abs when assessed with Gyros (defined as levels >100) (Fig. S1B). We then screened newly recruited critical or severe patients and controls from our COVID-19 cohort (Fig. 1A). We found high levels of anti-IFN-α2 and/or anti-IFN-ω auto-Abs in 6.9% of critical patients, 3.4% of patients with severe COVID-19, and only 0.6% of the asymptomatic or paucisymptomatic controls (Figure 1A). We also found that another 12.7% of patients with critical COVID-19 had intermediate levels of anti-IFN-α2 and/or IFN-ω auto-Abs in Gyros assays (defined as levels >30 and <100, based on the distribution observed in healthy controls), whereas this was the case for 8.6% of patients with severe COVID-19 and 11% of the individuals in our control cohort. Collectively, these findings replicate and extend our previous results and those of other groups (9, 11–15, 35), while suggesting that intermediate levels of auto-Abs against type I IFNs might be neutralizing and underlie critical disease.

### Auto-Abs neutralizing 10ng/mL IFN-α2 and/or -ω in almost 10% of the critical patients

We investigated the ability of these auto-Abs to neutralize high concentrations of type I IFNs, as defined in our previous reports (10ng/mL IFN-α2 or IFN-ω in medium containing 1/10 plasma or serum, the equivalent of 100ng/mL IFN-α2 or IFN-ω in undiluted plasma). We tested not only the patients with high levels of auto-Abs, as in our previous study (9), but all the available patients with critical COVID-19 (*N*=3,136), or severe COVID-19 (*N*=623), and controls (*N*=1,076) from our expanded cohort. We designed a high-throughput luciferase assay in which we transfected human embryonic kidney (HEK)293T cells with i) a plasmid containing five IFN-stimulated response element (ISRE) repeats and a firefly luciferase reporter, and ii) a plasmid encoding the *Renilla* luciferase. We stimulated these cells with an individual recombinant type I IFN (IFN-α2 or IFN-ω), in the presence of plasma diluted 1/10 (plasma 1/10) from patients or controls. We then measured firefly luciferase induction, normalized against *Renilla* luciferase activity (Fig. 1B). We confirmed the robustness of this assay by comparing the results with our previous pSTAT1 flow cytometry data (9). Consistent results were obtained for all 50 patients tested with both techniques (Fig. S1C, D). We then tested all patients and controls. Most plasma samples with high auto-Ab levels (>100) against IFN-α2 according to the Gyros assay were neutralizing (Fig. S1E). We found that 9.8% (307 of 3,136) of the critical patients tested and 3.53% (22 of 623) of the severe patients had auto-Abs neutralizing IFN-α2 and/or IFN-ω, versus only 0.37% (4 of 1,076) controls (Fig. 1C) (Table 1 and Table S1). In the patients with neutralizing auto-Abs, these auto-Abs were able to neutralize both IFN-α2 and IFN-ω in 175 of the 307 critical patients (57%), 6 of the severe patients (27%), and none of the controls; IFN-α2 alone in 106 critical patients (34.5%), 11 severe patients (50%), and only one of the controls (25%); IFN-ω alone in 26 of critical patients (8.5%), 5 severe patients (22%), and 3 controls (75%) (Table S1). None of the patients with these auto-Abs had inborn errors of TLR3- or TLR7-dependent type I IFN immunity (7, 36).

### Auto-Abs neutralizing 100pg/mL IFN-α2 and/or -ω in at least 13.6% of critical patients and 6.8% of severe patients

As the amounts of circulating type I IFNs in infected individuals are 100 to 1,000 times lower than the amounts tested previously (32, 33), we investigated the neutralization of more physiological concentrations of type I IFNs, by performing assays with 100pg/mL type I IFN. We observed a robust response in our luciferase system, in the presence of 1/10 dilutions of control plasma (Fig. S1F). The plasma or serum was diluted 1/10, so the concentration neutralized corresponds to 1ng/mL IFN in circulating whole blood. With diluted plasma samples from a positive control, we gained at least two orders of magnitude of sensitivity in terms of neutralizing activity, providing proof-of-concept that these auto-Abs can neutralize lower, more physiological, amounts of type I IFNs (Fig. 1D, Fig. S1G), lower than the concentrations previously tested by a factor of 100 (9). We then retested all available samples from our extended cohort. Overall, 13.6% of all critical patients tested (*N*=489 of 3,595), 6.5% (*N*=34 of 522) of the severe patients, and 1% of the controls (*N*=17 of 1,639) had circulating auto-Abs that neutralized 100pg/mL IFN-α2 and/or IFN-ω in plasma 1/10 (Fig. 1E–G) (Table 1 and Table S1). In the patients with neutralizing auto-Abs, these auto-Abs were able to neutralize both IFN-α2 and IFN-ω in 256 of the 489 positive critical patients (52%), 18 of the 34 severe patients (53%), and 1 of the 17 controls (6%); IFN-α2 alone in 104 critical patients (21%), 14 severe patients (41%), and 4 of the controls (23.5%); IFN-ω alone in 129 critical patients (26%), 2 severe patients (6%), and 12 controls (70%) (Table S1). Further dilution of a plasma sample from one patient neutralizing 100pg/mL of type I IFNs led to a loss of neutralizing activity (Fig. 1D, Fig. S1G). Importantly, for four unrelated patients, all of whom suffered from critical COVID-19, including one who died, samples collected before COVID-19 were available and tested positive for neutralizing auto-Abs against type I IFNs. One neutralized IFN-α2 and IFN-ω at a concentration of 10ng/mL, two neutralized both cytokines at 100pg/mL and one IFN-ω only at 100pg/mL (Fig. S1H). The four patients tested therefore had auto-Abs neutralizing 10ng/mL or 100pg/mL IFN-α2 and/or -ω before infection with SARS-CoV-2. These four patients, and another two reported in our previous study (9) all, therefore, had auto-Abs neutralizing type I IFNs before infection with SARS-CoV-2. We then assessed the risk, adjusted for age and sex, of having critical or severe disease for subjects carrying auto-Abs against each individual IFN and the different possible combinations. We found that all auto-Abs, except those neutralizing only IFN-ω at a concentration of 10ng/mL, were highly significant risk factors in comparisons of patients with critical or severe COVID-19 with controls (Table 1 and Table S2). The strongest association was with auto-Abs against both IFN-α2 and IFN-ω neutralizing concentrations of 10ng/mL (OR=67, *P*=8x10^−13^) and 100pg/mL (OR=54, *P*<10^−13^), followed by those against IFN-α2 +/− IFN-ω neutralizing 10ng/mL (OR=45, *P*<10^−13^) and 100pg/mL (OR=23, *P*<10^−13^) (Table 1). As the serum/plasma samples were diluted 1/10 in these assays, these findings suggest that more than 13.6% of patients with life-threatening COVID-19 have circulating auto-Abs neutralizing 1ng/mL IFN-α2 and/or IFN-ω *in vivo*, a greater proportion than the 10% of patients with auto-Abs neutralizing 100ng/mL reported in previous studies (9, 11–15, 35).

### Auto-Abs neutralize low concentrations of IFN-α2 protective against SARS-CoV-2

**Fig. 2.**
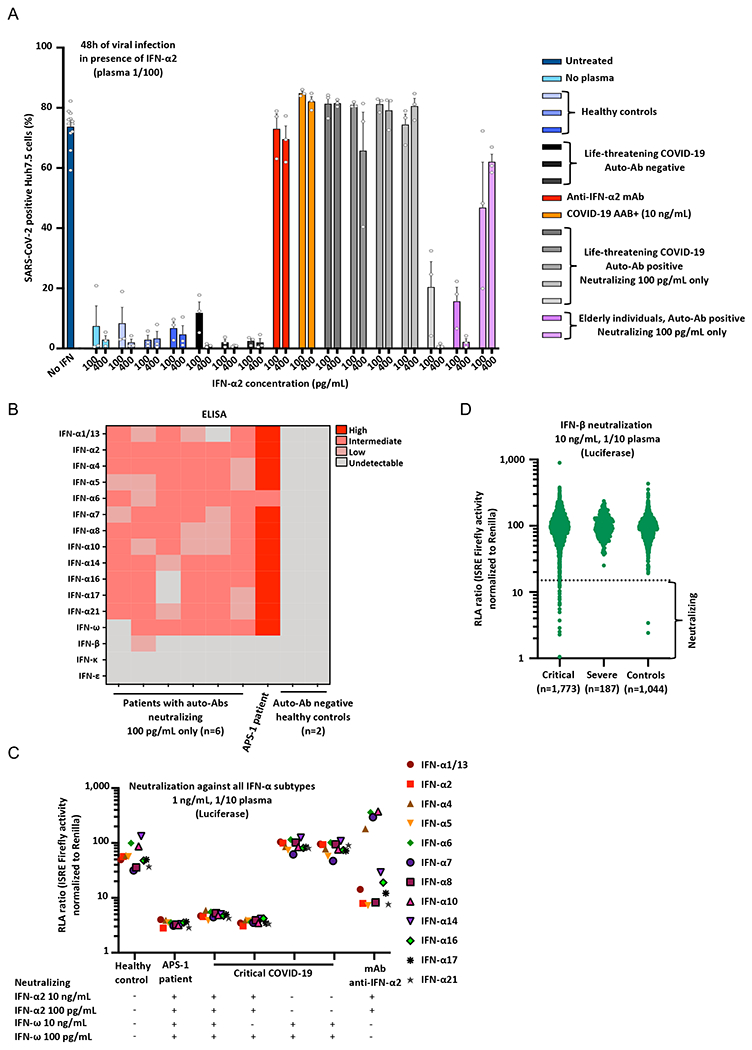
Enhanced SARS-CoV-2 replication, despite the presence of IFN-α2, in the presence of plasma from patients with auto-Abs neutralizing 100pg/mL IFN-α2. **(A)** SARS-CoV-2 replication in Huh-7.5 cells untreated (in dark blue), or treated with ~100 pg/mL or ~400 pg/mL IFN-α2 in the presence of 1/100 plasma from healthy controls without auto-Abs (*N*=3, in blue), from patients with life-threatening COVID-19 but without auto-Abs against IFN-α2 (*N*=3, in black), a commercial anti–IFN-α2 antibody (mAb, in red); from a patient with life-threatening COVID-19 and auto-Abs neutralizing 10ng/mL IFN-α2 in plasma 1/100 (COVID-19 AAB+, *N*=1, in orange), from patients with life-threatening COVID-19 and auto-Abs neutralizing 100pg/mL IFN-α2 in plasma 1/100 (*N*=5, in grey); elderly individuals with auto-Abs neutralizing 100pg/mL IFN-α2 in plasma 1/100 (*N*=2, in purple). Each dot represents a technical replicate. All experiments were done in triplicate. **(B)** ELISA (enzyme-linked immunosorbent assay) for auto-Abs against the 13 IFN-α forms, IFN-ω, IFN-β, IFN-ε, and IFN-κ in patients with life-threatening COVID-19 and auto-Abs neutralizing 100pg/mL IFN-α2 (*N*=6), APS-1 patient with life-threatening COVID-19 and auto-Abs neutralizing 10 ng/mL IFN-α2 and IFN-ω (*N*=1), and healthy controls (*N*=2). **(C)** RLA after stimulation with the all individual IFN-α at a concentration of 1ng/mL, with 1/10 plasma from a healthy control (negative control), an APS-1 patient (positive control), patients with life-threatening COVID-19 and neutralizing IFN-α2 and/or IFN-ω, or a monoclonal antibody anti-IFN-α2. **(D)** Neutralization of 10ng/mL IFN-β in the presence of plasma 1/10 from patients with critical COVID-19 (*N*=1,773), severe COVID-19 (*N*=187), or asymptomatic/mild controls (*N*=1,044).

We previously reported that plasma diluted 1/100 from patients with auto-Abs against type I IFNs neutralized the ability of IFN-α2 (at a concentration of 20 pM, approximately 400pg/mL) to block SARS-CoV-2 and YFV-17D replication in Huh-7.5 cells (9, 31). Strikingly, this neutralization was seen in all patients tested, even for a 1,000-fold dilution, and, in most patients, it was more potent than the neutralizing effect of a commercially available neutralizing monoclonal Ab (mAb) against IFN-α2. These auto-Abs against type I IFNs were, therefore, able to neutralize IFN-α2 at concentrations well beyond physiological levels. We therefore hypothesized that patients with lower titers of auto-Abs against type I IFNs, which can neutralize 100pg/mL but not 10ng/mL in plasma diluted 1/10, would also neutralize the protective effect of IFN-α2 against SARS-CoV-2. We therefore performed our SARS-CoV-2 assay with 5 pM (~100pg/mL) or 20 pM (~400pg/mL) IFN-α2, on five samples from patients with life-threatening COVID-19 and two samples from uninfected elderly individuals with auto-Abs neutralizing 100pg/mL but not 10ng/mL IFN-α2. As controls, we tested a commercial mAb against IFN-α2, a sample from a patient with auto-Abs neutralizing 10ng/mL IFN-α2, and samples from three patients with life-threatening COVID-19 and three healthy controls without detectable auto-Abs against type I IFNs. We found that the 1/100 dilutions of plasma from four of the five critical COVID-19 patients and one of the two elderly individuals with auto-Abs neutralizing 100pg/mL IFN-α2 were able to neutralize the protective effect of ~400 pg/mL IFN-α2 against SARS-CoV-2, whereas samples from all these individuals fully or partially neutralized ~100pg/m IFN-α2 (Fig. 2A). No such neutralizing effect was observed for any of the auto-Ab-negative controls. Overall, our findings indicate that auto-Abs against type I IFNs capable of neutralizing 100pg/mL IFN in 1% plasma can block the protective effect of ~100pg/mL or ~400pg/mL IFN-α2 against SARS-CoV-2. These findings raise the possibility that even 100-fold lower levels of auto-Abs against type I IFNs, capable of neutralizing lower, physiological concentrations of 10pg/mL IFN-α2, may be present in an even larger proportion of patients. The testing of this hypothesis will require the development of new, more sensitive methods to screen for neutralization.

### Neutralization of type I IFNs in the absence of detectable auto-Abs against IFN-α2 or -ω

The neutralization assays performed on all patients and controls revealed that some patients with neutralizing activity against 10ng/mL IFN-α2 and/or IFN-ω, as shown in luciferase assays, did not have high, or even intermediate levels of IgG auto-Abs in Gyros assays (Fig. S1E). We also observed that some patients with neutralizing auto-Abs had low or undetectable levels of auto-Abs in Luminex assays (Fig. S1I). For these individuals, we assessed the prevalence of IgA and IgM auto-Abs against type I IFNs; we found that none of the patients tested (*N*=12) had detectable titers of IgA or IgM auto-Abs (Fig. S1J). We then tested the alternative hypothesis that these auto-Abs were directed against the IFNAR1 or IFNAR2 chain of type I IFN receptors, assessing the ability of plasma samples from these patients to neutralize IFN-β. None of the samples from these patients neutralized IFN-β, suggesting that the auto-Abs in these patients were not directed against IFNAR1 or IFNAR2 (Fig. S1K). An alternative plausible hypothesis is that the epitope recognized by the auto-Abs might be concealed by the binding of the cytokine to the plate (ELISA), biotinylation of the cytokine (Gyros), or covalent coupling of the cytokine to magnetic beads at lysine residues (Luminex) (19). This observation has important clinical implications, suggesting that a lack of detection of auto-Abs against type I IFNs does not rule out the possibility of such antibodies being present and having neutralization capacity.

### Auto-Abs typically neutralize the 13 IFN-α subtypes and/or IFN-ω

In six patients with auto-Abs neutralizing 100pg/mL but not 10ng/mL IFN-α2 and/or IFN-ω, we tested the reactivity of the antibodies against the 17 type I IFNs (the 13 IFN-α forms, IFN-ω, IFN-β, IFN-ε, and IFN-κ). Like patients with auto-Abs neutralizing 10ng/mL type I IFNs (9), those capable of neutralizing only 100pg/mL had detectable auto-Abs against most of the 13 IFN-α forms and/or IFN-ω, albeit at lower levels (Fig. 2B). Of the six patients with auto-Abs against IFN-α and/or IFN-ω tested, only one also had auto-Abs against IFN-β and none had detectable auto-Abs against IFN-ε or IFN-κ. Overall, the patients with auto-Abs against IFN-α2 and/or IFN-ω capable of neutralizing 100pg/mL IFN displayed patterns of reactivity to the 17 type I IFNs similar to those reported in previously described patients with auto-Abs neutralizing 10ng/mL (9). We then set up an assay for assessing neutralization of the 13 IFN-α forms, using our luciferase-based assay. We tested two patients with auto-Abs neutralizing IFN-α2 and IFN-ω, two patients with auto-Abs neutralizing only IFN-α2, and two patients with auto-Abs neutralizing only IFN-ω. Interestingly, we found that the APS-1 patient, and the two patients with auto-Abs neutralizing 10ng/mL IFN-α2 and IFN-ω were able to neutralize all 13 IFN-α subtypes, as were the two patients with neutralizing auto-Abs against IFN-α2. Conversely, in the conditions tested, the two patients with auto-Abs neutralizing IFN-ω only, but not IFN-α2, were not able to neutralize any of the 13 IFN-α subtypes (Fig. 2C). In addition, to confirm that the IgG auto-Abs detected were indeed the cause of the neutralization activity observed, we performed an IgG depletion experiment and found that the removal of the IgG fraction abolished the neutralizing activity, whereas the purified IgG fraction had full neutralizing activity (Fig. S2A). Thus, patients with neutralizing auto-Abs against only IFN-ω do not seem to neutralize any of the 13 IFN-α subtypes, whereas patients with auto-Abs neutralizing IFN-α2 neutralize all these subtypes.

### Auto-Abs neutralizing IFN-β in 1.3% of critical patients

We previously reported that auto-Abs neutralizing IFN-β were detected in only two of 101 critical patients with auto-Abs neutralizing 10ng/mL IFN-α2 and/or IFN-ω (9). Given the potential therapeutic use of IFN-β (37, 38), and the absence of IFN-β-neutralization data for COVID-19 patients, we tested a larger number of patients and controls, including patients without auto-Abs against IFN-α or IFN-ω, for auto-Abs against IFN-β, assessing the levels and neutralizing activity of auto-Abs against 10ng/mL IFN-β. We screened 1,773 patients with critical COVID-19 pneumonia, and found that 1.3% (*N*=23) had neutralizing auto-Abs against IFN-β; by contrast, such antibodies were present in none of the 187 severe patients tested and in only two of the 1,044 controls tested (0.18%) (Fig. 2D, S2B and Table S3). Interestingly, only six of the 23 (21.7%) critical patients also had auto-Abs neutralizing IFN-α2 and/or IFN-ω at 100pg/mL, and none of the controls had such antibodies. Of note, five of these six patients had auto-Abs neutralizing all three cytokines. All the other critical patients and controls had only neutralizing auto-Abs against IFN-β. The presence of neutralizing auto-Abs against IFN-β was significantly associated with critical, but not severe, disease relative to the controls (Table 1, Tables S2–3). Interestingly, Gyros did not appear to be able to detect auto-Abs against IFN-β, perhaps because of the biotinylation of the cytokine hiding the epitope recognized by the auto-Abs. As most (78.3%) of the patients with neutralizing auto-Abs against IFN-β did not have neutralizing auto-Abs against IFN-α2 or IFN-ω, this suggests that auto-Abs against IFN-β alone may also underlie life-threatening COVID-19 (Table 1).

### Neutralizing auto-Abs against type I IFNs in at least 20% of critical patients over 80 years of age

**Fig. 3.**
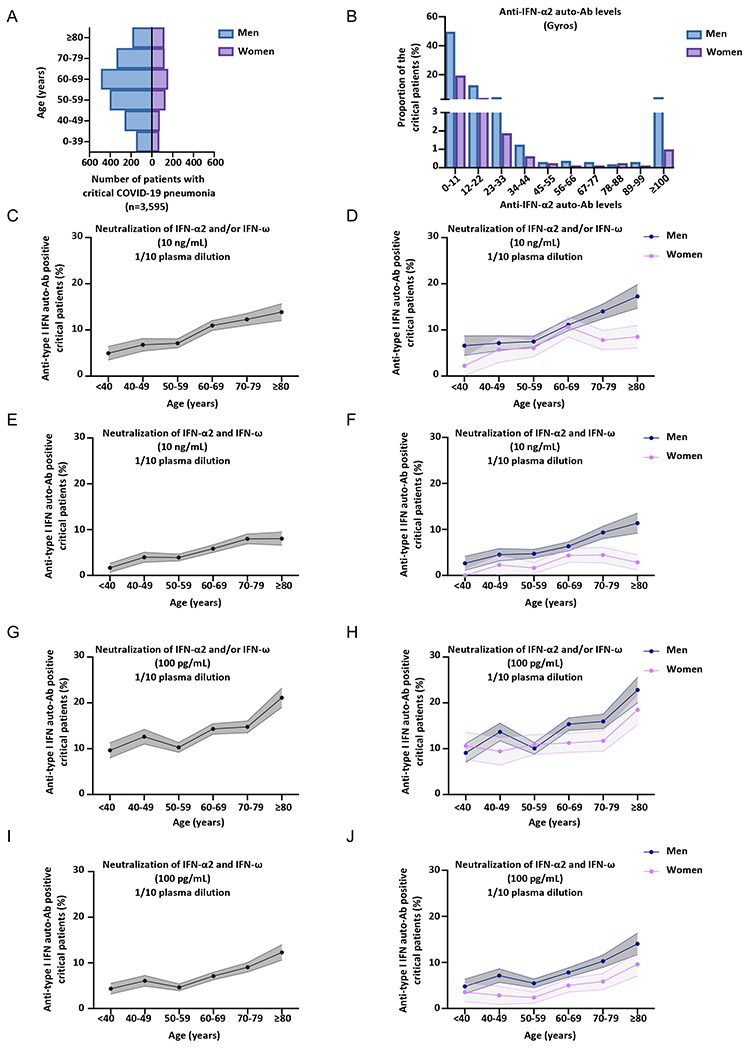
Higher prevalence of neutralizing auto-Abs against type I IFNs in elderly patients with critical COVID-19. **(A)** Bar plot of the age and sex distribution of the patients with life-threatening COVID-19 included in our expanded cohort (*N*=3,595). **(B)** Graph showing the anti-IFN-α2 auto-Ab levels, assessed by Gyros, in patients with life-threatening COVID-19. Men and women are shown separately. The upper section of the Y-axis starts at 3%. **(C-J)** Proportion by decade of patients with critical COVID-19, and positive for neutralizing auto-Abs (in plasma 1/10) against **(C)** IFN-α2 and/or IFN-ω, at 10ng/mL, for both sexes. **(D)** IFN-α2 and/or IFN-ω, at 10ng/mL, for men or women. **(E)** IFN-α2 and IFN-ω, at 10ng/mL, for both sexes. **(F)** IFN-α2 and IFN-ω, at 10ng/mL, for men or women. **(G)** IFN-α2 and/or IFN-ω, at 100pg/mL, for both sexes. **(H)** IFN-α2 and/or IFN-ω, at 100pg/mL, for men or women. **(I)** IFN-α2 and IFN-ω, at 100pg/mL, for both sexes. **(J)** IFN-α2 and IFN-ω, at 100pg/mL, for men or women.

We further assessed the percentage of critical COVID-19 patients positive for neutralizing auto-Abs per decade of life and by sex (Fig. 3A–J, S3A–W) (Tables S1–4). In our previous report, we found that critical COVID-19 patients with auto-Abs neutralizing IFN-α2 or IFN-ω at 10ng/mL were older (more than half the patients with auto-Abs were over the age of 65 years) and more likely to be male (95% of the antibody carriers were men) (9). These results have been confirmed by other groups, albeit with a smaller proportion of men (11–14, 35). In our expanded cohort of patients with critical COVID-19 pneumonia (*N*=3,595), the mean age was 61 years and 73% of the patients were men (Fig. 3A, Table S4). We confirmed that critical patients with auto-Abs neutralizing IFN-α and/or IFN-ω at 10ng/ml were significantly older than those not carrying auto-Abs (mean age [SD] 65.8 years [14.1] versus 61.6 years [15.5], Firth’s multivariable logistic regression, *P*=3x10^−6^) and more likely to be male (78.5% versus 71%, Firth’s multivariable logistic regression, *P*=0.003). The proportion of critical COVID-19 patients with auto-Abs neutralizing 10ng/mL IFN-α2 and/or IFN-ω increased continuously, with auto-Abs detected in 5% of patients under the age of 40 years, 6.8% of those between 40 and 49 years of age, 7.1% of those between 50 and 59 years of age, 10.7% of those between 60 and 69 years of age, 12.3% of those between 70 and 79 years, and almost 14% in those over 80 (Fig. 3C–F, S3B–I). In severe patients, the proportion of auto-Abs was much more stable with age (Fig. S3T–W, Firth’s multivariable logistic regression *P*=0.16) and sex (Firth’s multivariable logistic regression *P*=0.44). Similar results were obtained for critical COVID-19 patients with auto-Abs neutralizing 100pg/mL IFN-α2 and/or IFN-ω, but with even higher proportions (Fig. 3G–J, S3L–S) (Table S1). Indeed, the proportion of patients with auto-Abs ranged from 9.6% of patients below the age of 40 years, to more than 21% of those over 80 (Fig. 3G–J, S3L–S). In men, the proportion of critical COVID-19 patients carrying auto-Abs neutralizing 100pg/mL IFN-α2 and/or IFN-ω increased to up to 23% over 80 years of age. A very different pattern was seen for auto-Abs neutralizing 10ng/mL IFN-β, with a more stable proportion of auto-Abs carriers according to age (Fig. S3J, K, Firth’s multivariable logistic regression, *P*=0.68) (Table S3). Overall, the prevalence of auto-Abs neutralizing 10ng/mL and/or 100pg/mL IFN-α2 and/or IFN-ω increased sharply with age in critical patients. A striking enrichment in patients with neutralizing auto-Abs against IFN-α2 and/or IFN-ω was observed in the elderly, with more than 20% of patients, and 23% of men, over the age of 80 years with critical COVID-19 having neutralizing auto-Abs against these type I IFNs.

### Neutralizing auto-Abs against type I IFNs in at least 18% of deceased patients

**Fig. 4.**
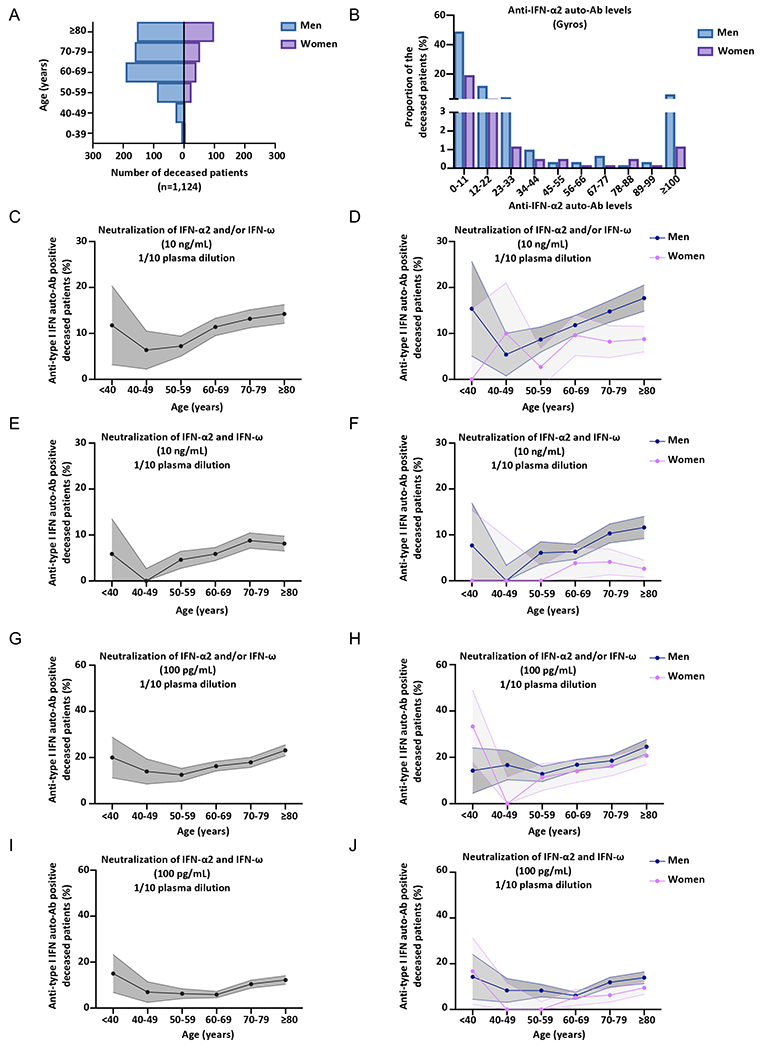
Higher prevalence of neutralizing auto-Abs against type I IFNs in patients who died of COVID-19. **(A)** Bar plot of the age and sex distribution of the patients who died of COVID-19 included in our cohort (*N*=1,124). **(B)** Graph showing the anti-IFN-α2 auto-Ab levels, assessed by Gyros, in patients who died of COVID-19. Men or women are shown separately. The upper section of the Y-axis starts at 3%. **(C-J)** Proportion by decade of patients who died of COVID-19, and positive for neutralizing auto-Abs (in plasma 1/10) against **(C)** IFN-α2 and/or IFN-ω, at 10ng/mL, for both sexes. **(D)** IFN-α2 and/or IFN-ω, at 10ng/mL, for men or women. **(E)** IFN-α2 and IFN-ω, at 10ng/mL, for both sexes. **(F)** IFN-α2 and IFN-ω, at 10ng/mL, for men or women. **(G)** IFN-α2 and/or IFN-ω, at 100pg/mL, for both sexes. **(H)** IFN-α2 and/or IFN-ω, at 100pg/mL, for men or women. **(I)** IFN-α2 and IFN-ω, at 100pg/mL, for both sexes. **(J)** IFN-α2 and IFN-ω, at 100pg/mL, for men or women.

The prevalence of auto-Abs against type I IFNs in patients dying from COVID-19 pneumonia is unknown. For the 3,595 patients with critical COVID-19, we analyzed data for the 1,124 who died. These patients were aged 20 days to 99 years (mean age: 71 years), 73% were male, and all had confirmed SARS-CoV-2 infection and critical COVID-19 pneumonia before death (Fig. 4A). In these patients, we analyzed the presence of neutralizing auto-Abs against type I IFNs at concentrations of 10ng/mL and 100pg/mL for IFN-α2 and IFN-ω, and at 10ng/mL for IFN-β (Fig. 4B–J, S4A–K). We found that 13.3% of the deceased patients carried auto-Abs neutralizing 10ng/mL IFN-α2 and/or IFN-ω (Fig. 4B–F, S4A–E). Strikingly, 18.5% carried auto-Abs neutralizing 100pg/mL of either or both cytokines (Fig. 4G–J, S4F–I). In addition, 0.9% had auto-Abs neutralizing IFN-β (Fig. S4J–K). An analysis of the prevalence of neutralizing auto-Abs against type I IFNs in these patients who died of COVID-19 by decade of age revealed a moderate increase with age for auto-Abs neutralizing 10ng/mL (Firth’s multivariable logistic regression *P*=0.03) or 100pg/mL (Firth’s multivariable logistic regression *P*=0.01) (Table S1–2). For a type I IFN concentration of 100pg/mL, the prevalence of auto-Abs neutralizing IFN-α2 and/or IFN-ω was 20% below the age of 40 years, 14% for individuals between 40 and 49 years old, 12.5% for those between 50 and 60 years old, 16.3% for those between 60 and 69 years old, 17.9% for those between 70 and 79 years old, and greater than 23% for those over the age of 80 years. Overall, at least 18% of patients dying from COVID-19 pneumonia have auto-Abs capable of neutralizing 100pg/mL type I IFNs in plasma 1/10.

### Auto-Abs capable of neutralizing IFN-α2 and/or IFN-ω at 10ng/mL in 0.53%, and at 100pg/mL in 2.3% of individuals from the general population

**Fig. 5.**
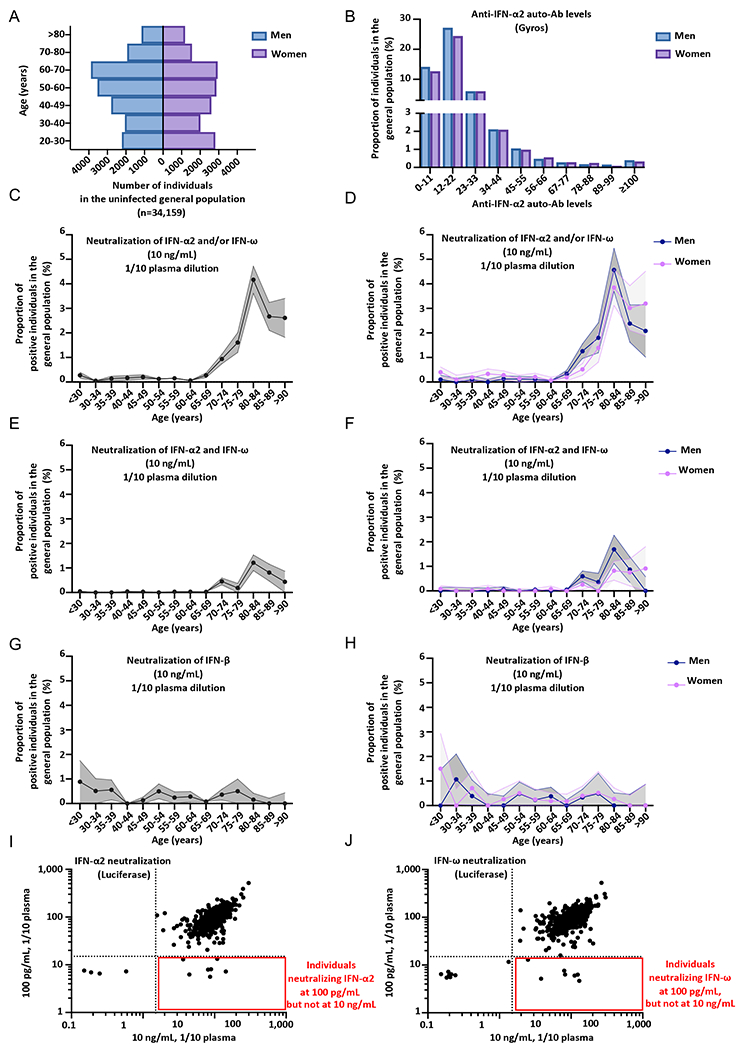
Neutralizing auto-Abs against IFN-α2 and/or IFN-ω at 10ng/mL are more prevalent in the elderly, in the general population. **(A)** Bar plot of the age and sex distribution of individuals from the general population (*N*=34,159). **(B)** Graph showing the IFN-α2 auto-Ab levels, assessed by Gyros, in individuals from the general population. Men or women are shown separately. The upper section of the Y-axis starts at 3%. **(C-H)** Proportion by 5 years of individuals from the general population, and positive for neutralizing auto-Abs (in plasma 1/10) against **(C)** IFN-α2 and/or IFN-ω, at 10ng/mL, for both sexes. **(D)** IFN-α2 and/or IFN-ω, at 10ng/mL, for men or women. **(E)** IFN-α2 and IFN-ω, at 10ng/mL, for both sexes. **(F)** IFN-α2 and IFN-ω, at 10ng/mL, for men or women. **(G)** IFN-β, at 10ng/mL, for both sexes. **(H)** IFN-β, at 10ng/mL, for men or women. **(I)** Plot showing luciferase induction after stimulation with 10ng/mL or 100pg/mL IFN-α2, in the presence of plasma from individuals from the general population. Dotted lines indicate neutralizing levels, defined as induction levels below 15% of the mean value for controls tested the same day. Individuals with antibodies neutralizing both 10ng/mL and 100pg/mL IFN-α2 are shown in the bottom left corner, whereas the individuals in the bottom right corner had antibodies capable of neutralizing only 100pg/mL IFN-α2. **(J)** Plot showing luciferase induction after stimulation with 10ng/mL or 100pg/mL IFN-ω, for individuals from the general population.

We previously tested a sample of 1,227 individuals aged 20 to 65 years from the general population collected in 2015-2017. This sample had an equal sex distribution, and we identified four individuals with auto-Abs against type I IFNs among the 1,227 tested (0.3%), suggesting that the auto-Abs pre-dated COVID-19 (9). These findings were replicated at the University of California San Francisco (UCSF) in a sample of 4,041 subjects aged 4 to 90 years (0.32%) (16). In the current study, we tested a much larger cohort of 34,159 individuals aged 20 to 100 years from the general population, with an equal distribution between the sexes (Fig. 5A). Samples were collected before 2018 for blood donors at the French blood bank (19,966 individuals), the 3C cohort (801) and in 2019 for participants in the French CONSTANCES cohort (8,850) and Cerba HealthCare (4,542). We performed serological tests for SARS-CoV-2 on the samples collected in 2019, and included only the individuals who had not been infected with SARS-CoV-2 in the sample. We used Gyros to screen this whole cohort for IgG auto-Abs against IFN-α2 and IFN-ω (Fig. 5B, S5A). We did not measure auto-Abs against IFN-β by Gyros. We found that only 0.05% and 4.2% had anti-IFN-α2 and/or anti-IFN-ω auto-Abs above the thresholds of 100 and 30, respectively (Fig. 5B, S5A). We then assessed the ability of these antibodies to neutralize 10ng/mL IFN-α2 or IFN-ω, for all individuals with a high or intermediate level of IgG auto-Abs against IFN-α2 or IFN-ω. We found 181 individuals with neutralizing auto-Abs, for whom 1/10 dilutions of plasma neutralized 10ng/mL IFN-α2 and/or IFN-ω, giving an overall prevalence of 0.53% (Fig. 5C–F, S5B–I) (Table S5–6), consistent with our two previous reports (9, 16). We may have slightly underestimated the number of positive individuals, as some may have had neutralizing auto-Abs at too low a titer for detection. Next, we assessed the prevalence of auto-Abs neutralizing 10ng/mL of IFN-β in 9,583 individuals, and found an overall prevalence of 0.26% (Fig. 5G–H) (Table S5–6). Finally, for a subset of 10,778 samples, we further assessed the ability of plasma/serum samples (diluted 1/10) to neutralize 100pg/mL IFN-α2 and/or IFN-ω in the luciferase assay (Fig. 5I–J, 6A–H). The prevalence of auto-Abs neutralizing 100pg/mL IFN-α2 and/or IFN-ω was 2.3% (Table S1).

### Sharp increase in the prevalence of auto-Abs against IFN-α2 and/or IFN-ω after the age of 70 years in the general population

**Fig. 6.**
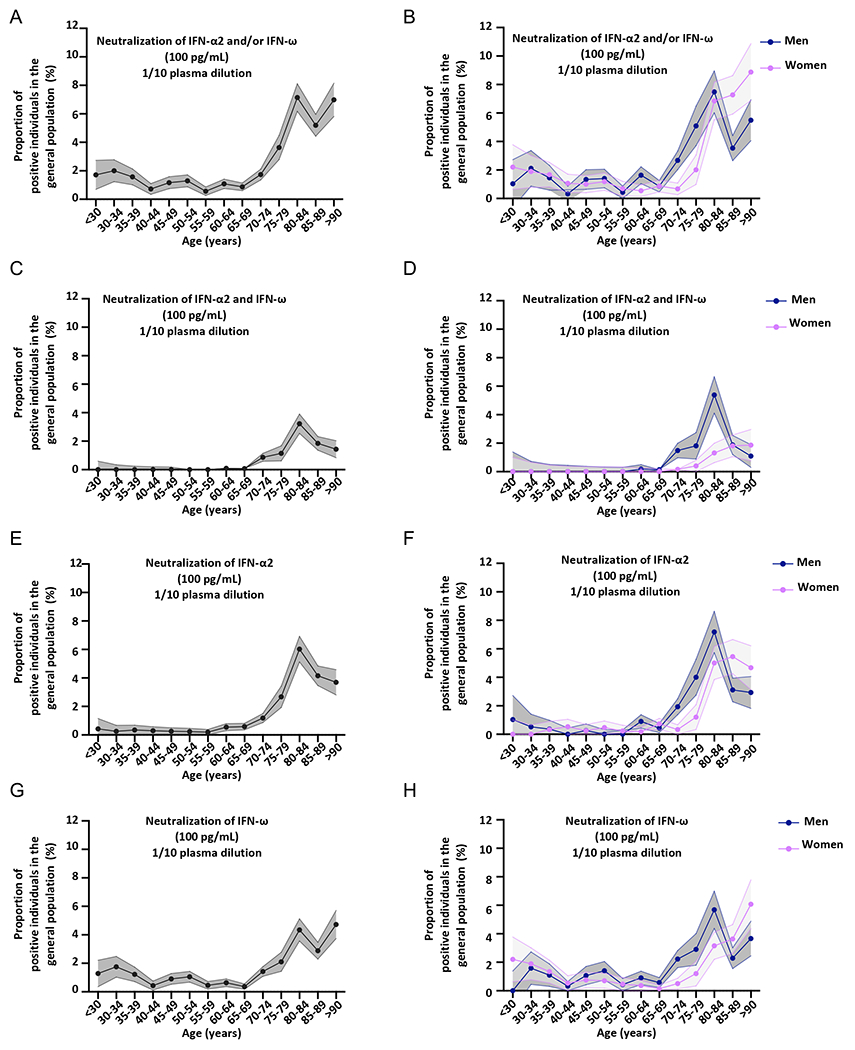
Neutralizing auto-Abs against IFN-α2 and/or IFN-ω at 100pg/mL are more prevalent in the elderly, in the general population. **(A-H)** Proportion, binned every 5 years, of individuals from the general population, and positive for neutralizing auto-Abs (in plasma 1/10) against **(A)** IFN-α2 and/or IFN-ω, at 100pg/mL, for both sexes. **(B)** IFN-α2 and/or IFN-ω, at 100pg/mL, for men or women. **(C)** IFN-α2 and IFN-ω, at 100pg/mL, for both sexes. **(D)** IFN-α2 and IFN-ω, at 100pg/mL, for men or women. **(E)** IFN-α2, at 100pg/mL, for both sexes. **(F)** IFN-α2, at 100pg/mL, for men or women. **(G)** IFN-ω, at 100pg/mL, for both sexes. **(H)** IFN-ω, at 100pg/mL, for men or women.

We then assessed the percentage of individuals from the general population positive for neutralizing auto-Abs per decade of life and by sex. Strikingly, we noted that the prevalence of auto-Abs neutralizing 10ng/mL type I IFN was more than 10 times higher in individuals over the age of 70 years than in those below this age (Firth’s multivariable logistic regression, *P*<10^−13^) (Fig. 5C–F, S5B–I) (Table S5–6). The prevalence of auto-Abs capable of neutralizing 10ng/mL IFN-α2 and/or IFN-ω was 0.17% in individuals below 70 years of age, 0.9% in individuals between 70 and 75 years of age, 1.6% between the ages of 75 and 80 years and more than 4% between the ages of 80 and 85 years. Intriguingly, after 85 years, the prevalence of these antibodies decreased to about 2.6%. These findings were replicated independently in two cohorts of 703 and 376 elderly individuals from Estonia and Japan, tested with Luciferase-based immunoprecipitation assay (LIPS) and ELISA assays, respectively (Fig. S5J, K). A strong increase in the prevalence of auto-Abs neutralizing 100pg/mL IFN-α2 and/or IFN-ω was observed with age (Fig. 6A–H, S6A–D), with the prevalence almost doubling with every five years from 65 to 85 years of age. Indeed, 0.87% of individuals between the ages of 65 and 70 years, 1.73% of those between 70 and 75 years, and 7.1% of those between 75 and 80 years were positive for auto-Abs. Interestingly, there was an overall decrease in the prevalence of auto-Abs after 85 years of age, especially in men. By contrast, the prevalence of auto-Abs neutralizing IFN-β did not vary significantly with age (Fig. 5G, H) (Table S4). We then assessed the risk, adjusted for age and sex, of having critical or severe disease, for subjects carrying auto-Abs against each individual IFN and the different possible combinations, relative to the general population. We also found that all auto-Abs were highly significant risk factors in comparisons of patients with critical or severe COVID-19 with the general population (Table 1 and Table S2). The strongest association was again that for auto-Abs neutralizing both IFN-α2 and IFN-ω at 10ng/mL (OR=30, *P*<1 x10^−13^), followed by those neutralizing IFN-α2 +/− IFN-ω at 10ng/mL (OR=20, *P*<10^−13^), and IFN-ω +/− IFN-α2 at 10ng/mL (OR =15, *P*<10^−13^) (Table 1). Auto-Abs neutralizing both IFN-α2 and IFN-ω at 100pg/mL were also highly significant risk factors (OR [95% CI]=12 [9-16], *P*<10^−13^) (Table 1). Overall, these findings indicate that there is a sharp increase in the prevalence of auto-Abs neutralizing type I IFNs with age in elderly uninfected individuals, with at least 4% of those over the age of 70 years positive for auto-Abs against IFN-α2 and/or IFN-ω, and that these auto-Abs pre-date COVID-19.

## Discussion

We report that at least 20% of patients over 80 years of age with life-threatening COVID-19 pneumonia carry circulating auto-Abs neutralizing 100pg/mL IFN-α2 and/or IFN-ω, and that such antibodies are present in more than 13.6% of patients of all ages with this condition. Some of these auto-Abs are not identified by immunoassays and are only detectable by a neutralization assay. In addition, at least 18% of deceased individuals in most age groups were found to have such auto-Abs. We also report that auto-Abs against IFN-β are found in about 1.3% and 0.9% of critical and deceased patients, most of whom do not have auto-Abs against IFN-α2 and/or IFN-ω. In all four patients tested for whom pre-COVID-19 samples were also available, the auto-Abs against IFN-α2 and/or IFN-ω were clearly present before SARS-CoV-2 infection, as in patients with APS-1 (9, 20), and in two other previously described patients (9). Importantly, auto-Abs capable of neutralizing high concentrations of type I IFNs have been found in patients without inborn errors of TLR3- or TLR7-dependent type I IFN immunity (7, 36), suggesting that both inborn errors and auto-Abs are independently causal of critical disease. It is also striking that inborn errors are more common in patients under the age of 60 years, whereas auto-Abs are more common in patients over the age of 70 years. We also report that the prevalence of auto-Abs neutralizing 10ng/mL (and 100pg/mL) type I IFNs, except for IFN-β**,** increases significantly with age in the general population, with 0.17% (1.1%) of individuals positive for these antibodies before the age of 70 years, and more than 1.4% (4.4%) positive after the age of 70 years, with a prevalence of 4.2% (7.1%) between the ages of 80 and 85 years.

These auto-Abs provide an explanation for the major increase in the risk of critical COVID-19 in the elderly. This increase with age is consistent with studies of various auto-Abs since the 1960s (39–43). These auto-Abs appear to have remained clinically silent in these individuals until SARS-CoV-2 infection. Our results also suggest that the neutralization of only one type I IFN (IFN-α2, IFN-ω, or IFN-β) can underlie life-threatening COVID-19 (Table 1, Tables S1–S3). Auto-Abs neutralizing 10ng/mL IFN-β have a frequency only about one tenth that of auto-Abs neutralizing the same concentrations of IFN-α2 and/or IFN-ω (Table 1, Table S3). We have shown that auto-Abs neutralizing 100pg/mL type I IFN in plasma diluted 1/10, corresponding to the neutralization of 1ng/mL IFN *in vivo*, can account for at least 18% of deaths and more than 20% of critical cases in the elderly >80 years of age. It is tempting to speculate that an even greater proportion of life-threatening COVID-19 cases are due to auto-Abs neutralizing lower, physiological concentrations of type I IFNs. *In vitro*, concentrations of type I IFN as low as 100pg/mL can impair SARS-CoV-2 replication in epithelial cells (Fig. 2A). Moreover, the levels of type I IFN detected in the blood of patients with acute and benign SARS-CoV-2 infections are in the range of 1 to 100pg/mL (32, 33).

Our findings have immediate clinical applications. First, it is quick and easy to test for auto-Abs against type I IFNs in patients infected with SARS-CoV-2. Screening for these antibodies is even possible in the general population before infection. The type I IFN-neutralizing activity of these antibodies is a better read-out than their mere detection, which can be falsely negative. Tests should be performed for auto-Abs against at least three individual IFNs: IFN-α2, IFN-ω, and IFN-β. Particular attention should be paid to elderly individuals, and patients with known autoimmune or genetic conditions associated with auto-Abs against type I IFNs (17–20, 22, 23, 26–29). Second, patients with auto-Abs against type I IFN should be vaccinated against COVID-19 as a priority. Third, live attenuated vaccines, including YFV-17D and vaccines using the YFV-17D backbone against SARS-CoV-2, should not be given to patients with auto-Abs (31, 44). Fourth, these patients appeared to be healthy before SARS-CoV-2 infection, but they should also be carefully followed for other viral illnesses, as exemplified by adverse reactions to YFV-17D (31). Fifth, in cases of SARS-CoV-2 infection in unvaccinated individuals with auto-Abs against type I IFNs, the patients should be hospitalized for prompt management. Early treatment with monoclonal antibodies (45, 46) can be administered in patients without symptoms of severe COVID-19 pneumonia, and IFN-β can be administered in the absence of both pneumonia and auto-Abs against IFN-β (37, 38). Rescue treatment by plasma exchange is another therapeutic option in patients who already have pneumonia (47).

Sixth, blood products, especially plasma, should be screened for anti-IFN auto-Abs and any products containing such antibodies should be excluded from donation (13). Plasma from donors convalescing from COVID-19 should be tested for such auto-Abs (13). Seventh, given the documented innocuity and potential efficacy of a single injection, early therapy with IFN-β may be considered for the contacts of contagious subjects or during the first week after infection, even in the absence of, or before the documentation of auto-Abs against type I IFNs, in elderly patients, who have a higher risk of critical pneumonia and auto-Abs against IFN-α2 and IFN-ω, but not IFN-β (48). Another possibility would be the administration of monoclonal antibodies that can neutralize SARS-CoV-2 (45, 46). Finally, it will be important to decipher the mechanism underlying the development of these auto-Abs, which may differ in patients over and under 65 years of age. Overall, our findings show that auto-Abs neutralizing concentrations of type I IFN lower than previously reported (9, 11–16), but still higher than physiological concentrations, are common in the elderly population. Their prevalence increases with age in the uninfected general population, reaching more than 4% of individuals after the age of 70 years. They underlie about 20% of cases of critical COVID-19 pneumonia in patients over the age of 80 years, and about 20% of total COVID-19 deaths. We previously reported that they can underlie severe adverse reactions to the yellow fever live attenuated virus (31). It is tempting to speculate that they may also underlie other severe viral diseases, especially in the elderly.

## Materials and methods

### Study design

We enrolled, from 38 countries across all continents, 3,595 patients with proven critical COVID-19, 623 with severe COVID-19 and 1,639 asymptomatic or paucisymptomatic individuals with proven COVID-19, and 34,159 healthy controls in this study. We collected plasma or serum samples for all these individuals. All subjects were recruited according to protocols approved by local institutional review boards (IRBs).

### COVID-19 classification

The severity of COVID-19 was assessed for each patient as follows (7, 9). “Critical COVID-19 pneumonia” was defined as pneumonia developing in patients with critical disease, whether pulmonary, with high-flow oxygen, mechanical ventilation (Continuous positive airway pressure, bilevel positive airway pressure, intubation), septic shock, or with damage to any other organ requiring admission to the intensive care unit. “Severe COVID-19” was defined as pneumonia developing in patients requiring low-flow oxygen (<6L/min). The controls were individuals infected with SARS-CoV-2 (as demonstrated by a positive PCR and/or serological test and/or displaying typical symptoms, such as anosmia/ageusia after exposure to a confirmed COVID-19 case) who remained asymptomatic or developed mild, self-healing, ambulatory disease with no evidence of pneumonia.

### Detection of anti-cytokine autoantibodies

#### Gyros

Cytokines, recombinant human (rh)IFN-α2 (Milteny Biotec, ref. number 130-108-984) or rhIFN-ω (Merck, ref. number SRP3061), were first biotinylated with EZ-Link Sulfo-NHS-LC-Biotin (Thermo Fisher Scientific, cat. number A39257), according to the manufacturer’s instructions, with a biotin-to-protein molar ratio of 1:12. The detection reagent contained a secondary antibody (Alexa Fluor 647 goat anti-human IgG (Thermo Fisher Scientific, ref. number A21445) diluted in Rexip F (Gyros Protein Technologies, ref. number P0004825; 1/500 dilution of the 2 mg/mL stock to yield a final concentration of 4 μg/mL). Buffer PBS-T 0.01% and Gyros Wash buffer (Gyros Protein Technologies, ref. number P0020087) were prepared according to the manufacturer’s instructions. Plasma or serum samples were then diluted 1/100 in PBS-T 0.01% and tested with the Bioaffy 1000 CD (Gyros Protein Technologies, ref. number P0004253), and the Gyrolab X-Pand (Gyros Protein Technologies, ref. number P0020520). Cleaning cycles were performed in 20% ethanol.

#### Multiplex particle-based assay

Serum/plasma samples were screened for autoantibodies (auto-Abs) against IFN-α2 and IFN-ω in a multiplex particle-based assay, in which magnetic beads with differential fluorescence were covalently coupled to recombinant human proteins (2.5 μg/reaction). Beads were combined and incubated with 1/100-diluted serum/plasma samples for 30 minutes. Each sample was tested once. The beads were then washed and incubated with PE-labeled goat anti-human IgG (1 μg/mL) for an additional 30 minutes. They were then washed again and used for a multiplex assay on a BioPlex X200 instrument.

#### Enzyme-linked immunosorbent assays (ELISA)

ELISA was performed as previously described. In brief, 96-well ELISA plates (MaxiSorp; Thermo Fisher Scientific) were coated by incubation overnight at 4°C with 2 μg/mL rhIFN-α2 (Milteny Biotec, ref. number 130-108-984), and rhIFN-ω (Merck, ref. number SRP3061). Plates were then washed (PBS 0.005% Tween), blocked by incubation with 5% nonfat milk powder in the same buffer, washed, and incubated with 1:50 dilutions of plasma from the patients or controls for 2 h at room temperature (or with specific mAbs as positive controls). Each sample was tested once. Plates were thoroughly washed. Horseradish peroxidase (HRP)–conjugated Fc-specific IgG fractions from polyclonal goat antiserum against human IgG, IgM or IgA (Nordic Immunological Laboratories) were added to a final concentration of 2 μg/mL. Plates were incubated for 1 h at room temperature and washed. Substrate was added and the optical density (OD) was measured. A similar protocol was used to test for antibodies against 12 subtypes of IFN-α, except that the plates were coated with cytokines from PBL Assay Science (catalog #11002-1), or IFN-β (Milteny Biotech, ref. number: 130-107-888).

### Functional evaluation of anti-cytokine autoantibodies

#### Luciferase reporter assays

The blocking activity of anti-IFN-α2 and anti-IFN-ω auto-Abs was determined with a reporter luciferase activity. Briefly, HEK293T cells were transfected with a plasmid containing the firefly luciferase gene under the control of the human *ISRE* promoter in the pGL4.45 backbone, and a plasmid constitutively expressing *Renilla* luciferase for normalization (pRL-SV40). Cells were transfected in the presence of the X-tremeGene9 transfection reagent (Sigma-Aldrich, ref. number 6365779001) for 24 hours. Cells in Dulbecco’s modified Eagle medium (DMEM, Thermo Fisher Scientific) supplemented with 2% fetal calf serum (FCS) and 10% healthy control or patient serum/plasma (after inactivation at 56°C, for 20 minutes) were either left unstimulated or were stimulated with IFN-α2 (Milteny Biotec, ref. number 130-108-984), IFN-ω (Merck, ref. number SRP3061), at 10ng/mL or 100pg/mL, or IFN-β (Milteny Biotech, ref. number: 130-107-888) at 10ng/mL, for 16 hours at 37°C. Each sample was tested once for each cytokine and dose. Finally, cells were lysed for 20 minutes at room temperature and luciferase levels were measured with the Dual-Luciferase® Reporter 1000 assay system (Promega, ref. number E1980), according to the manufacturer’s protocol. Luminescence intensity was measured with a VICTOR-X Multilabel Plate Reader (PerkinElmer Life Sciences, USA). Firefly luciferase activity values were normalized against *Renilla* luciferase activity values. These values were then normalized against the median induction level for non-neutralizing samples, and expressed as a percentage. Samples were considered neutralizing if luciferase induction, normalized against *Renilla* luciferase activity, was below 15% of the median values for controls tested the same day.

A similar protocol was used to test for auto-Abs against 12 subtypes of IFN-α, except that we used cytokines from PBL Assay Science (catalog #11002-1) at 1ng/mL for stimulation.

#### pSTAT1 induction in PBMC

The blocking activity of anti-IFN-α2 and anti-IFN-ω auto-Abs was determined by assessing STAT1 phosphorylation in healthy control cells following stimulation with the appropriate cytokines in the presence of 10% healthy control or patient serum/plasma. Surface-stained healthy control PBMCs (350,000/reaction) were cultured in serum-free RPMI medium with 10% healthy control or patient serum/plasma and were either left unstimulated or were stimulated with IFN-α2 or IFN-ω (10 ng/mL) for 15 minutes at 37°C. Each sample was tested once. Cells were fixed, permeabilized, and stained for intranuclear phospho-STAT1 (Y701). Cells were acquired on a BD LSRFortessa cytometer with gating on CD14^+^ monocytes and the data were analyzed with FlowJo software.

#### Luciferase-based immunoprecipitation assay (LIPS)

Levels of autoantibodies against IFN-α subtypes were measured in luciferase-based immunoprecipitation assay (LIPS), as previously described. IFNA1, IFNA2, IFNA8, and IFNA21 sequences were inserted into a modified pPK-CMV-F4 fusion vector (PromoCell GmbH, Germany), in which the firefly luciferase replaced the *NanoLuc* luciferase (Promega, USA). The resulting constructs were used to transfect HEK293 cells and the IFNA-luciferase fusion proteins were collected in the tissue culture supernatant. For autoantibody screening, we combined 2x10^6^ luminescence units (LU) of IFNA1, IFNA2, IFNA8 and IFNA21 in a single IP reaction mixture (pool 1), and IFNA4, IFNA5, IFNA6 and IFNA7 in another IP reaction mixture (pool 2). Serum samples were incubated with Protein G agarose beads (Exalpha Biologicals, USA) at room temperature for 1 h in a 96-well microfilter plate (Merck Millipore, Germany), and we then added 2x10^6^ luminescence units (LU) of antigen and incubated for another hour. Each sample was tested once. The plate was washed with a vacuum system and Nano-Glo® Luciferase Assay Reagent (Promega, USA) was added. Luminescence intensity was measured with a VICTOR X Multilabel Plate Reader (PerkinElmer Life Sciences, USA). The results are expressed in arbitrary units (AU), as a fold-difference relative to the mean of the negative control samples.

### IgG purification

We demonstrated that the IFN-α2 or IFN-ω neutralizing activity observed was due to auto-Abs and not another plasma factor, by depleting IgG from the plasma with a protein G buffer (Pierce™ Protein G IgG Binding Buffer, 21011) and column (NAb™ Protein G Spin Columns, 89953). All buffers were homemade: glycine 0.1 M pH=2.7, Tris 1.5 M pH = 8. Total plasma was loaded onto the column. Each sample was tested once. Purified IgG were then concentrated (Pierce™ Protein Concentrators PES, 50K MWCO, 88504). Without eluting the IgG, the flow-through fraction (IgG-depleted) was then collected and compared to total plasma in the luciferase neutralization assay.

### Statistical analysis

Odds ratios (OR) and P-values for the effect of auto-Abs neutralizing each type I IFN on critical or severe COVID-19, using asymptomatic/mild patients or the general population as controls and adjusted on age in years and sex, were estimated by means of Firth’s bias-corrected logistic regression (49, 50) as implemented in the “logistf” R package (https://rdrr.io/cran/logistf/). Effect of age (quantitative in years or binary +/− 65 years) and sex on the presence of neutralizing auto-Abs in each cohort (critical, severe, deceased and general population) was tested by multivariable Firth’s bias-corrected logistic regression. The standard error of the prevalence of neutralizing auto-Abs to each type I IFN per age groups and sex were estimated using the Agresti-Coull approximation (51).

### Schematic representation

Schematic representations (Fig. 1B) were created with BioRender.com.

### SARS-CoV-2 experiment

SARS-CoV-2 strain USA-WA1/2020 was obtained from BEI Resources and amplified in Caco-2 cells at 37°C. Viral titers were measured on Huh-7.5 hepatoma cells in a standard plaque assay. Caco-2 (*H. sapiens,* sex: male, colon epithelial) and Huh-7.5 cells (*H. sapiens,* sex: male, liver epithelial) were cultured in DMEM supplemented with 1% nonessential amino acids (NEAA) and 10% fetal bovine serum (FBS) at 37°C, under an atmosphere containing 5% CO_2_. Both cell lines have been tested negative for contamination with mycoplasma. SARS-CoV-2 experiments were performed as follows. Huh-7.5 cells were used to seed 96-well plates at a density of 7.5x10^3^ cells/well. The following day, plasma samples or a commercial anti-IFN-α2 antibody (catalog number 21100-1; R&D Systems) were diluted to 1% and incubated with 5 pM (~100 pg/mL) or 20 pM (~400 pg/mL) recombinant IFN-α2 (catalog number 11101-2; R&D systems) for 1 h at 37°C (dilutions: plasma samples = 1/100 and anti-IFN-α2 antibody = 1/1,000). Molar ratio was calculated according to the manufacturer’s datasheet and with http://molbiol.ru/eng/scripts/01_04.html. Following this incubation period, the cell culture medium was removed from the 96-well plates by aspiration and replaced with the plasma/anti-IFN-α2 antibody and IFN-α2 mixture. Each sample was tested once, in triplicate. The plates were incubated overnight and the plasma/anti-IFN-α2 antibody plus IFN-α2 mixture was removed by aspiration. The cells were washed once with PBS to remove potential anti-SARS-CoV-2-neutralizing antibodies and fresh medium was then added. Cells were then infected with SARS-CoV-2 by directly adding the virus to the wells. Cells infected at a MOI of 0.05 PFU/cell and incubated at 33°C for 48 hours. The cells were fixed with 7% formaldehyde, stained for SARS-CoV-2 with an anti-N antibody (catalog no. GTX135357; GeneTex), imaged and analyzed as previously described (9).

## Supplementary Material

Table S1

Supplemental Material

## Figures and Tables

**Table 1:** Risk of critical COVID-19 pneumonia for subjects carrying auto-Abs to specific sets of type I IFNs, when compared with that of asymptomatic/mild infection, adjusted on age and sex. #. Odds ratios (OR) and *P*-values were estimated by means of Firth’s bias-corrected logistic regression. The numbers and proportions of subjects with critical COVID-19 pneumonia (patients) and asymptomatic or mild infection (controls) are shown in Figures 1 to 3. Two combinations are not shown due to insufficient number of individuals: anti-IFN-β (10ng/mL) and anti-IFN-α2 (100pg/mL) auto-Abs only; anti-IFN-β (10ng/mL) and anti-IFN-ω (100pg/mL) auto-Abs only.

Anti-type I IFN auto-Ab positive (amount of type I IFN neutralized, in plasma diluted 1/10)	Proportion of critical patients with neutralizing auto-Abs	OR [95% CI]	*P*-value
anti-IFN-α2 and anti-IFN-ω auto-Abs (10 ng/mL)	5.6%	67 [4-1109]	7.8x10^−13^
anti-IFN-α2 and/or anti-IFN-ω auto-Abs (10 ng/mL)	9.8%	17 [7-45]	< 10^−13^
anti-IFN-α2 auto-Abs (10 ng/mL)	9%	45 [9-225]	< 10^−13^
anti-IFN-α2 auto-Abs only (10 ng/mL)	3.4%	21 [4-107]	1.8x10^−09^
anti-IFN-ω auto-Abs (10 ng/mL)	6.4%	13 [4-38]	1.4x10^−12^
anti-IFN-ω auto-Abs only (10 ng/mL)	0.8%	3 [0.9-10]	0.057
anti-IFN-α2 and anti-IFN-ω auto-Abs (100 pg/mL)	7.1%	54 [11-275]	< 10^−13^
anti-IFN-α2 and/or anti-IFN-ω auto-Abs (100 pg/mL)	13.6%	13 [8-21]	< 10^−13^
anti-IFN-α2 auto-Abs (100 pg/mL)	10%	23 [10-55]	< 10^−13^
anti-IFN-α2 auto-Abs only (100 pg/mL)	2.9%	10 [3-26]	2.8x10^−09^
anti-IFN-ω auto-Abs (100 pg/mL)	10.7%	13 [7-23]	< 10^−13^
anti-IFN-ω auto-Abs only (100 pg/mL)	3.6%	6 [3-12]	3.9x10^−10^
anti-IFN-β auto-Abs (10 ng/mL)	1.3%	8 [2-36]	1.7x10^−3^
anti-IFN-β auto-Abs only (10 ng/mL)	0.96%	5 [1-25]	0.043
anti-IFN-β auto-Abs (10ng/mL) and, anti-IFN-α2 and/or anti-IFN-ω auto-Abs (100 pg/mL)	0.34%	16 [0.5-497]	0.018
anti-IFN-β (10 ng/mL) and, anti-IFN-α2 and anti-IFN-ω auto-Abs (100 pg/mL)	0.28%	16 [0.5-502]	0.019

## Data Availability

All the data are available in the manuscript or in the supplementary materials. Plasma, cells, and genomic DNA are available from J.-L.C. under a material transfer agreement with The Rockefeller University or the Imagine Institute. Huh-7.5 cells are available on request from C.M.R. under a material transfer agreement with The Rockefeller University and Apath, LLC. The materials and reagents used are almost exclusively commercially available and nonproprietary. Materials derived from human samples may be made available on request, subject to any underlying restrictions concerning such samples.

## Consortium co-authors

### HGID Lab

Benedetta Bigio^1^, Soraya Boucherit^2,3^, Aliénor de la Chapelle^2^, Jie Chen^1^, Maya Chrabieh^2,3^, Boubacar Coulibaly^2,3^, Dana Liu^1^, Yelena Nemirowskaya^1^, Inés Marín Cruz^2^, Marie Materna^2,3^, Sophie Pelet^2^, Yoann Seeleuthner^2,3^, Chloé Thibault^2,3^, Zhiyong Liu^1^.

^1^St. Giles Laboratory of Human Genetics of Infectious Diseases, Rockefeller Branch, The Rockefeller University, New York, NY, USA. ^2^Laboratory of Human Genetics of Infectious Diseases, Necker Branch, INSERM U1163, Necker Hospital for Sick Children, Paris, France. ^3^University of Paris, Imagine Institute, Paris, France.

### COVID Clinicians

Jorge Abad^1^, Giulia Accordino^2^, Cristian Achille^3^, Sergio Aguilera-Albesa^4^, Aina Aguiló-Cucurull^5^, Alessandro AIUTI^6^, Esra Akyüz Özkan^7^, Ilad Alavi Darazam^8^, Jonathan Antonio Roblero Albisures^9^, Juan C Aldave^10^, Miquel Alfonso Ramos^11^, Taj Ali Khan^12^, Anna Aliberti^13^, Seyed Alireza Nadji^14^, Gulsum Alkan^15^, Suzan A. AlKhater^16^, Jerome Allardet-Servent^17^, Luis M Allende^18^, Rebeca ALONSO-ARIAS^19^, Mohammed S Alshahrani^20^, Laia Alsina^21^, Marie-Alexandra Alyanakian^22^, Blanca Amador Borrero^23^, Zahir Amoura^24^, Arnau Antolí^25^, Romain Arrestier^26^, Mélodie Aubart^27^, Teresa Auguet^28^, Iryna Avramenko^29^, Gökhan Aytekin^30^, Axelle Azot^31^, Seiamak Bahram^32^, Fanny Bajolle^33^, Fausto Baldanti^34^, Aurélie Baldolli^35^, Maite Ballester^36^, Hagit Baris Feldman^37^, Benoit Barrou^38^, Federica BARZAGH^6^, Sabrina Basso^39^, Gulsum Iclal BAYHAN^40^, Alexandre Belot^41^, Liliana BEZRODNIK^42^, Agurtzane Bilbao^43^, Geraldine Blanchard-Rohner^44^, Ignacio Blanco^45^, Adeline Blandinières^46^, Daniel Blázquez-Gamero^47^, Alexandre Bleibtreu^48^, Marketa Bloomfield^49^, Mireia Bolivar-Prados^50^, Anastasiia BONDARENKO^51^, Alessandro Borghesi^3^, Raphael Borie^52^, Elisabeth Botdhlo-Nevers^53^, Ahmed A Bousfiha^54^, Aurore Bousquet^55^, David Boutolleau^56^, Claire Bouvattier^57^, Oksana Boyarchuk^58^, Juliette Bravais^59^, M. Luisa Briones^60^, Marie-Eve Brunner^61^, Raffaele Bruno^62^, Maria Rita P Bueno^63^, Huda Bukhari^64^, Jacinta Bustamante^33^, Juan José Cáceres Agra^65^, Ruggero Capra^66^, Raphael Carapito^67^, Maria Carrabba^68^, Giorgio CASARI^6^, Carlos Casasnovas^69^, Marion Caseris^70^, Irene Cassaniti^34^, Martin Castelle^71^, Francesco Castelli^72^, Martín Castillo de Vera^73^, Mateus V Castro^63^, Emilie Catherinot^74^, Jale Bengi Celik^75^, Alessandro Ceschi^76^, Martin Chalumeau^77^, Bruno Charbit^78^, Matthew P. Cheng^79^, Père Clavé^50^, Bonaventura Clotet^80^, Anna Codina^81^, Yves Cohen^82^, Roger Colobran^83^, Cloé Comarmond^84^, Alain Combes^85^, Patrizia Comoli^39^, Angelo G Corsico^2^, Taner Coşkuner^86^, Aleksandar Cvetkovski^87^, Cyril Cyrus^88^, David Dalmau^89^, François Danion^90^, David Ross Darley^91^, Vincent Das^92^, Nicolas Dauby^93^, Stéphane Dauger^94^, Paul De Munter^95^, Loic de Pontual^96^, Amin Dehban^97^, Geoffroy Delplancq^98^, Alexandre Demoule^99^, Isabelle Desguerre^100^, Antonio Di Sabatino^101^, Jean-Luc Diehl^102^, Stephanie Dobbelaere^103^, Elena Domínguez-Garrido^104^, Clément Dubost^105^, Olov EKWALL^106^, Şefika Elmas Bozdemir^107^, Marwa H Elnagdy^108^, Melike Emiroglu^15^, Akifumi Endo^109^, Emine Hafize Erdeniz^110^, Selma Erol Aytekin^111^, Maria Pilar ETXART LASA^112^, Romain Euvrard^113^, Giovanna Fabio^68^, Laurence Faivre^114^, Antonin Falck^115^, Muriel Fartoukh^116^, Morgane Faure^117^, Miguel Fernandez Arquero^118^, Ricard Ferrer^119^, Jose Ferreres^120^, Carlos Flores^121^, Bruno Francois^122^, Victoria Fumadó^123^, Kitty S C Fung^124^, Francesca Fusco^125^, Alenka Gagro^126^, Blanca Garcia Solis^127^, Pascale Gaussem^128^, Zeynep GAYRETLI^129^, Juana Gil-Herrera^130^, Laurent Gilardin^131^, Audrey Giraud Gatineau^132^, Mònica Girona-Alarcón^133^, Karen Alejandra Cifuentes Godínez^134^, Jean-Christophe Goffard^135^, Nacho GONZALES^136^, Luis I Gonzalez-Granado^137^, Rafaela González-Montelongo^138^, Antoine Guerder^139^, Belgin Gülhan^140^, Victor Daniel Gumucio^141^, Leif Gunnar Hanitsch^142^, Jan Gunst^143^, Marta Gut^144^, Jérôme Hadjadj^145^, Filomeen Haerynck^146^, Rabih Halwani^147^, Lennart Hammarström^148^, Selda HANCERLI^149^, Tetyana Hariyan^150^, Nevin Hatipoglu^151^, Deniz Heppekcan^152^, Elisa Hernandez-Brito^153^, Po-ki Ho^154^, María Soledad Holanda-Peña^155^, Juan P Horcajada^156^, Sami Hraiech^157^, Linda Humbert^158^, Ivan F N Hung^159^, Alejandro D. Iglesias^160^, Antonio Íñigo-Campos^138^, Matthieu Jamme^161^, María Jesús Arranz^89^, Marie-Thérèse Jimeno^162^, Iolanda Jordan^133^, Saliha Kanık Yüksek^163^, Yalcin Burak Kara^164^, Aydın Karahan^165^, Adem KARBUZ^166^, Kadriye Kart Yasar^167^, Ozgur Kasapcopur^168^, Kenichi Kashimada^169^, Sevgi Keles^111^, Yasemin Kendir Demirkol^170^, Yasutoshi Kido^171^, Can KIZIL^172^, Ahmet Osman Kılıç^173^, Adam Klocperk^174^, Antonia Koutsoukou^175^, Zbigniew J. Król^176^, Hatem Ksouri^177^, Paul Kuentz^178^, Arthur M C Kwan^179^, Yat Wah M Kwan^180^, Janette S Y Kwok^181^, Jean-Christophe Lagier^182^, David S Y Lam^183^, Vicky Lampropoulou^184^, Fanny Lanternier^185^, Yu-Lung LAU^186^, Fleur Le Bourgeois^94^, Yee-Sin Leo^187^, Rafael Leon Lopez^188^, Daniel Leung^186^, Michael Levin^189^, Michael Levy^94^, Romain Lévy^33^, Zhi Li^78^, Daniele Lilleri^34^, Edson Jose Adrian Bolanos Lima^190^, Agnes Linglart^191^, Eduardo López-Collazo^192^, José M. Lorenzo-Salazar^138^, Céline Louapre^193^, Catherine Lubetzki^193^, Kwok-Cheung Lung^194^, Charles-Edouard Luyt^195^, David C Lye^196^, Cinthia MAGNONE^197^, Davood Mansouri^198^, Enrico Marchioni^199^, Carola Marioli^2^, Majid Marjani^200^, Laura MARQUES^201^, Jesus Marquez Pereira^202^, Andrea Martín-Nalda^203^, David Martínez Pueyo^204^, Javier Martinez-Picado^205^, Iciar Marzana^206^, Carmen Mata-Martínez^207^, Alexis Mathian^24^, Larissa RB Matos^63^, Gail V Matthews^208^, Julien Mayaux^209^, Raquel McLaughlin-Garcia^210^, Philippe Meersseman^211^, Jean-Louis Mège^212^, Armand Mekontso-Dessap^213^, Isabelle Melki^115^, Federica Meloni^2^, Jean-François Meritet^214^, Paolo Merlani^215^, Özge METIN AKCAN^216^, Isabelle Meyts^217^, Mehdi Mezidi^218^, Isabelle Migeotte^219^, Maude Millereux^220^, Matthieu Million^221^, Tristan Mirault^222^, Clotilde Mircher^223^, Mehdi Mirsaeidi^224^, Yoko Mizoguchi^225^, Bhavi P Modi^226^, Francesco Mojoli^13^, Elsa MONCOMBLE^227^, Abián Montesdeoca Melián^228^, Antonio Morales Martinez^229^, Francisco Morandeira^230^, Pierre-Emmanuel Morange^231^, Clémence Mordacq^158^, Guillaume Morelle^232^, Stéphane J Mouly^233^, Adrián Muñoz-Barrera^138^, Cyril Nafati^234^, Shintaro Nagashima^235^, Yu Nakagama^171^, Bénédicte Neven^236^, João Farela Neves^237^, Lisa FP Ng^238^, Yuk-Yung Ng^239^, hubert Nielly^105^, Yeray Novoa Medina^210^, Esmeralda Nuñez Cuadros^240^, J. Gonzalo Ocejo-Vinyals^241^, Keisuke Okamoto^109^, Mehdi Oualha^33^, Amani Ouedrani^22^, Tayfun Özçelik^242^, Aslinur Ozkaya-Parlakay^140^, Michele Pagani^13^, Qiang Pan-Hammarström^148^, Maria Papadaki^243^, Christophe Parizot^209^, Philippe Parola^244^, Tiffany Pascreau^245^, Stéphane Paul^246^, Estela Paz-Artal^247^, Sigifredo Pedraza^248^, Nancy Carolina González Pellecer^134^, Silvia Pellegrini^249^, Rebeca Pérez de Diego^127^, Xosé Luis Pérez-Fernández^141^, Aurélien Philippe^250^, Quentin Philippot^116^, Adrien Picod^251^, Marc Pineton de Chambrun^85^, Antonio Piralla^34^, Laura Planas-Serra^252^, Dominique Ploin^253^, Julien Poissy^254^, Géraldine Poncelet^70^, Garyphallia Poulakou^175^, Marie S Pouletty^255^, Persia Pourshahnazari^256^, Jia Li Qiu-Chen^257^, Paul Quentric^209^, Thomas Rambaud^258^, Didier Raoult^212^, Violette RAOULT^259^, Anne-Sophie Rebillat^223^, Claire Redin^260^, Léa Resmini^261^, Pilar Ricart^262^, Jean-Christophe Richard^263^, Raúl Rigo-Bonnin^264^, Nadia rivet^46^, Jacques G Rivière^265^, Gemma Rocamora-Blanch^25^, Mathieu P RODERO^266^, Carlos Rodrigo^267^, Luis Antonio Rodriguez^190^, Carlos Rodriguez-Gallego^268^, Agustí Rodriguez-Palmero^269^, Carolina Soledad Romero^270^, Anya Rothenbuhler^271^, Damien Roux^272^, Nikoletta Rovina^175^, Flore Rozenberg^273^, Yvon Ruch^90^, Montse Ruiz^274^, Maria Yolanda Ruiz del Prado^275^, Juan Carlos Ruiz-Rodriguez^119^, Joan Sabater-Riera^141^, Kai Saks^276^, Maria Salagianni^184^, Oliver Sanchez^277^, Adrián Sánchez-Montalvá^278^, Silvia Sánchez-Ramón^279^, Laire Schidlowski^280^, Agatha Schluter^252^, Julien Schmidt^281^, Matthieu Schmidt^282^, Catharina Schuetz^283^, Cyril E Schweitzer^284^, Francesco Scolari^285^, Anna Sediva^286^, Luis Seijo^287^, Analia Gisela Seminario^42^, Damien Sene^23^, Piseth Seng^221^, Sevtap Senoglu^167^, Mikko Seppänen^288^, Alex Serra Llovich^289^, Mohammad Shahrooei^97^, Anna Shcherbina^290^, Virginie Siguret^291^, Eleni Siouti^292^, David M Smadja^293^, Nikaia Smith^78^, Ali Sobh^294^, Xavier Solanich^25^, Jordi Solé-Violán^295^, Catherine Soler^296^, Pere Soler-Palacín^297^, Betül Sözeri^86^, Giulia Maria Stella^2^, Yuriy Stepanovskiy^298^, Annabelle Stoclin^299^, Fabio Taccone^219^, Yacine Tandjaoui-Lambiotte^300^, Jean-Luc Taupin^301^, Simon J Tavernier^302^, Loreto Vidaur Tello^112^, Benjamin Terrier^303^, Guillaume Thiery^304^, Christian Thorball^260^, Karolina THORN^305^, Caroline Thumerelle^158^, Imran Tipu^306^, Martin Tolstrup^307^, Gabriele Tomasoni^308^, Julie Toubiana^77^, Josep Trenado Alvarez^309^, Vasiliki TRIANTAFYLLIA^310^, Sophie TROUILLET-ASSANT^311^, Jesús Troya^312^, Owen T Y Tsang^313^, Liina Tserel^314^, Eugene Y K Tso^315^, Alessandra Tucci^316^, Şadiye Kübra Tüter Öz^15^, Matilde Valeria Ursini^125^, Takanori Utsumi^225^, Yurdagul Uzunhan^317^, Pierre Vabres^318^, Juan Valencia-Ramos^319^, Ana Maria Van Den Rym^127^, Isabelle Vandernoot^320^, Valentina Velez-Santamaria^321^, Silvia Patricia Zuniga Veliz^134^, Mateus C Vidigal^322^, Sébastien Viel^253^, Cédric Vilain^323^, Marie E Vilaire-Meunier^223^, Judit Villar-García^324^, Audrey Vincent^57^, Guillaume Vogt^325^, Guillaume Voiriot^326^, Alla Volokha^327^, Fanny Vuotto^158^, Els Wauters^328^, Joost Wauters^329^, Alan K L Wu^330^, Tak-Chiu Wu^331^, Aysun Yahşi^332^, Osman YESILBAS^333^, Mehmet Yildiz^168^, Barnaby E Young^187^, Ufuk Yükselmiş^334^, Mayana Zatz^63^, Marco Zecca^39^, Valentina Zuccaro^62^, Van Praet Jens^335^, Lambrecht Bart N.^336^, Van Braeckel Eva^336^, Bosteels Cédric^336^, Hoste Levi^337^, Hoste Eric^338^, Fré Bauters^336^, Jozefien De Clercq^336^, Heijmans Cathérine^339^, Slabbynck Hans^340^, Naesens Leslie3^41^, Benoit Florkin^342^, Cécile Boulanger^343^, Dimitri Vanderlinden^344^

^1^Germans Trias i Pujol University Hospital and Research Institute, Badalona, Barcelona, Spain. ^2^Respiratory Diseases Division, IRCCS Policlinico San Matteo Foundation, University of Pavia, Pavia, Italy. ^3^Neonatal Intensive Care Unit, Fondazione IRCCS Policlinico San Matteo, Pavia, Italy. ^4^Navarra Health Service Hospital, Pamplona, Spain. ^5^Jeffrey Modell Diagnostic and Research Center for Primary Immunodeficiencies, Barcelona, Catalonia, Spain, Immunology Division, Genetics Department, Vall d’Hebron University Hospital (HUVH), Vall d’Hebron Research Institute (VHIR), Vall d’Hebron Barcelona Hospital Campus, Universitat Autònoma de Barcelona (UAB), Barcelona, Catalonia, Spain. Catalonia, Barcelona, Spain. ^6^Immunohematology Unit, San Raffaele Hospital, Milan, Italy. ^7^Ondokuz Mayıs University Medical Faculty Pediatrics, Samsun, Turkey. ^8^Department of Infectious Diseases, Loghman Hakim Hospital, Shahid Beheshti University of Medical Sciences, Tehran, Iran. ^9^Hospital Regional de Huehuetenango, “Dr. Jorge Vides de Molina”, Guatemala. ^10^Hospital Nacional Edgardo Rebagliati Martins, Lima, Peru. ^11^Parc Sanitari Sant Joan de Déu, Sant Boi de Llobregat Spain. ^12^Khyber Medical University, Khyber Pakhtunkhwa, Pakistan. ^13^Anesthesia and Intensive Care, Rianimazione I, Fondazione IRCCS Policlinico San Matteo, Pavia, Italy. ^14^Virology Research Center, National institutes of Tuberculosis and Lung diseases, Shahid Beheshti University of Medical Sciences, Tehran, Iran. ^15^Department of Pediatrics, Division of Pediatric Infectious Diseases, Selcuk University Faculty of Medicine, Konya, Turkey. ^16^College of Medicine, Imam Abdulrahman Bin Faisal University, Dammam, Saudi Arabia; Department of Pediatrics, King Fahad Hospital of the University, Al-Khobar, Saudi Arabia. ^17^Intensive care unit, Hôpital Européen, Marseille, France. ^18^Immunology Department, Hospital 12 de Octubre, Research Institute imas12, Complutense University, Madrid, Spain. ^19^Immunology Department, Asturias Central University Hospital, Biosanitary Research Institute of the Principality of Asturias (ISPA), Oviedo, Spain. ^20^Emergency and Critical Care Medicine Departments, College of Medicine, Imam AbdulRahman Ben Faisal University, Dammam, Saudi Arabia. ^21^Clinical Immunology and Primary Immunodeficiencias Unit, Hospital Sant Joan de Déu, Institut de Recerca Sant Joan de Déu, Barcelona; Universitat de Barcelona, Barcelona, Spain. ^22^Department of Biological Immunology, Necker Hospital for Sick Children, APHP and INEM, Paris, France. ^23^Internal medicine department, Hôpital Lariboisière, APHP; Université de Paris, Paris, France. ^24^Internal medicine department, Pitié-Salpétrière Hospital, Paris, France. ^25^Department of Internal Medicine, Hospital Universitari de Bellvitge, IDIBELL, Barcelona, Spain. ^26^Service de Médecine Intensive Réanimation, Hôpitaux Universitaires Henri Mondor, AP-HP; Groupe de Recherche Clinique CARMAS, Faculté de Santé de Créteil, Université Paris Est Créteil, Créteil, France. ^27^INSERM U1163, University of Paris, Imagine Institute, Paris, France & Pediatric Neurology Department, Necker-Enfants malades Hospital, APHP, Paris, France. ^28^Hospital U. de Tarragona Joan XXIII. Universitat Rovira i Virgili (URV). IISPV, Tarragona, Spain. ^29^Department of Propedeutics of Pediatrics and Medical Genetics, Danylo Halytsky Lviv National Medical University, Lviv, Ukraine. ^30^Department of Immunology and Allergy, Konya City Hospital, Konya, Turkey. ^31^Private practice, Paris, France. ^32^INSERM U1109, University of Strasbourg, Strasbourg, France. ^33^Necker Hospital for Sick Children, AP-HP, Paris, France. ^34^Molecular Virology Unit, Microbiology and Virology Department, Fondazione IRCCS Policlinico San Matteo, Pavia, Italy. ^35^Department of Infectious Diseases, CHU de Caen, Caen, France. ^36^Consorcio Hospital General Universitario , Valencia, Spain. ^37^The Genetics Institute, Tel Aviv Sourasky Medical Center and Sackler Faculty of Medicine, Tel Aviv University, Tel Aviv, Israel. ^38^Dept Urology, Nephrology, Transplantation, APHP-SU, Sorbonne Université, INSERM U 1082, Paris, France. ^39^Cell Factory and Pediatric Hematology-Oncology, Fondazione IRCCS Policlinico San Matteo, Pavia, Italy. ^40^Yildirim Beyazit University, Faculty of Medicine, Ankara City Hospital, Children’s Hospital, Ankara, Turkey. ^41^University of Lyon, CIRI, INSERM U1111, National referee centre RAISE, Pediatric Rheumatology, HFME, Hospices Civils de Lyon, Lyon, France. ^42^Center for Clinical Immunology, CABA, Buenos Aires, Argentina. ^43^Cruces University Hospital, Bizkaia, Spain. ^44^Paediatric Immunology and Vaccinology Unit, Geneva University Hospitals and Faculty of Medicine, Geneva, Switzerland. ^45^University Hospital and Research Institute “Germans Trias i Pujol”, Badalona, Spain. ^46^Hematology, Georges Pompidou Hospital, APHP, Paris, France. ^47^Pediatric Infectious Diseases Unit, Instituto de Investigación Hospital 12 de Octubre (imas12), Hospital Universitario 12 de Octubre, Universidad Complutense, Madrid, Spain. ^48^Infectious disease Unit, Pitié-Salpêtrière Hospital, AP-AP, Paris, France. ^49^Department of Pediatrics, Thomayer’s Hospital, 1st Faculty of Medicine, Charles University, Prague, Czech Republic; Department of Immunology, Motol University Hospital, 2nd Faculty of Medicine, Charles University, Prague, Czech Republic. ^50^Centro de Investigación Biomédica en Red de Enfermedades Hepàticas y Digestivas (Ciberehd). Hospital de Mataró, Consorci Sanitari del Maresme, Mataró, Spain. ^51^Shupyk National Healthcare University of Ukraine, Kyiv, Ukraine. ^52^Service de Pneumologie, Hopital Bichat, APHP, Paris, France. ^53^Department of infectious diseases, CIC1408, GIMAP CIRI INSERM U1111, University Hospital of Saint-Etienne, Saint-Etienne, France. ^54^Clinical immunology unit, pediatric infectious disease departement, Faculty of Medicine and Pharmacy, Averroes University Hospital. LICIA Laboratoire d’immunologie clinique, d’inflammation et d’allergie, Hassann Ii University., Casablanca, Morocco. ^55^Bégin Military Hospital, St Mandé, France. ^56^Sorbonne Université, INSERM, Institut Pierre Louis d’Epidémiologie et de Santé Publique (iPLESP), AP-HP, Hôpital Pitié Salpêtrière, Service de Virologie, Paris, France. ^57^Endocrinology unit, APHP Hôpitaux Universitaires Paris-Sud, Le Kremlin-Bicêtre, France. ^58^Department of Children’s Diseases and Pediatric Surgery, I.Horbachevsky Ternopil National Medical University, Ternopil, Ukraine. ^59^Pneumology Unit, Tenon Hospital, AP-HP, Paris, France. ^60^Department of Respiratory Diseases, Hospital Clínico y Universitario de Valencia, Valencia, Spain. ^61^Intensive care unit, Réseau Hospitalier Neuchâtelois, Neuchâtel, Switzerland. ^62^Infectious Diseases Unit, Fondazione IRCCS Policlinico San Matteo, Pavia, Italy. ^63^Human Genome and stem-cell research center-University of São Paulo, São Paulo, Brazil. ^64^Department of Internal Medicine, College of Medicine, Imam Abdulrahman Bin Faisal University, Dammam, Saudi Arabia. ^65^Hospital Insular, Las Palmas de Gran Canaria, Spain. ^66^MS Center, Spedali Civili, Brescia, Italy. ^67^Laboratoire d’ImmunoRhumatologie Moléculaire, plateforme GENOMAX, INSERM UMR_S 1109, Faculté de Médecine, ITI TRANSPLANTEX NG, Université de Strasbourg, Strasbourg, France. ^68^Fondazione IRCCS Ca’ Granda Ospedale Maggiore Policlinico, Milan, Italy. ^69^Neuromuscular Unit. Neurology Department. Hospital Universitari de Bellvitge - IDIBELL and CIBERER, Barcelona, Spain. ^70^Hopital Robert Debré, Paris, France. ^71^Pediatric Immuno-hematology Unit, Necker Enfants Malades Hospital, AP-HP, Paris, France. ^72^Department of Infectious and Tropical Diseases, University of Brescia, ASST Spedali Civili di Brescia, Brescia, Italy. ^73^Doctoral Health Care Center, Canarian Health System, Las Palmas de Gran Canaria, Spain. ^74^Hôpital Foch, Suresnes, France. ^75^Selcuk University Faculty of Medicine, Department of Anesthesiology and Reanimation, Intensive Care Medicine Unit, Konya, Turkey. ^76^Division of Clinical Pharmacology and Toxicology, Institute of Pharmacological Sciences of Southern Switzerland, Ente Ospedaliero Cantonale & Faculty of Biomedical Sciences, Università della Svizzera italiana, Lugano, Switzerland. ^77^Necker Hospital for Sick Children, Paris University, AP-HP, Paris, France. ^78^Pasteur Institute, Paris, France. ^79^McGill University Health Centre, Montreal, Canada. ^80^University Hospital and Research Institute “Germans Trias i Pujol”, IrsiCaixa AIDS Research Institute, UVic-UCC, Badalona, Spain. ^81^Clinical Biochemistry, Pathology, Paediatric Neurology and Molecular Medicine Departments and Biobank, Institut de Recerca Sant Joan de Déu and CIBERER-ISCIII, Esplugues, Spain. ^82^AP-HP, Avicenne Hospital, Intensive Care Unit, Bobigny, France; University Sorbonne Paris Nord, Bobigny, France; INSERM, U942, F-75010, Paris, France. ^83^Hospital Universitari Vall d’Hebron, Barcelona, Spain. ^84^Pitié-Salpêtrière Hospital, Paris, France. ^85^Service de médecine Intensive Réanimation, Groupe Hospitalier Pitié-Salpêtrière, Sorbonne Université, France. ^86^Umraniye Training and Research Hospital, Istanbul, Turkey. ^87^Faculty of Medical Sciences at University “Goce Delcev”, Shtip, North Macedonia. ^88^Department of Biochemistry, College of Medicine, Imam Abdulrahman Bin Faisal University, Dammam, Saudi Arabia. ^89^Fundació Docencia i Recerca Mutua Terrassa, Barcelona, Spain. ^90^Maladies Infectieuses et Tropicales, Nouvel Hôpital Civil, CHU Strasbourg, Strasbourg, France. ^91^UNSW Medicine, St Vincent’s Clinical School; Department of Thoracic Medicine, St Vincent’s Hospital Darlinghurst, Sidney, Australia. ^92^Intensive Care unit, Montreuil hospital, Montreuil, France. ^93^CHU Saint-Pierre, Université Libre de Bruxelles (ULB), Brussels, Belgium. ^94^Pediatric Intensive Care Unit, Robert-Debré University Hospital, APHP, Paris, France. ^95^General Internal Medicine, University Hospitals Leuven, Belgium. ^96^Hôpital Jean Verdier, APHP, Bondy, France. ^97^Specialized Immunology Laboratory of Dr. Shahrooei, Sina Medical Complex, Ahvaz, Iran. ^98^Centre de génétique humaine, CHU Besançon, Besançon, France. ^99^Sorbonne Université médecine and APHP Sorbonne université site Pitié-Salpêtrière, Paris, France. ^100^Pediatric Neurology Department, Necker-Enfants malades hospital, APHP, Paris, France. ^101^Department of Internal Medicine, Fondazione IRCCS Policlinico San Matteo, University of Pavia, Pavia, Italy. ^102^Intensive Care unit, Georges Pompidou Hospital, APHP, Paris, France. ^103^Department of Pneumology, AZ Delta, Roeselare, Belgium. ^104^Molecular Diagnostic Unit, Fundación Rioja Salud, Logroño, La Rioja, Spain. ^105^Bégin military Hospital, Saint Mandé, France. ^106^Department of Pediatrics, Institute of Clinical Sciences, The Sahlgrenska Academy, University of Gothenburg, Gothenburg, Sweden; Department of Rheumatology and Inflammation Research, Institute of Medicine, The Sahlgrenska Academy, University of Gothenburg, Gothenburg, Sweden. ^107^Bursa City Hospital, Bursa, Turkey. ^108^Department of Medical Biochemistry and Molecular Biology, Faculty of Medicine, Mansoura University, Mansoura, Egypt. ^109^Tokyo Medical and Dental University, Tokyo, Japan. ^110^Ondokuz Mayıs University Faculty of Medicine, Samsun, Turkey. ^111^Necmettin Erbakan University, Meram Medical Faculty, Division of Pediatric Allergy and Immunology, Konya, Turkey. ^112^University Donostia Hospital, Gipuzkoa, Spain. ^113^Internal Medicine, University Hospital Edouard Herriot, Hospices Civils de Lyon, Lyon, France. ^114^Centre de Génétique, CHU Dijon, Dijon, France. ^115^Robert Debré Hospital, Paris, France. ^116^APHP Tenon Hospital, Paris, France. ^117^Sorbonne Universités, UPMC University of Paris, Paris, France. ^118^Department of Clinical Immunology, Hospital Clínico San Carlos, Madrid, Spain. ^119^Intensive Care Department, Vall d’Hebron University Hospital (HUVH), Vall d’Hebron Barcelona Hospital Campus, Barcelona, Catalonia, Spain, Shock, Organ Dysfunction and Resuscitation Research Group. Vall d’Hebron Research Institute (VHIR), Vall d’Hebron Barcelona Hospital Campus, Barcelona, Catalonia, Spain. ^120^Intensive Care Unit, Hospital Clínico y Universitario de Valencia, Valencia, Spain. ^121^Genomics Division, Instituto Tecnológico y de Energías Renovables (ITER), Santa Cruz de Tenerife, Spain; CIBER de Enfermedades Respiratorias, Instituto de Salud Carlos III, Madrid, Spain; Research Unit, Hospital Universitario N.S. de Candelaria, Santa Cruz de Tenerife, Spain; Instituto de Tecnologías Biomédicas (ITB), Universidad de La Laguna, San Cristóbal de La Laguna, Spain, Santa Cruz de Tenerife, Spain. ^122^CHU Limoges and INSERM CIC 1435 & UMR 1092, Limoges, France. ^123^Infectious Diseases Unit, Department of Pediatrics, Hospital Sant Joan de Déu, Barcelona, Spain; Institut de Recerca Sant Joan de Déu, Spain; Universitat de Barcelona (UB), Barcelona, Spain. ^124^Department of Pathology, United Christian Hospital, Hong Kong. ^125^Institute of Genetics and Biophysics ‘Adriano Buzzati-Traverso’, IGB-CNR, Naples, Italy. ^126^Department of Pediatrics, Children’s Hospital Zagreb, University of Zagreb School of Medicine, Zagreb, Josip Juraj Strossmayer University of Osijek, Medical Faculty Osijek, Osijek, Croatia. ^127^Laboratory of Immunogenetics of Human Diseases, IdiPAZ Institute for Health Research, La Paz Hospital, Madrid, Spain. ^128^Hematology, APHP, Hopital Européen Georges Pompidou and INSERM UMR-S1140, Paris, France. ^129^Faculty of Medicine, Department of Pediatrics, Division of Pediatric Infectious Diseases, Karadeniz Technical University, Trabzon, Turkey. ^130^Division of Immunology, Hospital General Universitario and Instituto de Investigación Sanitaria “Gregorio Marañón”, Madrid, Spain. ^131^Bégin military Hospital, Bégin, France. ^132^Aix Marseille Univ, IRD, AP-HM, SSA, VITROME, IHU Méditerranée Infection, Marseille, France, French Armed Forces Center for Epidemiology and Public Health (CESPA), Marseille, France. ^133^Pediatric Intensive Care Unit, Hospital Sant Joan de Déu, Barcelona, Spain. ^134^Guatemala. ^135^Department of Internal Medicine, Hôpital Erasme, Université Libre de Bruxelles, Brussels, Belgium. ^136^Immunodeficiencies Unit, Research Institute Hospital, madrid, Spain. ^137^Primary Immunodeficiencies Unit, Pediatrics, University Hospital 12 octubre, Madrid, Spain; School of Medicine Complutense University of Madrid, Madrid, Spain. ^138^Genomics Division, Instituto Tecnológico y de Energías Renovables (ITER), Santa Cruz de Tenerife, Spain. ^139^Assistance Publique Hôpitaux de Paris, Paris, France. ^140^Ankara City Hospital, Ankara, Turkey. ^141^Department of Intensive Care, Hospital Universitari de Bellvitge, IDIBELL, Barcelona, Spain. ^142^Immunodeficiency Outpatient Clinic, Institute for Medical Immunology, FOCIS Center of Excellence, Charité Universitätsmedizin Berlin, Germany. ^143^Surgical Intensive Care Unit, University Hospitals Leuven, Leuven, Belgium. ^144^CNAG-CRG, Barcelona Institute of Science and Technology, Barcelona, Spain. ^145^Department of Internal Medicine, National Reference Center for Rare Systemic Autoimmune Diseases, AP-HP, APHP-CUP, Hôpital Cochin, Paris, France. ^146^Department of Paediatric Immunology and Pulmonology, Center for Primary Immunodeficiency Ghent, Jeffrey Modell Diagnosis and Research Center, PID research lab, Ghent University Hospital, Ghent, Belgium. ^147^Sharjah Institute of Medical Research, College of Medicine, University of Sharjah, Sharjah, UAE, Sharjah, UAE. ^148^Department of Biosciences and Nutrition, SE14183, Huddinge, Karolinska Institutet, Stockholm, Sweden. ^149^Department of Pediatrics (Infectious Diseases), Istanbul Faculty of Medicine, Istanbul University, Istanbul, Turkey. ^150^I. Horbachevsky Ternopil National Medical University, Ternopil, Ukraine. ^151^Pediatric Infectious Diseases Unit, Bakirkoy Dr. Sadi Konuk Training and Research Hospital, University of Health Sciences, Istanbul, Turkey. ^152^Health Sciences University, Darıca Farabi Education and Research Hospital, Kocaeli, Turkey. ^153^Department of Immunology , Hospital Universitario de Gran Canaria Dr. Negrín , Canarian Health System, Las Palmas de Gran Canaria, Spain. ^154^Department of Paediatrics, Queen Elizabeth Hospital, Hong Kong. ^155^IntensivenCare Unit. Marqués de Valdecilla Hospital, Santander, Spain. ^156^Hospital del Mar, Institut Hospital del Mar d’Investigacions Mèdiques (IMIM), UAB, UPF, Barcelona. ^157^Intensive care unit, APHM, Marseille, France. ^158^ CHU Lille, unité de pneumologie et allergologie pédiatriques, Lille, France. ^159^Department of Medicine, The University of Hong Kong, Hong Kong. ^160^Department of Pediatrics, Columbia University , New York, NY, USA. ^161^Centre hospitalier intercommunal Poissy Saint Germain en Laye, Poissy, France. ^162^IHU Méditerranée Infection, Service de l’Information Médicale, Hôpital de la Timone, Marseille, France. ^163^Health Science University Ankara City Hospital, Ankara, Turkey. ^164^School of Medicine, General Surgery Department Fevzi Çakmak Mah, Marmara University, Istanbul, Turkey. ^165^Mersin City Education and Research Hospital, Mersin, Turkey. ^166^Division of Pediatric Infectious Diseases, Prof. Dr. Cemil Tascıoglu City Hospital, Istanbul, Turkey. ^167^Departments of Infectious Diseases and Clinical Microbiology, Bakirkoy Dr. Sadi Konuk Training and Research Hospital, University of Health Sciences, Istanbul, Turkey. ^168^Department of Pediatric Rheumatology, Istanbul University-Cerrahpasa, Istanbul, Turkey. ^169^Department of Pediatrics, Tokyo Medical and Dental University, Tokyo, Japan. ^170^Health Sciences University, Umraniye Education and Research Hospital, Istanbul, Turkey. ^171^Department of Parasitology and Research Center for Infectious Disease Sciences, Graduate School of Medicine, Osaka City University, Osaka, Japan. ^172^Pediatric Infectious Diseases Unit of Osman Gazi University Medical School in Eskişehir, Turkey. ^173^Meram Medical Faculty, Necmettin Erbakan University, Konya, Turkey. ^174^Department of Immunology, 2nd Faculty of Medicine, Charles University and University Hospital in Motol, Prague, Czech Republic. ^175^ICU, 1st Department of Respiratory Medicine, National and Kapodistrian University of Athens, Medical School, ‘Sotiria’ General Hospital of Chest Diseases, Athens, Greece. ^176^Central Clinical Hospital of the Ministry of Interior and Administration, Warsaw, Poland. ^177^Clinique des soins intensifs, HFR Fribourg, Fribourg, Switzerland. ^178^Oncobiologie Génétique Bioinformatique, PC Bio, CHU Besançon, Besançon, France. ^179^Department of Intensive Care, Tuen Mun Hospital, Hong Kong. ^180^Paediatric Infectious Disease Unit, Hospital Authority Infectious Disease Center, Princess Margaret Hospital, Hong Kong (Special Administrative Region), China. ^181^Department of Pathology, Queen Mary Hospital, Hong Kong. ^182^Aix Marseille Univ, IRD, MEPHI, IHU Méditerranée Infection, Marseille, France. ^183^Department of Paediatrics, Tuen Mun Hospital, Hong Kong. ^184^Biomedical Research Foundation of the Academy of Athens, Athens, Greece. ^185^Necker hospital, Paris, France. ^186^Department of Paediatrics and Adolescent Medicine, The University of Hong Kong, Hong Kong, China. ^187^National Centre for Infectious Diseases, Singapore. ^188^Hospital Universitario Reina Sofía, Cordoba, Spain. ^189^Imperial College, London, England. ^190^Hospital General San Juan de Dios, Ciudad de Guatemala, Guatemala. ^191^Endocrinology and diabetes for children, AP-HP, Bicêtre Paris-Saclay hospital, Le Kremlin-Bicêtre, France. ^192^Innate Immunity group, IdiPAZ Institute for Health Research, La Paz Hospital, Madrid, Spain. ^193^Neurology unit, APHP Pitié-Salpêtrière Hospital, Paris University, Paris, France. ^194^Department of Medicine, Pamela Youde Nethersole Eastern Hospital, Hong Kong. ^195^Intensive care unit, APHP Pitié-Salpêtrière Hospital, Paris University, Paris, France. ^196^National Centre for Infectious Diseases; Tan Tock Seng Hospital; Yong Loo Lin School of Medicine; Lee Kong Chian School of Medicine, Singapore. ^197^Hospital de Niños Dr Ricardo Gutierrez, Buenos Aires, Argentina. ^198^Department of Clinical Immunology and Infectious Diseases, National Research Institute of Tuberculosis and Lung Diseases, Shahid Beheshti University of Medical Sciences, Tehran, Iran. ^199^Neurooncology and Neuroinflammation Unit, IRCCS Mondino Foundation, Pavia, Italy. ^200^Clinical Tuberculosis and Epidemiology Research Center, National Research Institute of Tuberculosis and Lung Diseases (NRITLD), Shahid Beheshti University of Medical Sciences, Tehran, Iran. ^201^Coordenadora da Unidade de Infeciologia e Imunodeficiências do Serviço de Pediatria, Centro Materno-Infantil do Norte, Porto, Portugal. ^202^Hospital Sant Joan de Déu and University of Barcelona, Barcelona, Spain. ^203^Pediatric Infectious Diseases and Immunodeficiencies Unit, Hospital Universitari Vall d’Hebron, Vall d’Hebron Research Institute, Vall d’Hebron Barcelona Hospital Campus, Universitat Autònoma de Barcelona (UAB), Barcelona, Catalonia, Spain. ^204^Hospital Universitari Mutua de Terrassa, Universitat de Barcelona, Barcelona, Spain. ^205^IrsiCaixa AIDS Research Institute, ICREA, UVic-UCC, Research Institute “Germans Trias i Pujol”, Badalona, Spain. ^206^Department of Laboratory, Cruces University Hospital, Barakaldo, Bizkaia, Spain, Bizkaia, Spain. ^207^Intensive Care Unit, Hospital General Universitario “Gregorio Marañón”, Madrid, Spain. ^208^University of New South Wales, Australia. ^209^APHP Pitié-Salpêtrière Hospital, Paris, France. ^210^Department of Pediatrics, Complejo Hospitalario Universitario Insular-Materno Infantil, Canarian Health System, Las Palmas de Gran Canaria, Spain. ^211^Medical Intensive Care Unit, University Hospitals Leuven, Leuven, Belgium. ^212^Aix-Marseille University, APHM, Marseille, France. ^213^Service de Médecine Intensive Réanimation, Hôpitaux Universitaires Henri Mondor, Assistance Publique - Hôpitaux de Paris (AP-HP). Groupe de Recherche Clinique CARMAS, Faculté de Santé de Créteil, Université Paris Est Créteil, France. ^214^APHP Cohin Hospital, Paris, France. ^215^Department of Critical Care Medicine, Ente Ospedaliero Cantonale, Bellinzona, Switzerland. ^216^Necmettin Erbakan University, Meram Medical Faculty, Division of Pediatric Infectious Diseases, Konya, Turkey. ^217^Department of Pediatrics, University Hospitals Leuven; KU Leuven, Department of Microbiology, Immunology and Transplantation; Laboratory for Inborn Errors of Immunity, KU Leuven, Leuven, Belgium. ^218^Hospices Civils de Lyon, Hôpital de la Croix-Rousse, Lyon, France. ^219^Hôpital Erasme, Brussels, Belgium. ^220^Centre hospitalier de Gonesse, Gonesse, France. ^221^Aix Marseille Univ, IRD, AP-HM, MEPHI, IHU Méditerranée Infection, Marseille, France. ^222^Vascular Medicine, Georges Pompidou Hospital, APHP, Paris, France. ^223^Institut Jérôme Lejeune, Paris, France. ^224^Division of Pulmonary and Critical Care, College of Medicine-Jacksonville, University of Florida, Jacksonville, FL, USA. ^225^Department of Pediatrics, Hiroshima University Graduate School of Biomedical and Health Sciences, Hiroshima, Japan. ^226^BC Children’s Hospital Research Institute, University of British Columbia, Vancouver, Canada. ^227^Médecine Intensive Réanimation, Hôpitaux Universitaires Henri Mondor, Assistance Publique – Hôpitaux de Paris (AP-HP), Créteil, France. ^228^Guanarteme Health Care Center, Canarian Health System, Las Palmas de Gran Canaria, Spain. ^229^Regional University Hospital of Malaga, Malaga, Spain. ^230^Department of Immunology, Hospital Universitari de Bellvitge, IDIBELL, Barcelona, Spain. ^231^Aix Marseille Univ, INSERM, INRAE, C2VN, Marseille, France. ^232^Department of General Paediatrics, Hôpital Bicêtre, AP-HP, University of Paris Saclay, Le Kremlin-Bicêtre, France. ^233^INSERM U1144, Université de Paris, DMU INVICTUS, APHP-Nord, Département de Médecine Interne, Lariboisière Hospital, Paris, France. ^234^CHU de La Timone, Marseille, France. ^235^Department of Epidemiology, Infectious Disease Control and Prevention, Graduate School of Biomedical and Health Sciences, Hiroshima University, Hiroshima, Japan. ^236^Pediatric Immunology and rhumatology Department,Necker Hospital, AP-HP, Paris, France. ^237^Centro Hospitalar Universitário de Lisboa Central, Lisbon, Portugal. ^238^Infectious Diseases Horizontal Technlogy Centre, A*STAR; Singapore Immunology Network, A*STAR, Singapore. ^239^Department of Medicine and Geriatrics, Tuen Mun Hospital, Hong Kong. ^240^Regional Universitary Hospital of Malaga, Málaga, Spain. ^241^Department of Immunology, Hospital Universitario Marqués de Valdecilla, Santander, Spain. ^242^Bilkent University, Department of Molecular Biology and Genetics, Ankara, Turkey. ^243^BRFAA, Athens, Greece. ^244^IHU Méditerranée Infection, Aix Marseille Univ, IRD, AP-HM, SSA, VITROME, IHU Méditerranée Infection, Marseille, France. ^245^L’Hôpital Foch, Suresnes, France. ^246^Department of Immunology, CIC1408, GIMAP CIRI INSERM U1111, University Hospital of Saint-Etienne, St Etienne, France. ^247^Department of Immunology, Hospital Universitario 12 de Octubre, Instituto de Investigación Sanitaria Hospital 12 de Octubre (imas12), Madrid, Spain. ^248^Mexico. ^249^Diabetes Research Institute, IRCCS San Raffaele Hospital, Milan, Italy. ^250^APHP Hôpitaux Universitaires Paris-Sud, Le Kremlin-Bicêtre, France. ^251^AP-HP, Avicenne Hospital, Intensive Care Unit, Bobigny, France; INSERM UMR-S 942, Cardiovascular Markers in Stress Conditions (MASCOT), University of Paris, Paris, France. ^252^Neurometabolic Diseases Laboratory, IDIBELL-Hospital Duran i Reynals, Barcelona; CIBERER U759, ISCiii Madrid, Spain. ^253^Hospices Civils de Lyon, Lyon, France. ^254^Univ. Lille, INSERM U1285, CHU Lille, Pôle de médecine intensive-réanimation, CNRS, UMR 8576 - Unité de Glycobiologie Structurale et Fonctionnelle, Lille, France. ^255^Department of General pediatrics, Robert Debre Hospital, Paris, France. ^256^University of British Columbia, Vancouver, Canada. ^257^Jeffrey Modell Diagnostic and Research Center for Primary Immunodeficiencies, Barcelona, Catalonia, Spain, Diagnostic Immunology Research Group, Vall d’Hebron Research Institute (VHIR), Vall d’Hebron University Hospital (HUVH), Vall d’Hebron Barcelona Hospital Campus, Barcelona, Catalonia, Spain. ^258^AP-HP, Avicenne Hospital, Intensive Care Unit, Bobigny, France; University Sorbonne Paris Nord, Bobigny, France. ^259^Centre Hospitalier de Saint-Denis, St Denis, France. ^260^Precision Medicine Unit, Lausanne University Hospital and University of Lausanne, Lausanne, Switzerland. ^261^Paris Cardiovascular Center, PARCC, INSERM, Université de Paris, Paris, France. ^262^Germans Trias i Pujol Hospital, Badalona, Spain. ^263^Medical intensive care unit. Hopital de la Croix-Rousse. Hospices Civils de Lyon, Lyon, France. ^264^Department of Clinical Laboratory, Hospital Universitari de Bellvitge, IDIBELL, Barcelona, Spain. ^265^Pediatric Infectious Diseases and Immunodeficiencies Unit, Hospital Universitari Vall d’Hebron, Vall d’Hebron Research Institute, Vall d’Hebron Barcelona Hospital Campus, Barcelona, Spain. ^266^Université de Paris, CNRS UMR-8601; Team Chemistry & Biology, Modeling & Immunology for Therapy, CBMIT, Paris, France. ^267^Germans Trias i Pujol University Hospital and Research Institute. Badalona, Badalona, Spain. ^268^Department of Immunology, University Hospital of Gran Canaria Dr. Negrín, Canarian Health System, Las Palmas de Gran Canaria, Spain; Department of Clinical Sciences, University Fernando Pessoa Canarias, Las Palmas de Gran Canaria, Spain. ^269^Neurometabolic Diseases Laboratory, Bellvitge Biomedical Research Institute (IDIBELL), 08908 L’Hospitalet de Llobregat; University Hospital Germans Trias i Pujol, Badalona, Barcelona, Catalonia, Spain. ^270^Consorcio Hospital General Universitario, Valencia, Spain. ^271^APHP Hôpitaux Universitaires Paris-Sud, Paris, France. ^272^Intensive Care Unit, Louis-Mourier Hospital, Colombes, France. ^273^Virology unit, Université de Paris, Cohin Hospital, APHP, Paris, France. ^274^Neurometabolic Diseases Laboratory and CIBERER U759, Barcelona, Spain. ^275^Hospital San Pedro, Logroño, Spain. ^276^University of Tartu, Institute of Biomedicine and Translational Medicine, Tartu, Estonia. ^277^Respiratory medicine, Georges Pompidou Hospital, APHP, Paris, France. ^278^Infectious Diseases Department, International Health Program of the Catalan Insitute of Health (PROSICS), Vall d’Hebron University Hospital (HUVH), Vall d’Hebron Barcelona Hospital Campus, Universitat Autónoma de Barcelona, Barcelona, Spain. ^279^Hospital Clínico San Carlos and IdSSC, Madrid, Spain. ^280^Faculdades Pequeno Príncipe, Instituto de Pesquisa Pelé Pequeno Príncipe, Curitiba, Brazil. ^281^AP-HP, Avicenne Hospital, Intensive Care Unit, Bobigny, France. ^282^Service de Médecine Intensive Réanimation, Institut de Cardiologie, Hopital Pitié-Salpêtrière, Paris, France. ^283^Department of Pediatrics, Medizinische Fakultät Carl Gustav Carus, Technische Universität Dresden, Dresden, Germany. ^284^CHRU de Nancy, Hôpital d’Enfants, Vandoeuvre, France. ^285^Chair of Nephrology, University of Brescia, Brescia, Italy. ^286^Department of Immunology, 2nd Faculty of Medicine, Charles University and Motol University Hospital, Prague, Czech Republic. ^287^Clínica Universidad de Navarra and Ciberes, Madrid, Spain. ^288^HUS Helsinki University Hospital, Children and Adolescents, Rare Disease Center, and Inflammation Center, Adult Immunodeficiency Unit, Majakka, Helsinki, Finland. ^289^Fundació Docencia i Recerca Mutua Terrassa, Terrassa, Spain. ^290^D. Rogachev National Medical and Research Center of Pediatric Hematology, Oncology, Immunoogy, Moscow, Russia. ^291^Haematology Laboratory, Lariboisière Hospital, University of Paris, Paris, France. ^292^Biomedical Research Foundation of the Academy of Athens. ^293^INSERM U1140, University of Paris, European Georges Pompidou Hospital, Paris, France. ^294^Department of Pediatrics, Faculty of Medicine, Mansoura University, Mansoura, Egypt. ^295^Critical Care Unit , Hospital Universitario de Gran Canaria Dr. Negrín, Canarian Health System, Las Palmas de Gran Canaria, Spain. ^296^CHU de Saint Etienne, Saint-Priest-en-Jarez, France. ^297^Pediatric Infectious Diseases and Immunodeficiencies Unit, Hospital Universitari Vall d’Hebron, Vall d’Hebron Research Institute, Vall d’Hebron Barcelona Hospital Campus. Universitat Autònoma de Barcelona (UAB). Barcelona, Catalonia, Spain, EU., Barcelona, Spain. ^298^Department of pediatric infectious diseases and pediatric immunology, Shupyk National Healthcare University of Ukraine, Kyiv, Ukraine. ^299^Gustave Roussy Cancer Campus, Villejuif, France. ^300^Intensive Care Unit, Avicenne Hospital, APHP, Bobigny, France. ^301^Laboratory of Immunology and Histocompatibility, Saint-Louis Hospital, Paris University, Paris, France. ^302^ Center for Inflammation Research, Laboratory of Molecular Signal Transduction in Inflammation, VIB, Ghent, Belgium. ^303^Department of Internal Medicine, Université de Paris, INSERM, U970, PARCC, F-75015, Paris, France. ^304^Service de médecine intensive réanimation, CHU de Saint-Etienne, France. ^305^Dept of Rheumatology and Inflammation Research, Institute of Medicine, Sahlgrenska Academy, University of Gothenburg, Gothenburg, Sweden. ^306^University of Management and Technology, Lahore, Pakistan. ^307^Department of Infectious Diseases, Aarhus University Hospital, Aarhus, Denmark. ^308^First Division of Anesthesiology and Critical Care Medicine, University of Brescia, ASST Spedali Civili di Brescia, Brescia, Italy. ^309^Intensive Care Department, Hospital Universitari MutuaTerrassa, Universitat Barcelona, Terrassa, Spain. ^310^Laboratory of Immunobiology, Center for Clinical, Experimental Surgery and Translational Research, Biomedical Research Foundation of the Academy of Athens, Athens, Greece. ^311^International Center of Research in Infectiology, Lyon University, INSERM U1111, CNRS UMR 5308, ENS, UCBL, Lyon, France; Hospices Civils de Lyon, Lyon Sud Hospital, Pierre-Bénite, France. ^312^Infanta Leonor University Hospital, Madrid, Spain. ^313^Department of Medicine and Geriatrics, Princess Margaret Hospital, Hong Kong. ^314^University of Tartu, Institute of Clinical Medicine, Tartu, Estonia. ^315^Department of Medicine, United Christian Hospital, Hong Kong. ^316^Hematology Department, ASST Spedali Civili di Brescia, Brescia, Italy. ^317^Pneumologie, Hôpital Avicenne, APHP, INSERM U1272, Université Sorbonne Paris Nord, Bobigny, France. ^318^Dermatology unit, Laboratoire GAD, INSERM UMR1231 LNC, université de Bourgogne, Dijon, France. ^319^University Hospital of Burgos, Burgos, Spain. ^320^Center of Human Genetics, Hôpital Erasme, Université Libre de Bruxelles, Brussels, Belgium. ^321^Bellvitge University Hospital, L’Hospitalet de Llobregat, Barcelona, Spain. ^322^University of São Paulo, São Paulo, Brazil. ^323^CHU de Caen, Caen, France. ^324^Hospital del Mar - IMIM Biomedical Research Institute, Barcelona, Catalonia, Spain. ^325^Neglected Human Genetics Laboratory, INSERM, University of Paris, Paris, France. ^326^Sorbonne Université, Service de Médecine Intensive Réanimation, Hôpital Tenon, Assistance Publique-Hôpitaux de Paris, Paris, France. ^327^Pediatric Infectious Disease and Pediatric Immunology Department, Shupyk National Healthcare University of Ukraine, Kyiv, Ukraine. ^328^Department of Pneumology, University Hospitals Leuven, Leuven, Belgium. ^329^Laboratory for Clinical Infectious and Inflammatory Disorders, Departement of Microbiology, Immunology and Transplantation, Leuven, Belgium. ^330^Department of Clinical Pathology, Pamela Youde Nethersole Eastern Hospital, Hong Kong. ^331^Department of Medicine, Queen Elizabeth Hospital, Hong Kong. ^332^Ankara City Hospital, Children’s Hospital, Ankara, Turkey. ^333^Division of Pediatric Infectious Disease, Department of Pediatrics, Faculty of Medicine, Karadeniz Technical University, Trabzon, Turkey. ^334^Health Sciences University, Lütfi Kırdar Kartal Education and Research Hospital, İstanbul, Turkey. ^335^Department of Nephrology and Infectiology, AZ Sint-Jan, Bruges, Belgium. ^336^Department of Pulmonology, Ghent University Hospital, Belgium. ^337^Department of Pediatric pulmonology and immunology, Ghent University Hospital, Belgium. ^338^Department of Intensive Care Unit, Ghent University Hospital, Belgium. ^339^Department of Pediatric hemato-oncology, Jolimont Hospital; Department of Pediatric hemato-oncology, HUDERF, Brussels, Belgium. ^340^Department of Pulmonology, ZNA Middelheim, Antwerp, Belgium. ^341^Department of Internal Medicine, Ghent University Hospital, Belgium. ^342^Department of Pediatric immuno-hémato-rhumatology, CHR Citadelle, Liége, Belgium. ^343^Department of Pediatric hemato-oncology, UCL Louvain, Belgium. ^344^Department of Pediatrics, Saint Luc, UCL Louvain, Belgium.

### COVID-STORM Clinicians

Giuseppe Foti^1^, Giacomo Bellani^1^, Giuseppe Citerio^1^, Ernesto Contro^1^, Alberto Pesci^2^, Maria Grazia Valsecchi^3^, Marina Cazzaniga^4^

^1^Department of Emergency, Anesthesia and Intensive Care, School of Medicine and Surgery, University of Milano-Bicocca, San Gerardo Hospital, Monza, Italy. ^2^Department of Pneumology, School of Medicine and Surgery, University of Milano-Bicocca, San Gerardo Hospital, Monza, Italy. ^3^Center of Bioinformatics and Biostatistics, School of Medicine and Surgery, University of Milano-Bicocca, San Gerardo Hospital, Monza, Italy. ^4^Phase I Research Center, School of Medicine and Surgery, University of Milano-Bicocca, San Gerardo Hospital, Monza, Italy.

### NIAID Immune Response to COVID Group

Jeffrey J. Danielson^1^, Kerry Dobbs^1^, Anuj Kashyap^1^, Li Ding^1^, Clifton L. Dalgard^2^, Alessandra Sottini^3^, Virginia Quaresima^3^, Eugenia Quiros-Roldan^4^, Camillo Rossi^5^, Laura Rachele Bettini^6^, Mariella D’Angio’^6^, Ilaria Beretta^7^, Daniela Montagna^8^, Amelia Licari^9^, Gian Luigi Marseglia^10^

^1^Laboratory of Clinical Immunology and Microbiology, Division of Intramural Research, NIAID, NIH, Bethesda, MD, USA. ^2^Department of Anatomy, Physiology & Genetics, Uniformed Services University of the Health Sciences; The American Genome Center, Uniformed Services University of the Health Sciences, Bethesda, MD, USA. ^3^CREA Laboratory, Diagnostic Department, ASST Spedali Civili di Brescia, Brescia, Italy. ^4^Department of Infectious and Tropical Diseases, University of Brescia and ASST Spedali Civili di Brescia, Brescia, Italy. ^5^Chief Medical Officer, ASST Spedali Civili di Brescia, Brescia, Italy. ^6^Pediatric Departement and Centro Tettamanti-European Reference Network PaedCan, EuroBloodNet, MetabERN-University of Milano-Bicocca-Fondazione MBBM-Ospedale, San Gerardo, Monza, Italy. "^7^Department of Infectious Diseases, University of Milano-Bicocca, San Gerardo Hospital, Monza, Italy. " ^8^Laboratory of Immunology and Transplantation, Fondazione IRCCS Policlinico San Matteo, Pavia, Italy; Department of Clinical, Surgical, Diagnostic and Pediatric Sciences, University of Pavia, Pavia, Italy. ^9^Department of Pediatrics, Fondazione IRCCS Policlinico San Matteo, University of Pavia, Pavia, Italy. ^10^Department of Pediatrics, Fondazione IRCCS Policlinico San Matteo, University of Pavia, Pavia, Italy; Department of Clinical, Surgical, Diagnostic and Pediatric Sciences, University of Pavia, Pavia, Italy.

### NH-COVAIR Study Group

Isabella Batten^1^, Conor Reddy^1^, Matt McElheron^1^, Claire Noonan^1^, Emma Connolly^1^, Aoife Fallon^1^

^1^Department of Age-Related Healthcare, Tallaght University Hospital & Department of Medical Gerontology, School of Medicine, Trinity College Dublin

### Danish CHGE

Merete Storgaard^1^, Sofie Jørgensen^1^, Martin Tolstrup^1^

^1^Dept. Infectious Diseases, Aarhus University Hospital, Aarhus, Denmark.

### The Danish Blood Donor Study (DBDS)

Christian Erikstrup^1^, Ole Birger Pedersen^2^, Erik Sørensen^3^, Susan Mikkelsen^1^, Khoa Manh Dinh^1^, Margit Anita Hørup Larsen^3^, Isabella Worlewenut Paulsen^2^, Jakob Hjorth Von Stemann^3^, Morten Bagge Hansen^3^, Sisse Rye Ostrowski^3^

^1^Dept. Clinical Immunology, Aarhus University Hospital, Aarhus, Denmark. ^2^Dept. Clinical Immunology, Zeeland University Hospital, Køge, Denmark, ^3^Dept. of Clinical Immunology, Rigshospitalet, Copenhagen University Hospital, Copenhagen, Denmark

### St. James’s Hospital, SARS CoV2 Interest group

Liam Townsend^1^, Cliona Ni Cheallaigh^1^, Colm Bergin^1^, Ignacio Martin-Loeches^2^, Jean Dunne^3^, Niall Conlon^3^, Nollaig Bourke^4^, Cliona O’Farrelly^5^

^1^Department of Infectious Diseases, St. James’s Hospital; Department of Clinical Medicine, School of Medicine, Trinity Translational Medicine Institute, Trinity College Dublin, Dublin, Ireland. ^2^Department of Intensive Care Medicine, St James’s Hospital, Dublin, Ireland. ^3^Department of Immunology, St. James’s Hospital; Department of Immunology, School of Medicine, Trinity College Dublin, Ireland. ^4^Department of Medical Gerontology, School of Medicine, Trinity Translational Medicine Institute, Trinity College Dublin, Dublin, Ireland. ^5^School of Biochemistry and Immunology, Trinity Biomedical Sciences Institute, Trinity College Dublin; School of Medicine, Trinity College Dublin, Dublin, Ireland.

### French COVID Cohort Study Group

Laurent ABEL^1^, Clotilde ALLAVENA^2^, Claire ANDREJAK^3^, François ANGOULVANT^4^, Cecile AZOULAY^5^, Delphine BACHELET^6^, Marie BARTOLI^7^, Romain BASMACI^8^, Sylvie BEHILILL^9^, Marine BELUZE^10^, Nicolas BENECH^11^, Dehbia BENKERROU^12^, Krishna BHAVSAR^6^, Laurent BITKER^11^, Lila BOUADMA^6^, Maude BOUSCAMBERT-DUCHAMP^13^, Pauline CARAUX PAZ^14^, Minerva CERVANTES-GONZALEZ^6^, Anissa CHAIR^6^, Catherine CHIROUZE^15^, Alexandra COELHO^16^, Hugues CORDEL^17^, Camille COUFFIGNAL^6^, Sandrine COUFFIN-CADIERGUES^18^, Eric d’ORTENZIO^7^, Etienne DE MONTMOLLIN^6^, Alexa DEBARD^19^, Marie-Pierre DEBRAY^6^, Dominique DEPLANQUE^20^, Diane DESCAMPS^6^, Mathilde DESVALLÉE^21^, Alpha DIALLO^7^, Jean-Luc DIEHL^22^, Alphonsine DIOUF^16^, Céline DORIVAL^12^, François DUBOS^23^, Xavier DUVAL^6^, Philippine ELOY^6^, Vincent ENOUF^9^, Olivier EPAULARD^24^, Hélène ESPEROU^18^, Marina ESPOSITO-FARESE^6^, Manuel ETIENNE^25^, Denis GAROT^26^, Nathalie GAULT^6^, Alexandre GAYMARD^13^, Jade GHOSN^6^, Tristan GIGANTE^27^, Morgane GILG^27^, François GOEHRINGER^28^, Jérémie GUEDJ^29^, Alexandre HOCTIN^16^, Isabelle HOFFMANN^6^, Ikram HOUAS^18^, Jean-Sébastien HULOT^22^, Salma JAAFOURA^18^, Ouifiya KAFIF^6^, Florentia KAGUELIDOU^30^, Sabrina KALI^6^, Younes KERROUMI^31^, Antoine KHALIL^6^, Coralie KHAN^21^, Antoine KIMMOUN^32^, Fabrice LAINE^33^, Cédric LAOUÉNAN^6^, Samira LARIBI^6^, Minh LE^6^, Cyril LE BRIS^34^, Sylvie LE GAC^6^, Quentin LE HINGRAT^6^, Soizic LE MESTRE^7^, Hervé LE NAGARD^35^, Adrien LEMAIGNEN^26^, Véronique LEMEE^25^, François-Xavier LESCURE^6^, Sophie LETROU^6^, Yves LEVY^36^, Bruno LINA^13^, Guillaume LINGAS^35^, Jean Christophe LUCET^6^, Moïse MACHADO^37^, Denis MALVY^38^, Marina MAMBERT^16^, Aldric MANUEL^39^, France MENTRÉ^6^, Amina MEZIANE^12^, Hugo MOUQUET^9^, Jimmy Mullaert^6^, Nadège NEANT^35^, Duc NGUYEN^38^, Marion NORET^40^, Aurélie PAPADOPOULOS^18^, Christelle PAUL^7^, Nathan PEIFFER-SMADJA^6^, Vincent PEIGNE^41^, Ventzislava PETROV-SANCHEZ^7^, Gilles PEYTAVIN^6^, Huong PHAM^6^, Olivier PICONE^8^, Valentine PIQUARD^6^, Julien POISSY^23^, Oriane PUÉCHAL^42^, Manuel ROSA-CALATRAVA^13^, Bénédicte ROSSIGNOL^27^, Patrick ROSSIGNOL^28^, Carine ROY^6^, Marion SCHNEIDER^6^, Richa SU^6^, Coralie TARDIVON^6^, Marie-Capucine TELLIER^6^, François TÉOULÉ^12^, Olivier TERRIER^13^, Jean-François TIMSIT^6^, Christelle TUAL^43^, Sarah TUBIANA^6^, Sylvie VAN DER WERF^9^, Noémie VANEL^44^, Aurélie VEISLINGER^43^, Benoit VISSEAUX^6^, Aurélie WIEDEMANN^45^, Yazdan YAZDANPANAH^6^

^1^INSERM UMR 1163, Paris, France. ^2^CHU Nantes, France. ^3^CHU Amiens, France. ^4^Hôpital Necker, Paris, France. ^5^Hopitâl Cochin, Paris, France. ^6^Hôpital Bichat, Paris, France. ^7^ANRS, Paris, France. ^8^Hôpital Louis Mourier, Colombes, France. ^9^Pasteur Institute, Paris, France. ^10^F-CRIN Partners Platform, Paris, France. ^11^CHU Lyon, France. ^12^INSERM UMR 1136, Paris, France. ^13^INSERM UMR 1111, Lyon, France. ^14^CH Villeneuve Saint Georges, France. ^15^CHRU Jean Minjoz, Besançon, France. ^16^INSERM UMR 1018, Paris, France. ^17^Hôpital Avicenne, Bobigny, France. ^18^INSERM sponsor, Paris, France. ^19^CHU Toulouse, France. ^20^Hôpital Calmette, Lille, France. ^21^INSERM UMR 1219, Bordeaux, France. ^22^Hôpital Européen Georges Pompidou, Paris, France. ^23^CHU Lille, France. ^24^CHU Grenoble, France. ^25^CHU Rouen, France. ^26^CHU Tours, France. ^27^F-CRIN INI-CRCT, Nancy, France. ^28^CHU Nancy, France. ^29^Université de Paris, INSERM, IAME, F-75018 Paris, France. ^30^Hôpital Robert Debré, Paris, France. ^31^GH Diaconesses, Paris, France. ^32^Université de Lorraine, CHRU de Nancy, Service de Médecine Intensive et Réanimation Brabois, INSERM U116, Nancy, France. ^33^CHU Rennes, France. ^34^CH Beziers, France. ^35^INSERM UMR 1137, Paris, France. ^36^Vaccine Research Insitute (VRI), INSERM U955, Créteil, France. ^37^Grand Hôpital de l’Est Francilien, Marne-la-Vallée, France. ^38^CHU Bordeaux, France. ^39^CH Annecy, France. ^40^RENARCI, Annecy, France. ^41^CH Métropole Savoie, Cambery, France. ^42^REACTing, Paris, France. ^43^INSERM CIC-1414, Rennes, France. ^44^Hôpital la Timone, Marseille, France. ^45^Vaccine Research Insitute (VRI), INSERM UMR 955, Créteil, France.

### Imagine COVID-Group

Jean-Philippe Annereau^1^, Luis Briseño-Roa^1^, Olivier Gribouval^2^, Anna Pelet^2^

^1^Medetia Pharmaceuticals, Paris, France. ^2^Imagine Institute, Université de Paris, INSERM UMR 1163, Paris, France.

### The Milieu Intérieur Consortium

Laurent Abel^1^, Andres Alcover^2^, Hugues Aschard^2^, Philippe Bousso^2^, Nollaig Bourke^3^, Petter Brodin^4^, Pierre Bruhns^2^, Nadine Cerf-Bensussan^5^, Ana Cumano^2^, Christophe D’Enfert^2^, Ludovic Deriano^2^, Marie-Agnès Dillies^2^, James Di Santo^2^, Françoise Dromer^2^, Gérard Eberl^2^, Jost Enninga^2^, Jacques Fellay^6^, Ivo Gomperts-Boneca^2^, Milena Hasan^2^, Gunilla Karlsson Hedestam^4^, Serge Hercberg^7^, Molly A Ingersoll^2^, Olivier Lantz^8^, Rose Anne Kenny^3^, Mickaël Ménager^5^, Frédérique Michel^2^, Hugo Mouquet^2^, Cliona O’Farrelly^3^, Etienne Patin^2^, Sandra Pellegrini^2^, Antonio Rausell^5^, Frédéric Rieux-Laucat^5^, Lars Rogge^2^, Magnus Fontes^9^, Anavaj Sakuntabhai^2^, Olivier Schwartz^2^, Benno Schwikowski^2^, Spencer Shorte^2^, Frédéric Tangy^2^, Antoine Toubert^10^, Mathilde Touvier^12^, Marie-Noëlle Ungeheuer^2^, Christophe Zimmer^2^, Matthew L. Albert^11^, Darragh Duffy^2^, Lluis Quintana-Murci^2^

^1^Hôpital Necker, Paris, France. ^2^Institut Pasteur, Paris, France. ^3^Trinity College, Dublin, Ireland. ^4^Karolinska Institutet, Stockholm, Sweden. ^5^INSERM U1163, Institut Imagine, Paris, France. ^6^EPFL, Lausanne, Switzerland. ^7^Université Paris ^13^, Paris, France. ^8^Institut Curie, Paris, France. ^9^Institut Roche, Paris, France. ^10^Hôpital Saint-Louis, Paris, France. ^11^In Sitro, San Francisco, USA. ^12^Sorbonne Paris Nord University, INSERM U1153, INRAE U1125, CNAM, Nutritional Epidemiology Research Team (EREN), Epidemiology and Statistics Research Center – University of Paris (CRESS), Bobigny, France.

### CoV-Contact Cohort

Loubna Alavoine^1^, Sylvie Behillil^2^, Charles Burdet^3^, Charlotte Charpentier^3,4^, Aline Dechanet^5^, Diane Descamps^3,6^, Xavier Duval^1,3^, Jean-Luc Ecobichon^1^, Vincent Enouf^8^, Wahiba Frezouls^1^, Nadhira Houhou^5^, Ouifiya Kafif^5^, Jonathan Lehacaut^1^, Sophie Letrou^1^, Bruno Lina^9^, Jean-Christophe Lucet^10^, Pauline Manchon^5^, Mariama Nouroudine^1^, Valentine Piquard^5^, Caroline Quintin^1^, Michael Thy^11^, Sarah Tubiana^1^, Sylvie van der Werf^8^, Valérie Vignali^1^, Benoit Visseaux^3,10^, Yazdan Yazdanpanah^3,10^, Abir CHAHINE^12^, Nawal WAUCQUIER^12^, Maria-Claire MIGAUD^12^, Dominique DEPLANQUE^12^, Félix DJOSSOU^13^, Mayka Mergeay-Fabre^14^, Aude LUCARELLI^15^, Magalie DEMAR^13^, Léa Bruneau^16^, Patrick Gérardin^17^, Adrien Maillot^16^, Christine Payet^18^, Bruno Laviolle^19^, Fabrice Laine^19^, Christophe Paris^19^, Mireille Desille-Dugast^19^, Julie Fouchard^19^, Denis MALVY^20^, Duc NGUYEN^20^, Thierry PISTONE^20^, Pauline PERREAU^20^, Valérie GISSOT^21^, Carole LE GOAS^21^, Samatha Montagne^22^, Lucie Richard^23^, Catherine Chirouze^24^, Kévin Bouiller^24^, Maxime Desmarets^25^, Alexandre Meunier^26^, Benjamin Lefévre^27^, Hélène Jeulin^28^, Karine Legrand^29^, Sandra Lomazzi^30^, Bernard Tardy^31^, Amandine Gagneux-Brunon^32^, Frédérique Bertholon^33^, Elisabeth Botelho-Nevers^32^, KOUAKAM Christelle KOUAKAM Christelle^34^, LETURQUE Nicolas LETURQUE Nicolas^34^, Layidé Roufai^34^, Karine Amat^35^, Sandrine Couffin-Cadiergues^34^, Hélène Espérou^36^, Samia Hendou^34^

^1^Centre d’Investigation Clinique, INSERM CIC 1425, Hôpital Bichat Claude Bernard, APHP, Paris, France. ^2^Institut Pasteur, Paris, France. ^3^Université de Paris, IAME, INSERM U1137, Paris, France, Hôpital Bichat Claude Bernard, APHP, Paris, France. ^4^ Service de Virologie, Université de Paris, INSERM, IAME, UMR 1137, AP-HP, Hôpital Bichat-Claude Bernard, F-75018 Paris, France. ^6^IAME INSERM U1140, Hôpital Bichat Claude Bernard, APHP, Paris, France. ^7^Centre d’Investigation Clinique, INSERM CIC 1425, APHP, IAME, Paris University, Paris, France. ^8^Institut Pasteur, U3569 CNRS, Université de Paris, Paris, France. ^9^Virpath Laboratory, International Center of Research in Infectiology, Lyon University, INSERM U1111, CNRS U5308, ENS, UCBL, Lyon, France. ^10^IAME INSERM U1138, Hôpital Bichat Claude Bernard, APHP, Paris, France. ^11^Center for Clinical Investigation, Assistance Publique-Hôpitaux de Paris, Bichat-Claude Bernard University Hospital, Paris, France. ^12^Centre d’Investigation Clinique, INSERM CIC 1403, Centre Hospitalo universitaire de Lille, Lille, France. ^13^Service des maladies infectieuses, Centre Hospitalo universitaire de Cayenne, Guyane, France. ^14^Centre d’Investigation Clinique, INSERM CIC 1424, Centre Hospitalier de Cayenne, Cayenne, Guyane Française. ^15^Service Hôpital de jour Adulte, Centre Hospitalier de Cayenne, Guyane, France. ^16^Centre d’Investigation Clinique, INSERM CIC 1410, Centre Hospitalo universitaire de la Réunion, La Réunion, France. ^17^Centre d’Investigation Clinique, INSERM CIC 1410, CHU Reunion, Saint-Pierre, Reunion island. ^18^Centre d’Investigation Clinique, INSERM CIC 1410, Centre de Ressources Biologiques, Centre Hospitalo universitaire de la Réunion, La Réunion, France. ^19^Centre d’Investigation Clinique, INSERM CIC 1414, Centre Hospitalo universitaire de Rennes, Rennes, France. ^20^Service des maladies infectieuses, Centre Hospitalo universitaire de Bordeaux, Bordeaux, France. ^21^Centre d’Investigation Clinique, INSERM CIC 1415, CHRU Tours, Tours, France. ^22^CRBT, Centre Hospitalo universitaire de Tours, Tours, France. ^23^Pole de Biologie Médicale, Centre Hospitalo universitaire de Tours, Tours, France. ^24^Service des maladies infectieuses, Centre Hospitalo universitaire de Besançon, Besançon, France. ^25^Service des maladies infectieuses, Centre d’investigation clinique, INSERM CIC1431, Centre Hospitalier Universitaire de Besançon, Besançon, France. ^26^Centre de Ressources Biologiques - Filière Microbiologique de Besançon, Centre Hospitalier Universitaire, Besançon, France. ^27^Université de Lorraine, CHRU-Nancy and APEMAC, Infectious and tropical diseases, Nancy, France. ^28^Laboratoire de Virologie, CHRU de Nancy Brabois, Vandoeuvre-lès-Nancy, France. ^29^INSERM CIC-EC 1433, Centre Hospitalo universitaire de Nancy, Nancy, France. ^30^Centre de ressources Biologiques, Centre Hospitalo universitaire de Nancy, Nancy, France. ^31^Centre d’Investigation Clinique, INSERM CIC 1408, Centre Hospitalo universitaire de Saint Etienne, Saint Etienne, France. ^32^Service des maladies infectieuses, Centre Hospitalo universitaire de Saint Etienne, Saint Etienne, France. ^33^Service des maladies infectieuses, CRB^42^-BTK, Centre Hospitalo Universitaire de Saint Etienne, Saint Etienne, France. ^34^Pole Recherche Clinique, INSERM, Paris France. ^35^IMEA Fondation Léon M’Ba, Paris, France. ^36^INSERM Pôle Recherche Clinique, Paris, France.

### Amsterdam UMC Covid-19 Biobank Investigators

Michiel van Agtmael^2^, Anne Geke Algera^1^, Brent Appelman^2^, Frank van Baarle^1^, Diane Bax^3^, Martijn Beudel^4^, Harm Jan Bogaard^5^, Marije Bomers^2^, Peter Bonta^5^, Lieuwe Bos^1^, Michela Botta^1^, Justin de Brabander^2^, Godelieve de Bree^2^, Sanne de Bruin^1^, David T.P. Buis^1^, Marianna Bugiani^5^, Esther Bulle^1^, Osoul Chouchane^2^ Alex Cloherty^3^, Mirjam Dijkstra^12^, Dave A. Dongelmans^1^, Romein W.G. Dujardin^1^, Paul Elbers^1^, Lucas Fleuren^1^, Suzanne Geerlings^2^ Theo Geijtenbeek^3^, Armand Girbes^1^, Bram Goorhuis^2^, Martin P. Grobusch^2^, Florianne Hafkamp^3^, Laura Hagens^1^, Jorg Hamann^7^, Vanessa Harris^2^, Robert Hemke^8^, Sabine M. Hermans^2^ Leo Heunks^1^, Markus Hollmann^6^, Janneke Horn^1^, Joppe W. Hovius^2^, Menno D. de Jong^9^, Rutger Koning^4^, Endry H.T. Lim^1^, Niels van Mourik^1^, Jeaninne Nellen^2^, Esther J. Nossent^5^, Frederique Paulus^1^, Edgar Peters^2^, Dan A.I. Pina-Fuentes^4^, Tom van der Poll^2^, Bennedikt Preckel^6^, Jan M. Prins^2^, Jorinde Raasveld^1^, Tom Reijnders^2^, Maurits C.F.J. de Rotte^12^, Michiel Schinkel^2^, Marcus J. Schultz^1^, Femke A.P. Schrauwen^12^, Alex Schuurmans^10^, Jaap Schuurmans^1^, Kim Sigaloff^1^, Marleen A. Slim^1,2^, Patrick Smeele^5^, Marry Smit^1^, Cornelis S. Stijnis^2^, Willemke Stilma^1^, Charlotte Teunissen^11^, Patrick Thoral^1^, Anissa M Tsonas^1^, Pieter R. Tuinman^2^, Marc van der Valk^2^, Denise Veelo^6^, Carolien Volleman^1^, Heder de Vries^1^, Lonneke A. Vught^1,2^, Michèle van Vugt^2^, Dorien Wouters^12^, A. H (Koos) Zwinderman^13^, Matthijs C. Brouwer^4^, W. Joost Wiersinga^2^, Alexander P.J. Vlaar^1^, Diederik van de Beek^4^.

^1^Department of Intensive Care, Amsterdam UMC, Amsterdam, The Netherlands; ^2^Department of Infectious Diseases, Amsterdam UMC, Amsterdam, The Netherlands; ^3^Experimental Immunology, Amsterdam UMC, Amsterdam, The Netherlands; ^4^Department of Neurology, Amsterdam UMC, Amsterdam Neuroscience, Amsterdam, The Netherlands; ^5^Department of Pulmonology, Amsterdam UMC, Amsterdam, The Netherlands; ^6^Department of Anesthesiology, Amsterdam UMC, Amsterdam, The Netherlands; ^7^Amsterdam UMC Biobank Core Facility, Amsterdam UMC, Amsterdam, The Netherlands; ^8^Department of Radiology, Amsterdam UMC, Amsterdam, The Netherlands; ^9^Department of Medical Microbiology, Amsterdam UMC, Amsterdam, The Netherlands; ^10^Department of Internal Medicine, Amsterdam UMC, Amsterdam, The Netherlands; ^11^Neurochemical Laboratory, Amsterdam UMC, Amsterdam, The Netherlands; ^12^Department of Clinical Chemistry, Amsterdam UMC, Amsterdam, The Netherlands; ^13^Department of Clinical Epidemiology, Biostatistics and Bioinformatics, Amsterdam UMC, Amsterdam, The Netherlands.

### COVID Human Genetic Effort

Laurent Abel^1^, Alessandro Aiuti^2^, Saleh Al-Muhsen^3^, Fahd Al-Mulla^4^, Mark S. Anderson^5^, Evangelos Andreakos^6^, Andrés A. Arias^7^, Hagit Baris Feldman^8^, Alexandre Belot^9^, Catherine M. Biggs^10^, Dusan Bogunovic^11^, Alexandre Bolze^12^, Anastasiia Bondarenko^13^, Ahmed A. Bousfiha^14^, Petter Brodin^15^, Yenan Bryceson^16^, Carlos D. Bustamante^17^, Manish J. Butte^18^, Giorgio Casari^19^, Samya Chakravorty^20^, John Christodoulou^21^, Antonio Condino-Neto^22^, Stefan N. Constantinescu^23^, Megan A. Cooper^24^, Clifton L. Dalgard^25^, Murkesh Desai^26^, Beth A. Drolet^27^, Jamila El Baghdadi^28^, Sara Espinosa-Padilla^29^, Jacques Fellay^30^, Carlos Flores^31^, José Luis Franco^7^, Antoine Froidure^32^, Peter K. Gregersen^33^, Filomeen Haerynck^34^, David Hagin^35^, Rabih Halwani^36^, Lennart Hammarström^37^, James R. Heath^38^, Sarah E. Henrickson^39^, Elena W.Y. Hsieh^40^, Eystein S. Husebye^41^, Kohsuke Imai^42^, Yuval Itan^43^, Erich D. Jarvis^44^, Timokratis Karamitros^45^, Kai Kisand^46^, Cheng-Lung Ku^47^, Yu-Lung Lau^48^, Yun Ling^49^, Carrie L. Lucas^50^, Tom Maniatis^51^, Davood Mansouri^52^, László Maródi^53^, Isabelle Meyts^54^, Joshua D. Milner^55^, Kristina Mironska^56^, Trine H. Mogensen^57^, Tomohiro Morio^58^, Lisa F.P. Ng^59^, Luigi D. Notarangelo^60^, Antonio Novelli^61^, Giuseppe Novelli^62^, Cliona O’Farrelly^63^, Satoshi Okada^64^, Tayfun Ozcelik^65^, Qiang Pan-Hammarström^37^, Rebeca Perez de Diego^66^, Anna M. Planas^67^, Carolina Prando^68^, Aurora Pujol^69^, Lluis Quintana-Murci^70^, Laurent Renia^59^, Igor Resnick^71^, Carlos Rodríguez-Gallego^72^, Vanessa Sancho-Shimizu^73^, Anna Sediva^74^, Mikko R.J. Seppänen^75^, Mohammed Shahrooei^76^, Anna Shcherbina^77^, Ondrej Slaby^78^, Andrew L. Snow^79^, Pere Soler-Palacín^80^, András N. Spaan^81^, Ivan Tancevski^82^, Stuart G. Tangye^83^, Ahmad Abou Tayoun^84^, Sathishkumar Ramaswamy^84^, Stuart E Turvey^85^, K M Furkan Uddin^86^, Mohammed J. Uddin^87^, Diederik van de Beek^88^, Donald C. Vinh^89^, Horst von Bernuth^90^, Mayana Zatz^91^, Pawel Zawadzki^92^, Helen C. Su^60^, Jean-Laurent Casanova^93^

^1^INSERM U1163, University of Paris, Imagine Institute, Paris, France. ^2^San Raffaele Telethon Institute for Gene Therapy, IRCCS Ospedale San Raffaele, and Vita Salute San Raffaele University, Milan, Italy. ^3^Immunology Research Laboratory, Department of Pediatrics, College of Medicine and King Saud University Medical City, King Saud University, Riyadh, Saudi Arabia. ^4^Dasman Diabetes Institute, Department of Genetics and Bioinformatics, Dasman, Kuwait. ^5^Diabetes Center, University of California San Francisco, San Francisco, CA, USA. ^6^Laboratory of Immunobiology, Center for Clinical, Experimental Surgery and Translational Research, Biomedical Research Foundation of the Academy of Athens, Athens, Greece. ^7^Group of Primary Immunodeficiencies, University of Antioquia UDEA, Medellin, Colombia. ^8^The Genetics Institute, Tel Aviv Sourasky Medical Center and Sackler Faculty of Medicine, Tel Aviv University, Tel Aviv, Israel. ^9^Pediatric Nephrology, Rheumatology, Dermatology, HFME, Hospices Civils de Lyon, National Referee Centre RAISE, and INSERM U1111, Université de Lyon, Lyon, France. ^10^Department of Pediatrics, British Columbia Children’s Hospital, The University of British Columbia, Vancouver, BC, Canada ^11^Icahn School of Medicine at Mount Sinai, New York, NY, USA. ^12^Helix, San Mateo, CA, USA. ^13^Shupyk National Medical Academy for Postgraduate Education, Kiev, Ukraine. ^14^Clinical Immunology Unit, Department of Pediatric Infectious Disease, CHU Ibn Rushd and LICIA, Laboratoire d’Immunologie Clinique, Inflammation et Allergie, Faculty of Medicine and Pharmacy, Hassan II University, Casablanca, Morocco. ^15^SciLifeLab, Department Of Women’s and Children’s Health, Karolinska Institutet, Stockholm, Sweden ^16^Department of Medicine, Center for Hematology and Regenerative Medicine, Karolinska Institutet, Stockholm, Sweden. ^17^Stanford University, Stanford, CA, USA. ^18^Division of Immunology, Allergy, and Rheumatology, Department of Pediatrics and the Department of Microbiology, Immunology, and Molecular Genetics, University of California, Los Angeles, CA, USA. ^19^Clinical Genomics, IRCCS San Raffaele Scientific Institute and Vita-Salute San Raffaele University, Milan, Italy ^20^Department of Pediatrics and Children’s Healthcare of Atlanta, Emory University, Atlanta, GA, USA. ^21^Murdoch Children’s Research Institute and Department of Paediatrics, University of Melbourne, Australia ^22^Department of Immunology, Institute of Biomedical Sciences, University of São Paulo, São Paulo, Brazil. ^23^de Duve Institute and Ludwig Cancer Research, Brussels, Belgium ^24^Washington University School of Medicine, St. Louis, MO, USA. ^25^Department of Anatomy, Physiology & Genetics, Uniformed Services University of the Health Sciences, Bethesda, MD, USA. ^26^Bai Jerbai Wadia Hospital for Children, Mumbai, India. ^27^School of Medicine and Public Health, University of Wisconsin, Madison, WI, USA. ^28^Genetics Unit, Military Hospital Mohamed V, Rabat, Morocco. ^29^Instituto Nacional de Pediatria (National Institute of Pediatrics), Mexico City, Mexico. ^30^School of Life Sciences, Ecole Polytechnique Fédérale de Lausanne, Lausanne, Switzerland; Precision Medicine Unit, Lausanne University Hospital and University of Lausanne, Lausanne, Switzerland. ^31^Genomics Division, Instituto Tecnológico y de Energías Renovables (ITER), Santa Cruz de Tenerife, Spain; Research Unit, Hospital Universitario N.S. de Candelaria, Santa Cruz de Tenerife, Spain; Instituto de Tecnologías Biomédicas (ITB), Universidad de La Laguna, San Cristóbal de La Laguna, Spain; CIBER de Enfermedades Respiratorias, Instituto de Salud Carlos III, Madrid, Spain. ^32^Pulmonology Department, Cliniques Universitaires Saint-Luc ; Institut de Recherche Expérimentale et Clinique (IREC), Université Catholique de Louvain, Brussels, Belgium. ^33^Feinstein Institute for Medical Research, Northwell Health USA, Manhasset, NY, USA. ^34^Department of Paediatric Immunology and Pulmonology, Centre for Primary Immunodeficiency Ghent (CPIG), PID Research Laboratory, Jeffrey Modell Diagnosis and Research Centre, Ghent University Hospital, Ghent, Belgium. ^35^The Genetics Institute Tel Aviv Sourasky Medical Center, Tel Aviv, Israel. ^36^Sharjah Institute of Medical Research, College of Medicine, University of Sharjah, Sharjah, United Arab Emirates. ^37^Department of Biosciences and Nutrition, Karolinska Institutet, Stockholm, Sweden. ^38^Institute for Systems Biology, Seattle, WA, USA. ^39^Department of Pediatrics, Division of Allergy Immunology, Children’s Hospital of Philadelphia, Philadelphia, PA, USA; Department of Microbiology, Perelman School of Medicine, University of Pennsylvania, Philadelphia, PA, USA ^40^Departments of Pediatrics, Immunology and Microbiology, University of Colorado, School of Medicine, Aurora, Colorado, USA ^41^ Department of Clinical Science and K.G. Jebsen Center for Auoimmune Diseases, University of Bergen, Bergen, Norway. ^42^Department of Community Pediatrics, Perinatal and Maternal Medicine, Tokyo Medical and Dental University (TMDU) ^43^Institute for Personalized Medicine, Icahn School of Medicine at Mount Sinai, New York, NY, USA; Department of Genetics and Genomic Sciences, Icahn School of Medicine at Mount Sinai, New York, NY, USA. ^44^Laboratory of Neurogenetics of Language and Howard Hughes Medical Institute, The Rockefeller University, New York, NY, USA. ^45^Bioinformatics and Applied Genomics Unit, Hellenic Pasteur Institute, Athens, Greece ^46^Molecular Pathology, Department of Biomedicine, Institute of Biomedicine and Translational Medicine, University of Tartu, Tartu Estonia. ^47^Chang Gung University, Taoyuan County, Taiwan. ^48^Department of Paediatrics & Adolescent Medicine, The University of Hong Kong, Hong Kong, China. ^49^Shanghai Public Health Clinical Center, Fudan University, Shanghai, China. ^50^Department of Immunobiology, Yale University School of Medicine, New Haven, CT, USA. ^51^Columbia University Zuckerman Institute, New York, NY ^52^Department of Clinical Immunology and Infectious Diseases, National Research Institute of Tuberculosis and Lung Diseases, The Clinical Tuberculosis and Epidemiology Research Center, National Research Institute of Tuberculosis and Lung Diseases (NRITLD), Masih Daneshvari Hospital, Shahid Beheshti University of Medical Sciences, Tehran, Iran. ^53^Primary Immunodeficiency Clinical Unit and Laboratory, Department of Dermatology, Venereology and Dermatooncology, Semmelweis University, Budapest, Hungary. ^54^Department of Pediatrics, University Hospitals Leuven; KU Leuven, Department of Microbiology, Immunology and Transplantation; Laboratory for Inborn Errors of Immunity, KU Leuven, Leuven, Belgium. ^55^Department of Pediatrics, Columbia University Irving Medical Center, New York, NY, USA. ^56^University Clinic for Children’s Diseases, Department of Pediatric Immunology, Medical Faculty, University “St.Cyril and Methodij” Skopje, North Macedonia. ^57^Department of Biomedicine, Aarhus University, Aarhus, Denmark ^58^Tokyo Medical & Dental University Hospital, Tokyo, Japan. ^59^A*STAR Infectious Disease Labs, Agency for Science, Technology and Research, Singapore; Lee Kong Chian School of Medicine, Nanyang Technology University, Singapore. ^60^National Institute of Allergy and Infectious Diseases, National Institutes of Health, Bethesda, MD, USA. ^61^Laboratory of Medical Genetics, IRCCS Bambino Gesù Children’s Hospital, Rome, Italy. ^62^Department of Biomedicine and Prevention, Tor Vergata University of Rome, Rome, Italy. ^63^Comparative Immunology Group, School of Biochemistry and Immunology, Trinity Biomedical Sciences Institute, Trinity College Dublin, Ireland. ^64^Department of Pediatrics, Graduate School of Biomedical and Health Sciences, Hiroshima University, Hiroshima, Japan. ^65^Department of Molecular Biology and Genetics, Bilkent University, Bilkent, Ankara, Turkey. ^66^Laboratory of Immunogenetics of Human Diseases, Innate Immunity Group, IdiPAZ Institute for Health Research, La Paz Hospital, Madrid, Spain. ^67^IIBB-CSIC, IDIBAPS, Barcelona, Spain. ^68^Faculdades Pequeno Príncipe, Instituto de Pesquisa Pelé Pequeno Príncipe, Curitiba, Brazil. ^69^Neurometabolic Diseases Laboratory, Bellvitge Biomedical Research Institute (IDIBELL), L’Hospitalet de Llobregat, Barcelona, Spain; Catalan Institution of Research and Advanced Studies (ICREA), Barcelona, Spain; Center for Biomedical Research on Rare Diseases (CIBERER), ISCIII, Barcelona, Spain. ^70^Human Evolutionary Genetics Unit, CNRS U2000, Institut Pasteur, Paris, France; Human Genomics and Evolution, Collège de France, Paris, France. ^71^University Hospital St. Marina, Varna, Bulgaria. ^72^Department of Immunology, University Hospital of Gran Canaria Dr. Negrín, Canarian Health System, Las Palmas de Gran Canaria, Spain; Department of Clinical Sciences, University Fernando Pessoa Canarias, Las Palmas de Gran Canaria, Spain ^73^Department of Paediatric Infectious Diseases and Virology, Imperial College London, London, UK; Centre for Paediatrics and Child Health, Faculty of Medicine, Imperial College London, London, UK. ^74^Department of Immunology, Second Faculty of Medicine Charles University, V Uvalu, University Hospital in Motol, Prague, Czech Republic. ^75^Adult Immunodeficiency Unit, Infectious Diseases, Inflammation Center, University of Helsinki and Helsinki University Hospital, Helsinki, Finland; Rare Diseases Center and Pediatric Research Center, Children’s Hospital, University of Helsinki and Helsinki University Hospital, Helsinki, Finland ^76^Saeed Pathobiology and Genetics Lab, Tehran, Iran; Department of Microbiology and Immunology, Clinical and Diagnostic Immunology, KU Leuven, Leuven, Belgium. ^77^Department of Immunology, Dmitry Rogachev National Medical Research Center of Pediatric Hematology, Oncology and Immunology, Moscow, Russia. ^78^Central European Institute of Technology & Department of Biology, Faculty of Medicine, Masaryk University, Brno, Czech Republic. ^79^Department of Pharmacology & Molecular Therapeutics, Uniformed Services University of the Health Sciences, Bethesda, MD, USA. ^80^Pediatric Infectious Diseases and Immunodeficiencies Unit, Vall d’Hebron Barcelona Hospital Campus, Barcelona, Spain. ^81^St. Giles Laboratory of Human Genetics of Infectious Diseases, Rockefeller Branch, The Rockefeller University, New York, NY, USA.; Department of Medical Microbiology, University Medical Center Utrecht, Utrecht, Netherlands ^82^Department of Internal Medicine II, Medical University of Innsbruck, Innsbruck, Austria. ^83^Garvan Institute of Medical Research, Darlinghurst, NSW, Australia; St Vincent’s Clinical School, Faculty of Medicine, UNSW Sydney, NSW, Australia. ^84^Al Jalila Children’s Hospital, Dubai, UAE ^85^BC Children’s Hospital, The University of British Columbia, Vancouver, Canada ^86^Centre for Precision Therapeutics, Genetic and Genomic Medicine Centre, NeuroGen Children Healthcare, Dhaka, Bangladesh; Holy Family Red Crescent Medical College, Dhaka, Bangladesh ^87^College of Medicine, Mohammed Bin Rashid University of Medicine and Health Sciences, Dubai, UAE; Cellular Intelligence (Ci) Lab, GenomeArc Inc., Toronto, ON, Canada ^88^Department of Neurology, Amsterdam Neuroscience, Amsterdam University Medical Center, University of Amsterdam, Amsterdam, The Netherlands. ^89^Department of Medicine, Division of Infectious Diseases, McGill University Health Centre, Montréal, Québec, Canada; Infectious Disease Susceptibility Program, Research Institute, McGill University Health Centre, Montréal, Québec, Canada. ^90^Department of Pediatric Pneumology, Immunology and Intensive Care, Charité Universitätsmedizin, Berlin University Hospital Center, Berlin, Germany; Labor Berlin GmbH, Department of Immunology, Berlin, Germany; Berlin Institutes of Health (BIH), Berlin-Brandenburg Center for Regenerative Therapies, Berlin, Germany. ^91^Biosciences Institute, University of São Paulo, São Paulo, Brazil. ^92^Molecular Biophysics Division, Faculty of Physics, A. Mickiewicz University, Poznań, Poland. ^93^The Rockefeller University & Howard Hughes Medical Institute, New York, NY, USA; Necker Hospital for Sick Children & INSERM, Paris, France.

### CONSTANCES cohort

Rachel Nadif^1^, Marcel Goldberg^2^, Anna Ozguler^2^, Joseph Henny^2^, Sylvie Lemonnier^2^, Mireille Coeuret-Pellicer^3^, Stéphane Le Got^2^, Marie Zins^2^

^1^Université de Paris-Saclay, UVSQ, Université Paris-Sud, Inserm, Equipe d’Epidémiologie Respiratoire Intégrative, Inserm CESP, Villejuif, France. ^2^Université de Paris, Université Paris-Saclay, UVSQ, Inserm UMS11, Villejuif, France. ^3^Inserm U011 Constances cohort, Villejuif, France.

### 3C-Dijon Study

Christophe Tzourio^1^, Stéphanie Debette^2^, Carole Dufouil^1^, Aïcha Soumaré^1^, Morgane Lachaize^2^, Nathalie Fievet^3^, Amandine Flaig^3^

^1^University of Bordeaux; Bordeaux Population Health Center, INSERM U1219, Bordeaux, France. ^2^University of Bordeaux; Bordeaux Population Health Center, INSERM U1219; Bordeaux University Hospital, Department of Neurology, Institute of Neurodegenerative Diseases, Bordeaux, France. ^3^Laboratoire d’Analyses Génomiques - Centre de Ressources Biologiques; Institut Pasteur de Lille, Lille, France.

### Cerba Health-Care

Fernando Martin^1^

^1^Cerba Health Care, Issy-les-Moulineaux, France.

### Etablissement du Sang study group

Brigitte Bonneaudeau^1^, Dorothée Cannet^2^, Pierre Gallian^3^, Michel Jeanne^4^, Magali Perroquin^4^, Hind Hamzeh-Cognasse^5, 6^

^1^La Plaine St-Denis, France. ^2^Dijon, France. ^3^Marseille, France. ^4^Bordeaux, France. ^5^Saint-Etienne, France. ^6^ SAINBIOSE, INSERM, U1059, University of Lyon, Université Jean-Monnet-Saint-Etienne.
