# Supplementary material for: Autoantibodies neutralizing type I IFNs are present in ~ 4% of uninfected individuals over 70 years and account for ~ 20% of COVID-19 deaths
Source: Sci Immunol. Author manuscript; Available in PMC 2022 Feb 19. (PMC8521484; doi:10.1126/sciimmunol.abl4340)
Supplement: Supplementary file 1 [file NIHMS1744468-supplement-Supplemental_Material.pdf]

Science
AAAS

Supplementary Materials for

Autoantibodies neutralizing type I IFNs are present in ~ 4% of uninfected individuals over 70 years and account for ~ 20% of COVID-19 deaths

Paul Bastard[1-3,@], Adrian Gervais[1,2,&], Tom Le Voyer[1,2,&], Jérémie Rosain[1,2,&], Quentin Philippot[1,2,&], Jérémy Manry[1,2,#], Eleftherios Michailidis[4,#], Hans-Heinrich Hoffmann[4,#], Shohei Eto[5, £], Marina Garcia-Prat[6,£], Lucy Bizien[1,2,£], Alba Parra-Martínez[6,£], Rui Yang[3,£], Liis Haljasmägi[7,£], Mélanie Migaud[1,2,£], Karita Särekannu[7,£], Julia Maslovskaja[7,£], Nicolas de Prost[8,9], Yacine Tandjaoui-Lambiotte[10], Charles-Edouard Luyt[11,12], Blanca Amador-Borrero[13], Alexandre Gaudet[14-18], Julien Poissy[14-18], Pascal Morel[19,20], Pascale Richard[19], Fabrice Cognasse[21,22], Jesus Troya[23], Sophie Trouillet-Assant[24], Alexandre Belot[25,26], Kahina Saker[25], Pierre Garçon[27], Jacques G. Rivière[6], Jean-Christophe Lagier[28,29], Stéphanie Gentile[30,31], Lindsey B. Rosen[32], Elana Shaw[32], Tomohiro Morio[33], Junko Tanaka[34], David Dalmau[35], Pierre-Louis Tharaux[36], Damien Sene[13], Alain Stepanian[37], Bruno Megarbane[38], Vasiliki Triantafyllia[39], Arnaud Fekkar[1,40], James R. Heath[41], José Luis Franco[42], Juan-Manuel Anaya[43], Jordi Solé-Violán[44,45], Luisa Imberti[46], Andrea Biondi[47], Paolo Bonfanti[48], Riccardo Castagnoli[32,49], Ottavia M. Delmonte[32], Yu Zhang[32,50], Andrew L. Snow[51], Steven M. Holland[32], Catherine Biggs[52], Marcela Moncada-Vélez[3], Andrés Augusto Arias[3,53,54], Lazaro Lorenzo[1,2], Soraya Boucherit[1,2], Boubacar Coulibaly[1,2], Dany Anglicheau[55], Anna M. Planas[56,57], Filomeen Haerynck[58], Sotirija Duvlis[59,60], Robert L. Nussbaum[61], Tayfun Ozcelik[62], Sevgi Keles[63], Ahmed A. Bousfiha[64], Jalila El Bakkouri[64], Carolina Ramirez-Santana[42,43], Stéphane Paul[65], Qiang Pan-Hammarström[66], Lennart Hammarström[66], Annabelle Dupont[67], Alina Kurolap[68], Christine N. Metz[69], Alessandro Aiuti[70], Giorgio Casari[70], Vito Lampasona[71], Fabio Ciceri[72], Lucila A. Barreiros[73], Elena Dominguez-Garrido[74], Mateus Vidigal[75], Mayana Zatz[75], Diederik van de Beek[76], Sabina Sahanic[77], Ivan Tancevski[77], Yurii Stepanovskyy[78], Oksana Boyarchuk[79], Yoko Nukui[80], Miyuki Tsumura[5], Loreto Vidaur[81,82], Stuart G. Tangye[83], Sonia Burrel[84], Darragh Duffy[85], Lluis Quintana-Murci[86,87], Adam Klocperk[88], Nelli Y. Kann[89], Anna Shcherbina[89], Yu-Lung Lau[90], Daniel Leung[90], Matthieu Coulongeat[91], Julien Marlet[92,93], Rutger Koning[76], Luis Felipe Reyes[94,95], Angélique Chauvineau-Grenier[96], Fabienne Venet[97-99], Guillaume Monneret[97,99], Michel C. Nussenzweig[100,101], Romain Arrestier[8,9], Idris Boudhabhay[55], Hagit Baris-Feldman[68,102], David Hagin[102,103], Joost Wauters[104], Isabelle Meyts[105,106], Adam H. Dyer[107], Sean P. Kennelly[107], Nollaig M. Bourke[108], Rabih Halwani[109], Narjes Saheb Sharif-Askari[109], Karim Dorgham[110], Jérome Sallette[111], Souad Mehlal Sedkaoui[111], Suzan AlKhater[112,113], Raúl Rigo-Bonnin[114], Francisco Morandeira[115], Lucie Roussel[116,117], Donald C. Vinh[116,117], Sisse Rye Ostrowski[118], Antonio Condino-Neto[73], Carolina Prando[119], Anastasiia Bonradenko[78], András N. Spaan[3,120], Laurent Gilardin[121,122], Jacques Fellay[123-125], Stanislas Lyonnet[126], Kaya Bilguvar[127-130], Richard P. Lifton[127,131,132], Shrikant Mane[128], HGID Lab[§], COVID Clinicians[§], COVID-STORM Clinicians[§], NIAID Immune Response to COVID Group[§], NH-COVAIR Study Group[§],

Danish CHGE[§], Danish Blood Donor Study[§], St. James's Hospital, SARS CoV2 Interest group[§], French COVID Cohort Study Group[§], Imagine COVID-Group[§], The Milieu Intérieur Consortium[§], CoV-Contact Cohort[§], Amsterdam UMC Covid-19 Biobank Investigators[§], COVID Human Genetic Effort[§], CONSTANCES cohort[§], 3C-Dijon Study[§], Cerba Health-Care[§], Etablissement du Sang study group[§], Mark S. Anderson[133], Bertrand Boisson[1-3], Vivien Béziat[1,2], Shen-Ying Zhang[1-3], Evangelos Vandreakos[134,†], Olivier Hermine[2,135,†], Aurora Pujol[136,†], Pärt Peterson[7,†], Trine H. Mogensen[137,138,†], Lee Rowen[41,†], James Mond[139,†], Stéphanie Debette[140,141,†], Xavier de Lamballerie[142,†], Xavier Duval[143-146,†], France Mentré[143-145,†], Marie Zins[147,†], Pere Soler-Palacin[6,†], Roger Colobran[148,†], Guy Gorochov[110,149,†], Xavier Solanich[150,†], Sophie Susen[67,†], Javier Martinez-Picado[151-153,†], Didier Raoult[28,29,†], Marc Vasse[154,†], Peter K. Gregersen[69,†], Lorenzo Piemonti[71,†], Carlos Rodríguez-Gallego[155,156†], Luigi D. Notarangelo[32, ‡], Helen C. Su [32,157, ‡], Kai Kisand[7, ‡], Satoshi Okada[5, ‡], Anne Puel[1-3,‡], Emmanuelle Jouanguy[1-3,‡], Charles M. Rice [4,‡], Pierre Tiberghien[19,20,‡], Qian Zhang[1-3,‡], Aurélie Cobat[1-3‡], Laurent Abel [1-3,*] and Jean-Laurent Casanova [1-3,101,*,@]

[&]These authors contributed equally to this work.
[#]These authors contributed equally to this work.
[£]These authors contributed equally to this work.
[†]These authors contributed equally to this work.
[‡]These authors contributed equally to this work.
[*]These authors contributed equally to this work.
[§]All collaborators and their affiliations appear at the end of this paper.
[@]corresponding authors: jean-laurent.casanova@rockefeller.edu; paul.bastard@institutimagine.org

This PDF file includes:

Supplementary materials and methods
Legends to supplementary tables (S1 to S6)
Legends to supplementary figures
Supplementary figures (S1 to S6)

Supplementary materials and methods

Subjects and samples

We enrolled, from 38 countries across all continents, 3,595 patients with proven critical COVID-19, 623 with severe COVID-19 and 1,639 asymptomatic or paucisymptomatic individuals with proven COVID-19, and 34,159 healthy controls in this study. All subjects were recruited according to protocols approved by local institutional review boards (IRBs). For patients enrolled in the Italian cohort, ethics approval was obtained from the University of Milano-Bicocca School of Medicine, San Gerardo Hospital, Monza – Ethics Committee of the National Institute of Infectious Diseases Lazzaro Spallanzani (84/2020) (Italy), the IRB of Fondazione IRCCS Policlinico San Matteo, Pavia (Italy), and the Comitato Etico Provinciale (NP 4000 – Studio CORONAlab), Fondazione IRCCS Ca' Granda Ospedale Maggiore policlinico of Milan - Comitato Etico Milano Area 2 (369_2020bis – COVID HGE study). STORM-Health care workers were enrolled in the STudio OsseRvazionale sullo screening dei lavoratori ospedalieri per COVID- 19 (STORM-HCW) study, which was approved by the local IRB on June 18, 2020. For the COV-Contact study, ethics approval was obtained from the CPP IDF VI (ID RCB: 2020-A00280- 39). For patients were enrolled in the French COVID cohort (clinicaltrials.gov NCT04262921), ethics approval was obtained from the CPP IDF VI (ID RCB: 2020-A00256-33). Some contact subjects were enrolled in the Cov-Contact cohort (clinicaltrials.gov NCT04259892). Anonymized samples were studied at the NIAID under non-human subject research conditions; no additional IRB consent was required at the NIH. Protocols in San Raffaele were approved by San Raffaele Hospital Ethical Committee. Samples were obtained from the Milieur Intérieur Cohort, which was approved by the Comité de Protection des Personnes – Ouest 6 (Committee for the protection of persons) on June 13, 2012 and by the French Agence nationale de sécurité du médicament (ANSM) on June 22, 2012. The Milieur Intérieur Cohort study is sponsored by Institut Pasteur (Pasteur ID-RCB Number: 2012-A00238-35), and was conducted as a single-center interventional study without an investigational product. The original protocol was registered under ClinicalTrials.gov (study# NCT01699893). The samples and data used in this study were formally established as the Milieu Interieur biocollection (NCT03905993), with approval from the Comité de Protection des Personnes – Sud Méditerranée and the Commission nationale de l'informatique et des libertés (CNIL), obtained on April 11, 2018. The patients of the Danish Covid-19 cohort were enrolled prospectively and ethics approval was obtained from the Danish National Committee on Health Research Ethics (#1-10-72-80-20). THM received financial support from The Novo Nordisk Foundation (NNF21OC0067157). The Constances Cohort Study is an "Infrastructure nationale en Biologie et Santé" and benefits from a grant from ANR (ANR-11-INBS-0002). The content of the manuscript is the sole responsibility of the authors and does not necessarily represent the official views of any of the funding sources. The patients of the Amsterdam UMC Covid-19 cohort were enrolled prospectively at the Amsterdam UMC. Ethics approval was obtained from the Amsterdam UMC Biobank Committee (202_065#A202029). For patients enrolled at University Hospitals Leuven (Belgium), ethics approval was obtained for the CONTAGIOUS study (S63881, clinicaltrials.gov NCT04327570). Patients were enrolled in the Danish CHGE group after permission from the National Committee on Health Research Ethics (1-10-72-80-20). Blood donors with previous SARS-CoV-2 infection and matching controls were collected from The Danish Blood Donor Study (DBDS) biobank. The DBDS has been approved by the Central Denmark and Zealand Committees

on Health Research Ethics (SJ-740 and 1-10-72-95-13) and the data processing was approved by the Capital Region (P-2019-99). The 3C-Dijon is population-based based cohort of individuals aged 65 years and older recruited from the electoral rolls of the city of Dijon. All protocols conformed to local ethics recommendations and informed consent was obtained when required.

Legends to supplementary tables:

Table S1: Total numbers of critical patients, severe patients, asympto- or pauci-symptomatic controls tested for neutralizing auto-Abs against type I IFNs. All is shown by gender, age, type of type I IFN auto-Ab and dose of type I IFN neutralized.

Table S2: Risk, adjusted on age and sex, of having critical or severe disease for subjects carrying auto-Abs to each individual IFN and the different possible combinations, compared to the asymptomatic controls or to general population.

Table S3: Total numbers of critical patients, severe patients, asympto- or pauci-symptomatic controls tested for neutralizing auto-Abs against IFN-β. All is shown by gender, age, type of type I IFN auto-Ab and dose of type I IFN neutralized.

Table S4: Effect of age and sex on the presence of auto-Abs in individuals with critical or severe COVID-19, and in the general population.

Table S5: Age-stratified proportion of individuals from the general population tested for neutralizing auto-Abs against type I IFNs, by 10-year bins. All is shown by gender, age, type of type I IFN auto-Ab and dose of type I IFN neutralized.

Table S6: Age-stratified proportion of the general population tested for neutralizing auto-Abs against type I IFNs, by 5-year bins. All is shown by gender, age, type of type I IFN auto-Abs and dose of type I IFN neutralized.

Legends to supplementary figures

Fig. S1. Neutralizing auto-Abs against IFN-α2 and/or IFN-ω in patients with life-threatening COVID-19. (A) Gyros anti-IFN-α2 and anti-IFN-ω auto-Ab titers for various dilutions of plasma from a patient positive for auto-Abs against type I IFNs, representative of 3 independent patients with auto-Abs. **(B)** Plot of anti–IFN-α2 auto-Ab levels, as detected by ELISA, against the values obtained for the same samples with Gyros (*N* = 48 independent samples). A simple linear regression analysis was performed (black line). R^2 = 0.9197. R^2, coefficient of determination. **(C)** Plot of the capacity to neutralize IFN-α2, as determined in pSTAT1 assays on PBMCs against that determined in the luciferase assay. The stimulation index (stimulated over unstimulated) for plasma from each patient was normalized against that of healthy control plasma from the same experiment for the pSTAT1 assay and that of the mean induction of control plasma tested the same day for the luciferase assay. A simple linear regression analysis was performed (black line). R^2= 0.6036. RLA: Relative luciferase activity. **(D)** Plot of the capacity to neutralize IFN-ω, as determined with the pSTAT1 assay on PBMCs, against that determined in the luciferase assay. The stimulation index (stimulated over unstimulated) for plasma from each patient was normalized against that of healthy control plasma from the same experiment for the pSTAT1 assay, whereas luciferase activity was normalized against the *Renilla* luciferase activity of control plasma tested the same day. A simple linear regression analysis was performed (black line). R^2 = 0.6175. **(E)** Plot of anti–IFN-α2 auto-Ab levels, as determined by Gyros, against their neutralization capacity at 10 ng/mL in the luciferase assay. The luciferase activity for the plasma from each patient was normalized against the mean induction of control plasma tested the same day for the luciferase assay. The horizontal dotted line indicates neutralizing levels, defined as induction levels below 15% of the mean value for controls tested the same day. The vertical dotted lines represent Gyros auto-Ab levels. **(F)** Relative luciferase activity (RLA) (ISRE dual luciferase activity, with normalization against *Renilla* luciferase activity) after stimulation with various amounts of IFN-α2, in presence of plasma 1/10. **(G)** Relative luciferase activity (RLA) (ISRE dual luciferase activity, with normalization against *Renilla* luciferase activity) after stimulation with IFN-ω at a concentration of 10 ng/ml or 100 pg/mL, with various dilutions of plasma from a positive control (from 1/10 to $1/10^7$) neutralizing 10 ng/mL of type I IFNs (C+, 10 ng/mL), a patient neutralizing 100 pg/mL of type I IFNs but not 10 ng/mL (C+, 100 pg/mL), and a healthy control (HC). **(H)** Results for the neutralization of 10ng/mL IFN-α2 or IFN-ω in the presence of plasma 1/10 from the 4 patients with critical COVID-19 for which samples were available before their COVID-19 infection. Relative luciferase activity is shown (ISRE dual luciferase activity, with normalization against *Renilla* luciferase activity) after stimulation with 10ng/mL IFN-α2 or IFN-ω in the presence of plasma 1/10. RLA: relative luciferase activity. **(I)** Plot of anti-IFN-a2 (upper panel), or anti-IFN-ω (lower panel) auto-Ab levels assessed by Luminex, against their neutralization capacity, assessed by the pSTAT1 assays on PBMCs. The stimulation index (stimulated over unstimulated condition) for the plasma from each patient was normalized against that of healthy control plasma from the same experiment. **(J)** ELISA for IgA and IgM auto-Abs against IFN-α2 or IFN-ω in patients with critical COVID-19 (*N* = 12), and undetectable auto-Abs neutralizing IFN-α2 and/or -ω. **(K)** Neutralization of 10 ng/mL IFN-β in the presence of plasma 1/10 from patients with critical COVID-19 (*N* = 12), and undetectable auto-Abs neutralizing IFN-α2 and/or -ω.

Fig. S2. Enhanced SARS-CoV-2 replication, despite the presence of IFN-α2, in the presence of plasma from patients with auto-Abs neutralizing 100 pg/mL IFN-α2.
(A) IgG purification experiment in 1 healthy control, 1 APS-1 patient and 3 patients with critical COVID-19 capable of neutralizing 100 pg/mL of IFN-α2. RLA ratio is shown after stimulation with 100 pg/mL of IFN-α2, in presence of plasma only, the IgG depleted fraction of the plasma, or the IgG positive eluted fraction. **(B)** ELISA for auto-Abs against IFN-β in patients with life-threatening COVID-19 (*N* = 384) and mild/asymptomatic controls (*N* = 109).

Fig. S3. Higher prevalence of neutralizing auto-Abs against type I IFNs in elderly patients with critical COVID-19. (A) Graph showing the IFN-ω auto-Ab levels, assessed by Gyros, in patients with life-threatening COVID-19. Men or women are shown separately. The upper section of the Y-axis starts at 3%. **(B-S)** Proportion by decade of patients with critical COVID-19, and positive for neutralizing auto-Abs (in plasma 1/10) against **(B)** IFN-α2, at 10 ng/mL, for both sexes. **(C)** IFN-α2, at 10 ng/mL, for men and women. **(D)** IFN-α2 only, at 10 ng/mL, for both sexes. **(E)** IFN-α2 only, at 10 ng/mL, for men and women. **(F)** IFN-ω, at 10 ng/mL, for both sexes. **(G)** IFN-ω, at 10 ng/mL, for men and women. **(H)** IFN-ω only, at 10 ng/mL, for both sexes. **(I)** IFN-ω only, at 10 ng/mL, for men and women. **(J)** IFN-β, at 10 ng/mL, for both sexes. **(K)** IFN-β, at 10 ng/mL, for men and women. **(L)** IFN-α2, at 100 pg/mL, for both sexes. **(M)** IFN-α2, at 100 pg/mL, for both sexes. **(N)** IFN-α2 only, at 100 pg/mL, for both sexes. **(O)** IFN-α2 only, at 100 pg/mL, for men and women. **(P)** IFN-ω, at 100 pg/mL, for both sexes. **(Q)** IFN-ω, at 100 pg/mL, for men and women. **(R)** IFN-ω only, at 100 pg/mL, for both sexes. **(S)** IFN-ω only, at 100 pg/mL, for men and women. **(T-W)** Proportion by decade of patients with severe COVID-19, and positive for neutralizing auto-Abs (in plasma 1/10) against **(T)** IFN-α2 and/or IFN-ω, at 10 ng/mL, for both sexes. **(U)** IFN-α2 and/or IFN-ω, at 10 ng/mL, for men and women. **(V)** IFN-α2 and/or IFN-ω, at 10 ng/mL, for both sexes. **(W)** IFN-α2 and/or IFN-ω, at 100 pg/mL, for men and women.

Fig. S4. Higher prevalence of neutralizing auto-Abs against type I IFNs in patients who died of COVID-19. (A) Graph showing the IFN-ω auto-Ab levels, assessed by Gyros, in patients who died of COVID-19. Men or women are shown separately. **(B-K)** Proportion by decade of patients who died of COVID-19, and positive for neutralizing auto-Abs (in plasma 1/10) against **(B)** IFN-α2, at 10 ng/mL, for both sexes. **(C)** IFN-α2, at 10 ng/mL, for men and women. **(D)** IFN-ω, at 10 ng/mL, for both sexes. **(E)** IFN-ω, at 10 ng/mL, for men and women. **(F)** IFN-α2, at 100 pg/mL, for both sexes. **(G)** IFN-α2, at 100 pg/mL, for men and women. **(H)** IFN-ω, at 100 pg/mL, for both sexes. **(I)** IFN-ω, at 100 pg/mL, for men and women. **(J)** IFN-β, at 10 ng/mL, for both sexes. **(K)** IFN-β, at 10 ng/mL, for men and women.

Fig. S5. Neutralizing auto-Abs against IFN-α2 and/or IFN-ω at 10 ng/mL are more prevalent in the elderly, in the general population. (A) Graph showing the IFN-ω auto-Ab levels, assessed by Gyros, in individuals from the general population. Men or women are shown separately. **(B-I)** Proportion, binned into 5 year-intervals, of individuals from the general population, and positive for neutralizing auto-Abs (in plasma 1/10) against **(B)** IFN-α2, at 10 ng/mL, for both sexes. **(C)** IFN-α2, at 10 ng/mL, for men and women. **(D)** IFN-α2 only, at 10 ng/mL, for both sexes. **(E)** IFN-α2 only, at 10 ng/mL, for men and women. **(F)** IFN-ω, at 10 ng/mL, for both sexes. **(G)** IFN-ω, at 10 ng/mL, for men and women. **(H)** IFN-ω only, at 10 ng/mL, for both sexes.

(I) IFN-ω only, at 10 ng/mL, for men and women. **(J)** Type I IFN auto-Abs, assessed by LIPS (Luciferase based immunoprecipitation assay) in an independent Estonian cohort of 703 elderly individuals from the general population. **(K)** Type I IFN auto-Abs, assessed by ELISA in an independent Japanese cohort of 1002 individuals of all ages from the general population, including 376 elderly individuals.

Fig. S6. Neutralizing auto-Abs against IFN-α2 and/or IFN-ω at 100 pg/mL are more prevalent in the elderly, in the general population. Proportion, binned into 5 year-intervals, of individuals from the general population, and positive for neutralizing auto-Abs (in plasma 1/10) against **(A)** IFN-α2 only, at 100 pg/mL, for both sexes. **(B)** IFN-α2 only, at 100 pg/mL, for men and women. **(C)** IFN-ω only, at 100 pg/mL, for both sexes. **(D)** IFN-ω only, at 100 pg/mL, for men and women.

Figure S1

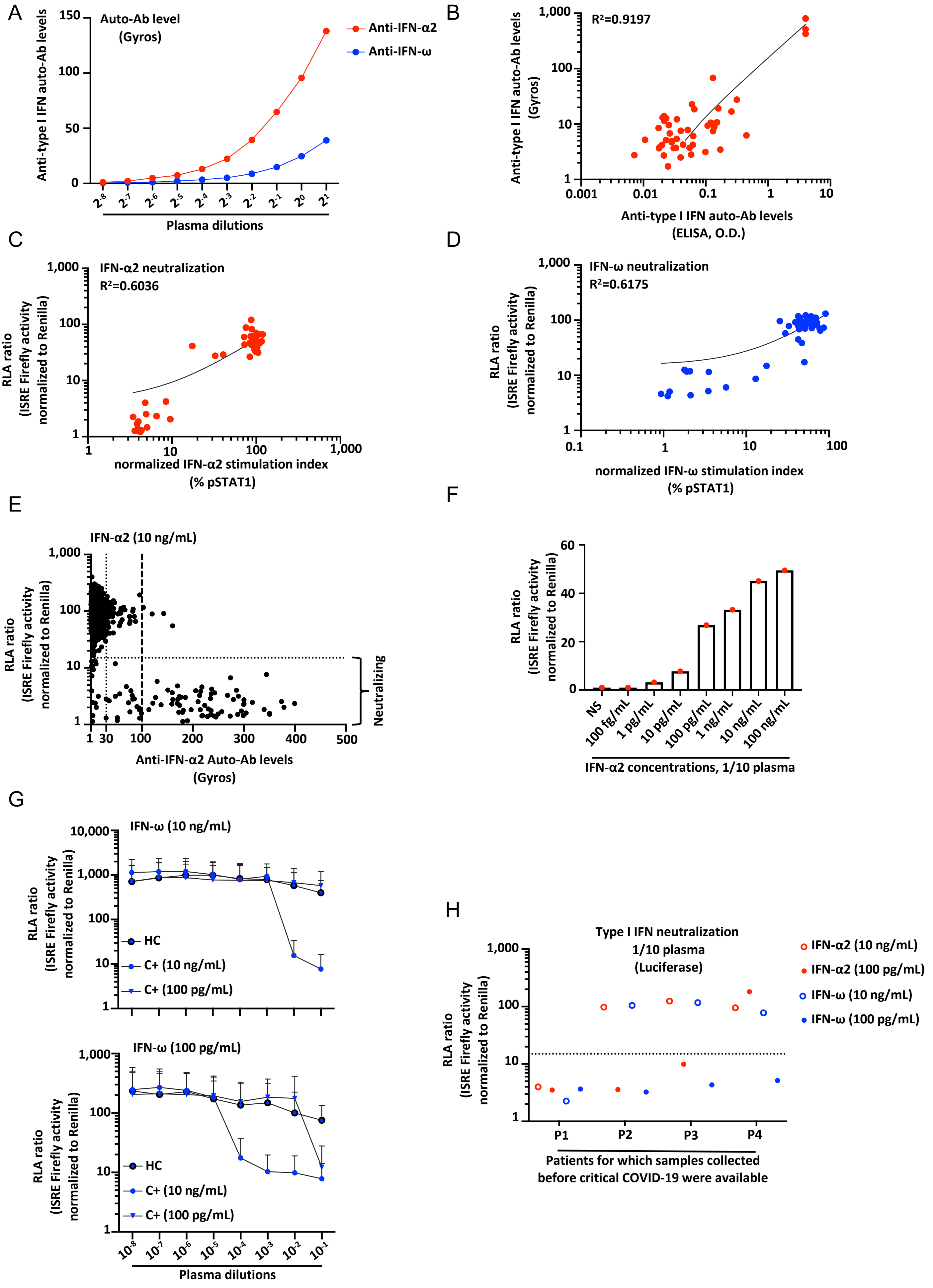
A
Auto-Ab level (Gyros)
Anti-IFN-α2
Anti-IFN-ω
Anti-type I IFN auto-Ab levels
Plasma dilutions
B
R²=0.9197
Anti-type I IFN auto-Ab levels (Gyros)
Anti-type I IFN auto-Ab levels (ELISA, O.D.)
C
IFN-α2 neutralization
R²=0.6036
RLA ratio (ISRE Firefly activity normalized to Renilla)
normalized IFN-α2 stimulation index (% pSTAT1)
D
IFN-ω neutralization
R²=0.6175
RLA ratio (ISRE Firefly activity normalized to Renilla)
normalized IFN-ω stimulation index (% pSTAT1)
E
IFN-α2 (10 ng/mL)
RLA ratio (ISRE Firefly activity normalized to Renilla)
Neutralizing
Anti-IFN-α2 Auto-Ab levels (Gyros)
F
RLA ratio (ISRE Firefly activity normalized to Renilla)
NS
100 fg/mL
1 pg/mL
10 pg/mL
100 pg/mL
1 ng/mL
10 ng/mL
100 ng/mL
IFN-α2 concentrations, 1/10 plasma
G
IFN-ω (10 ng/mL)
RLA ratio (ISRE Firefly activity normalized to Renilla)
HC
C+ (10 ng/mL)
C+ (100 pg/mL)
IFN-ω (100 pg/mL)
Plasma dilutions
H
Type I IFN neutralization
1/10 plasma
(Luciferase)
RLA ratio (ISRE Firefly activity normalized to Renilla)
IFN-α2 (10 ng/mL)
IFN-α2 (100 pg/mL)
IFN-ω (10 ng/mL)
IFN-ω (100 pg/mL)
P1
P2
P3
P4
Patients for which samples collected before critical COVID-19 were available

Figure S1

I

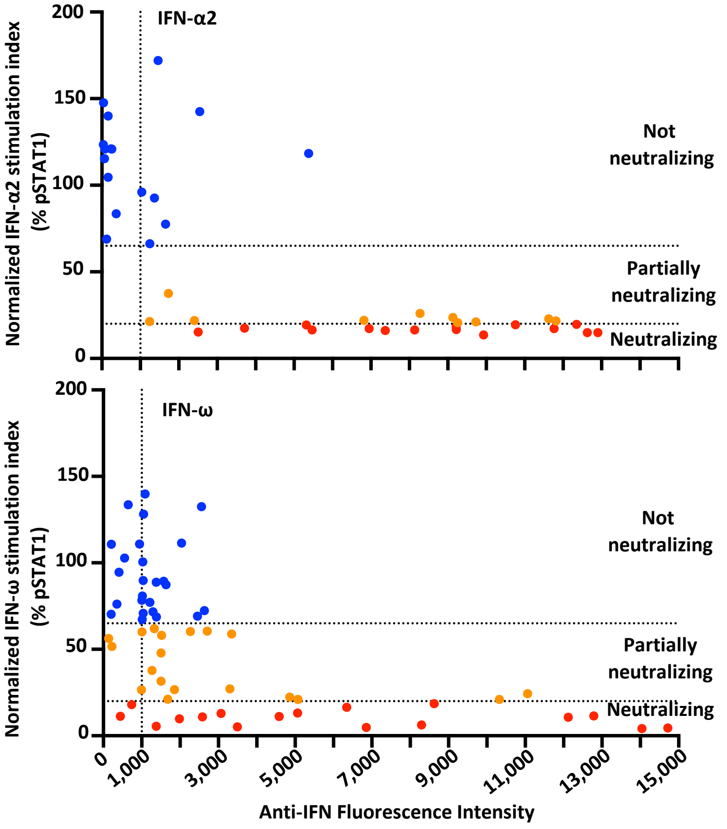

J

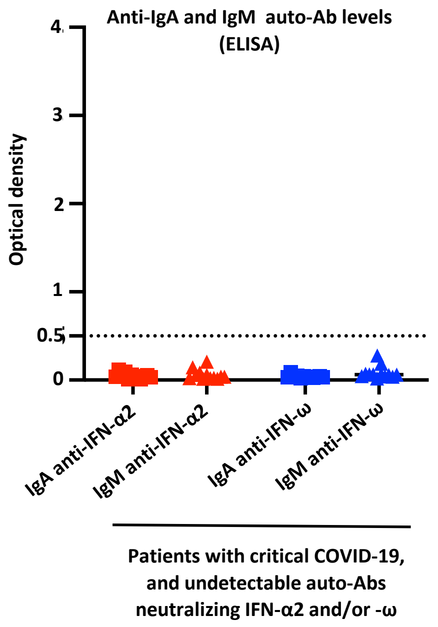

K

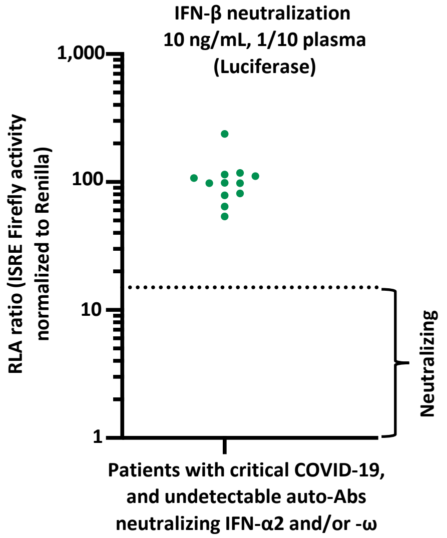

Figure S2

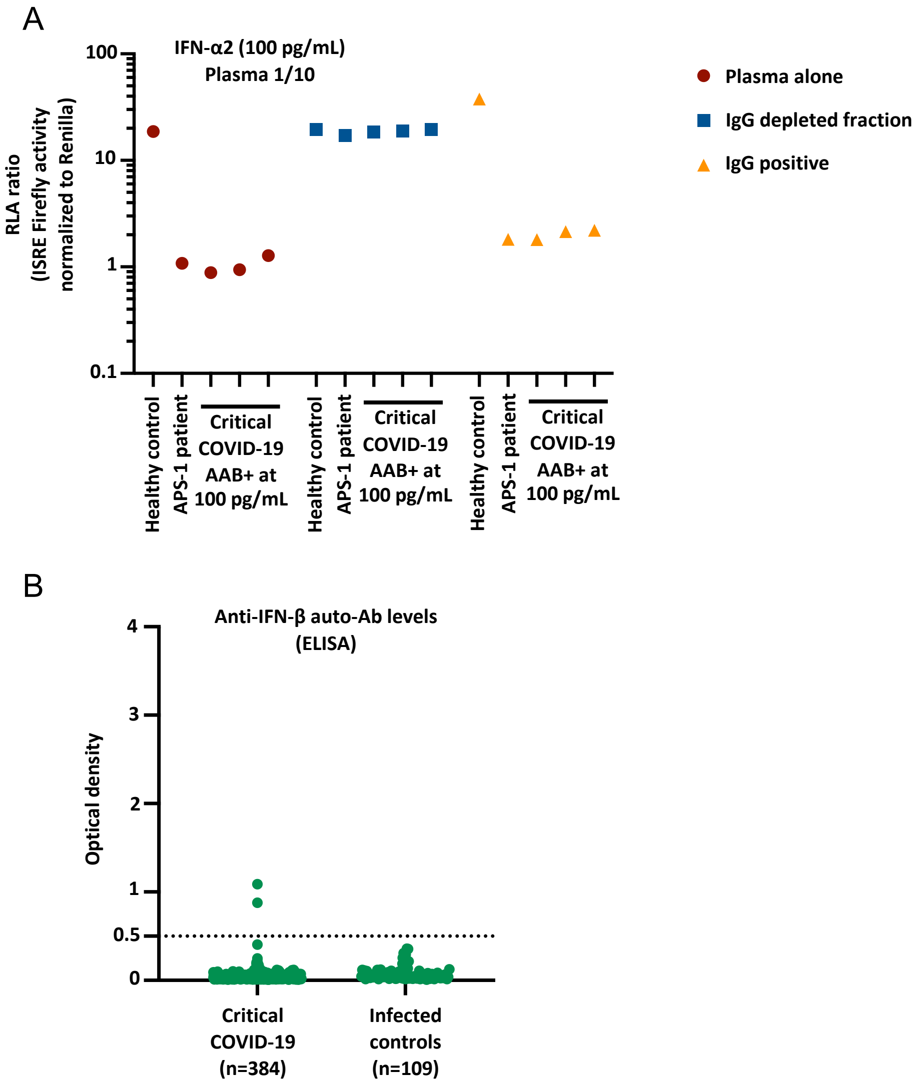
A
IFN-α2 (100 pg/mL)
Plasma 1/10
RLA ratio
(ISRE Firefly activity
normalized to Renilla)
100
10
1
0.1
Plasma alone
IgG depleted fraction
IgG positive
Healthy control
APS-1 patient
Critical
COVID-19
AAB+ at
100 pg/mL
Healthy control
APS-1 patient
Critical
COVID-19
AAB+ at
100 pg/mL
Healthy control
APS-1 patient
Critical
COVID-19
AAB+ at
100 pg/mL
B
Anti-IFN-β auto-Ab levels
(ELISA)
Optical density
4
3
2
1
0.5
0
Critical
COVID-19
(n=384)
Infected
controls
(n=109)

Figure S3

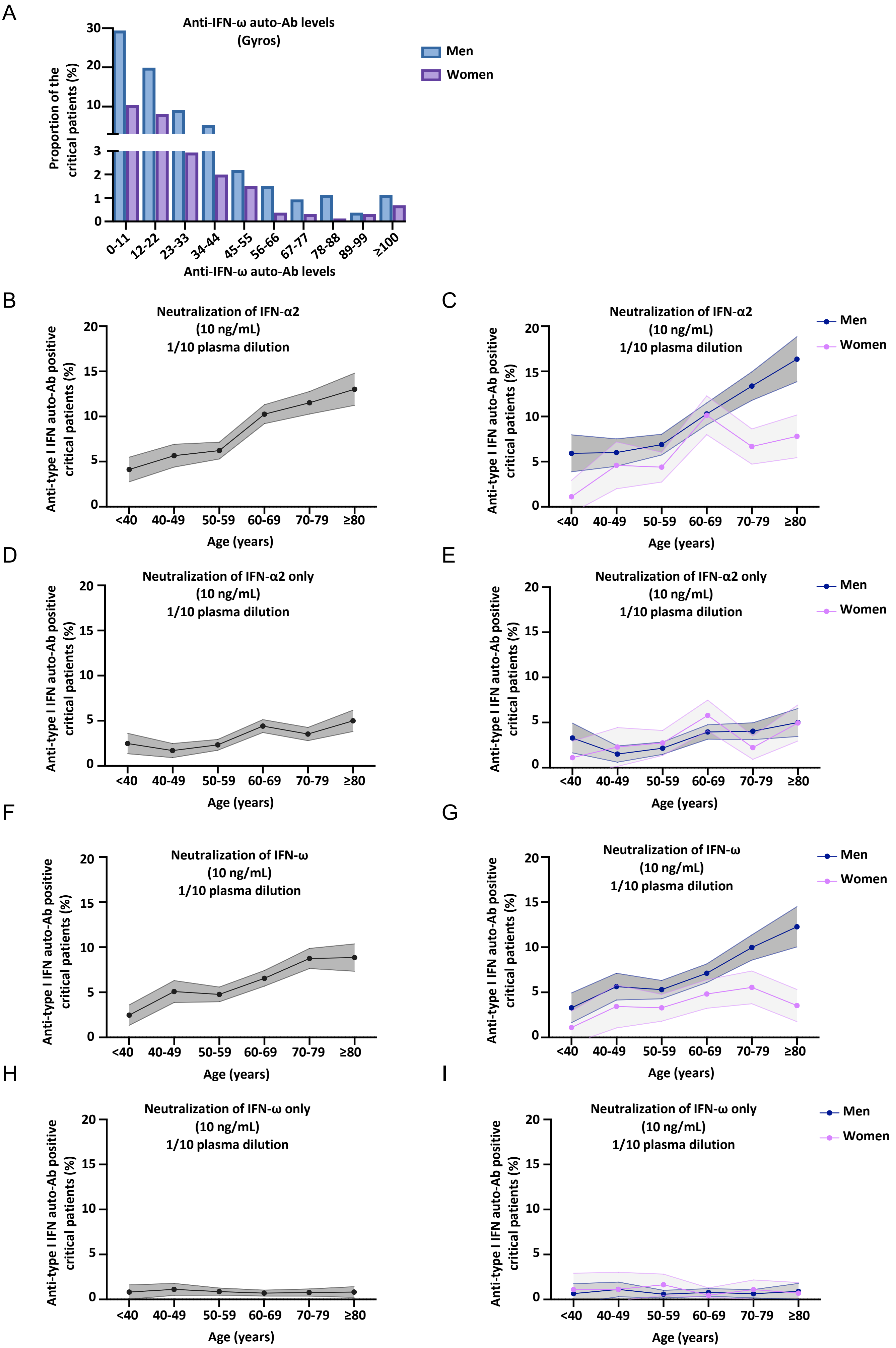
A
Anti-IFN-ω auto-Ab levels
(Gyros)
Men
Women
Proportion of the
critical patients (%)
30
20
10
3
2
1
0
0-11
12-22
23-33
34-44
45-55
56-66
67-77
78-88
89-99
≥100
Anti-IFN-ω auto-Ab levels
B
Neutralization of IFN-α2
(10 ng/mL)
1/10 plasma dilution
Anti-type I IFN auto-Ab positive
critical patients (%)
20
15
10
5
0
<40
40-49
50-59
60-69
70-79
≥80
Age (years)
C
Neutralization of IFN-α2
(10 ng/mL)
1/10 plasma dilution
Men
Women
D
Neutralization of IFN-α2 only
(10 ng/mL)
1/10 plasma dilution
E
Neutralization of IFN-α2 only
(10 ng/mL)
1/10 plasma dilution
Men
Women
F
Neutralization of IFN-ω
(10 ng/mL)
1/10 plasma dilution
G
Neutralization of IFN-ω
(10 ng/mL)
1/10 plasma dilution
Men
Women
H
Neutralization of IFN-ω only
(10 ng/mL)
1/10 plasma dilution
I
Neutralization of IFN-ω only
(10 ng/mL)
1/10 plasma dilution
Men
Women

Figure S3

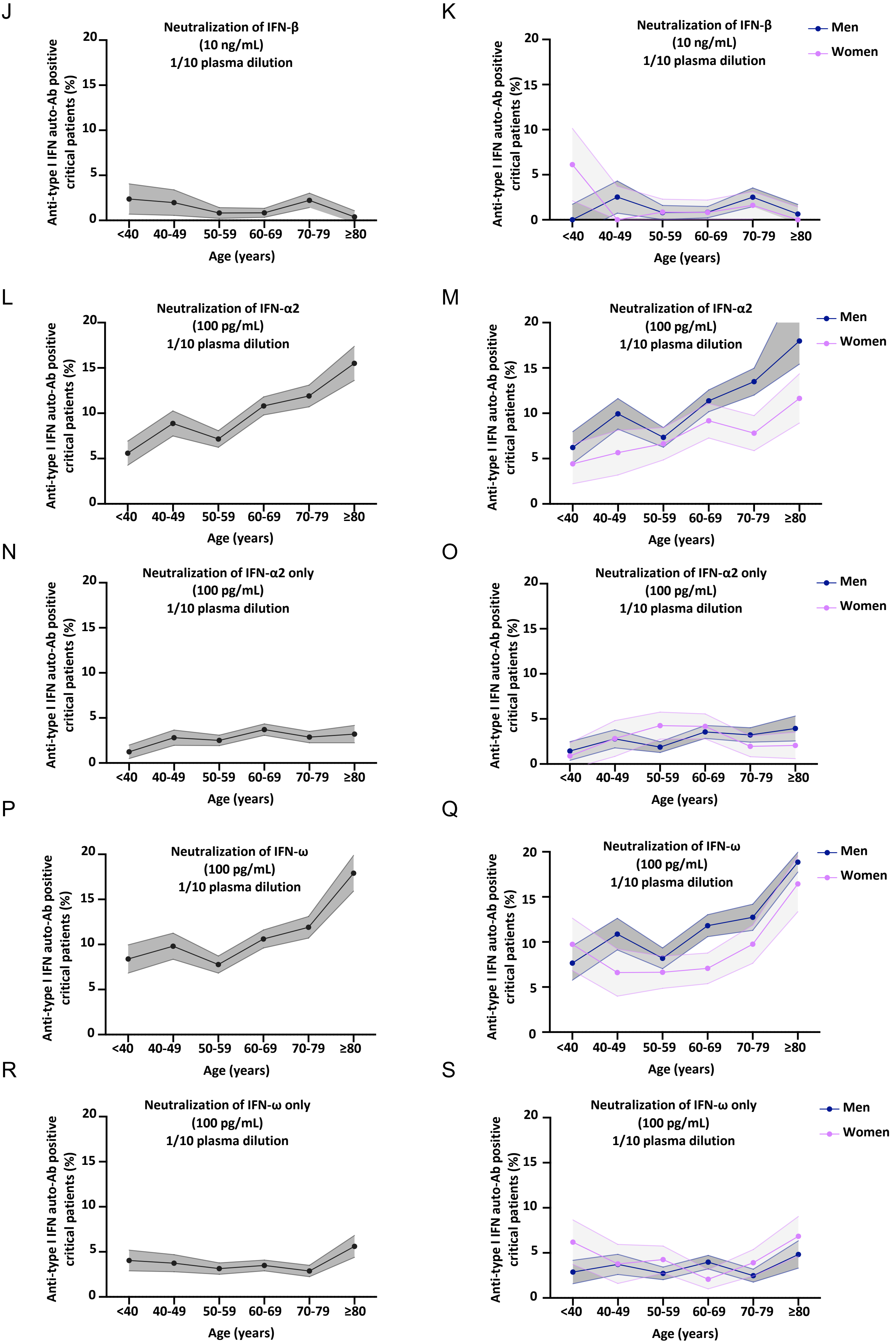
J
Neutralization of IFN-β
(10 ng/mL)
1/10 plasma dilution
Anti-type I IFN auto-Ab positive critical patients (%)
0
5
10
15
20
<40
40-49
50-59
60-69
70-79
≥80
Age (years)
K
Neutralization of IFN-β
(10 ng/mL)
1/10 plasma dilution
Men
Women
Anti-type I IFN auto-Ab positive critical patients (%)
Age (years)
L
Neutralization of IFN-α2
(100 pg/mL)
1/10 plasma dilution
Anti-type I IFN auto-Ab positive critical patients (%)
Age (years)
M
Neutralization of IFN-α2
(100 pg/mL)
1/10 plasma dilution
Men
Women
Anti-type I IFN auto-Ab positive critical patients (%)
Age (years)
N
Neutralization of IFN-α2 only
(100 pg/mL)
1/10 plasma dilution
Anti-type I IFN auto-Ab positive critical patients (%)
Age (years)
O
Neutralization of IFN-α2 only
(100 pg/mL)
1/10 plasma dilution
Men
Women
Anti-type I IFN auto-Ab positive critical patients (%)
Age (years)
P
Neutralization of IFN-ω
(100 pg/mL)
1/10 plasma dilution
Anti-type I IFN auto-Ab positive critical patients (%)
Age (years)
Q
Neutralization of IFN-ω
(100 pg/mL)
1/10 plasma dilution
Men
Women
Anti-type I IFN auto-Ab positive critical patients (%)
Age (years)
R
Neutralization of IFN-ω only
(100 pg/mL)
1/10 plasma dilution
Anti-type I IFN auto-Ab positive critical patients (%)
Age (years)
S
Neutralization of IFN-ω only
(100 pg/mL)
1/10 plasma dilution
Men
Women
Anti-type I IFN auto-Ab positive critical patients (%)
Age (years)

Figure S3

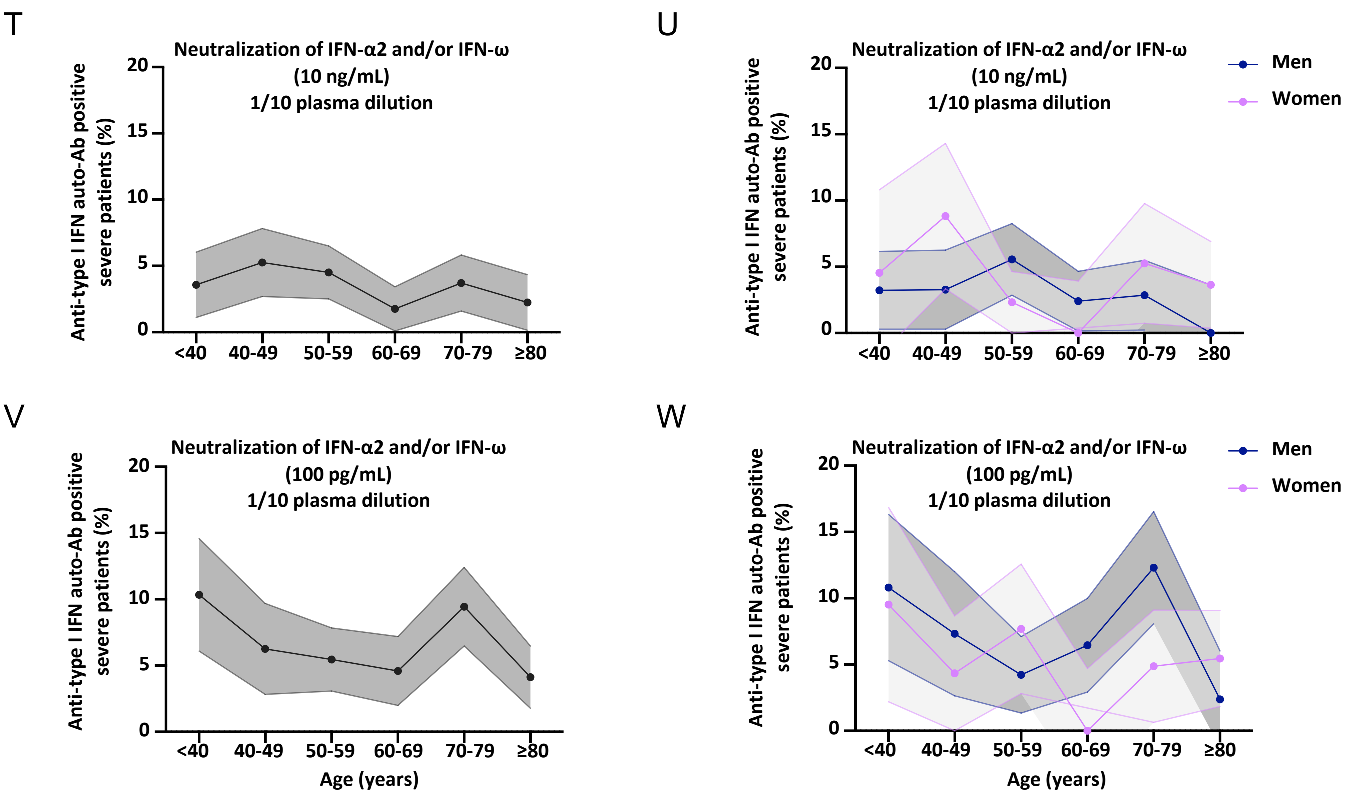

T
Neutralization of IFN-α2 and/or IFN-ω
(10 ng/mL)
1/10 plasma dilution
Anti-type I IFN auto-Ab positive severe patients (%)
0
5
10
15
20
<40
40-49
50-59
60-69
70-79
≥80
U
Neutralization of IFN-α2 and/or IFN-ω
(10 ng/mL)
1/10 plasma dilution
Men
Women
Anti-type I IFN auto-Ab positive severe patients (%)
V
Neutralization of IFN-α2 and/or IFN-ω
(100 pg/mL)
1/10 plasma dilution
Anti-type I IFN auto-Ab positive severe patients (%)
Age (years)
W
Neutralization of IFN-α2 and/or IFN-ω
(100 pg/mL)
1/10 plasma dilution
Men
Women
Anti-type I IFN auto-Ab positive severe patients (%)
Age (years)

Figure S4

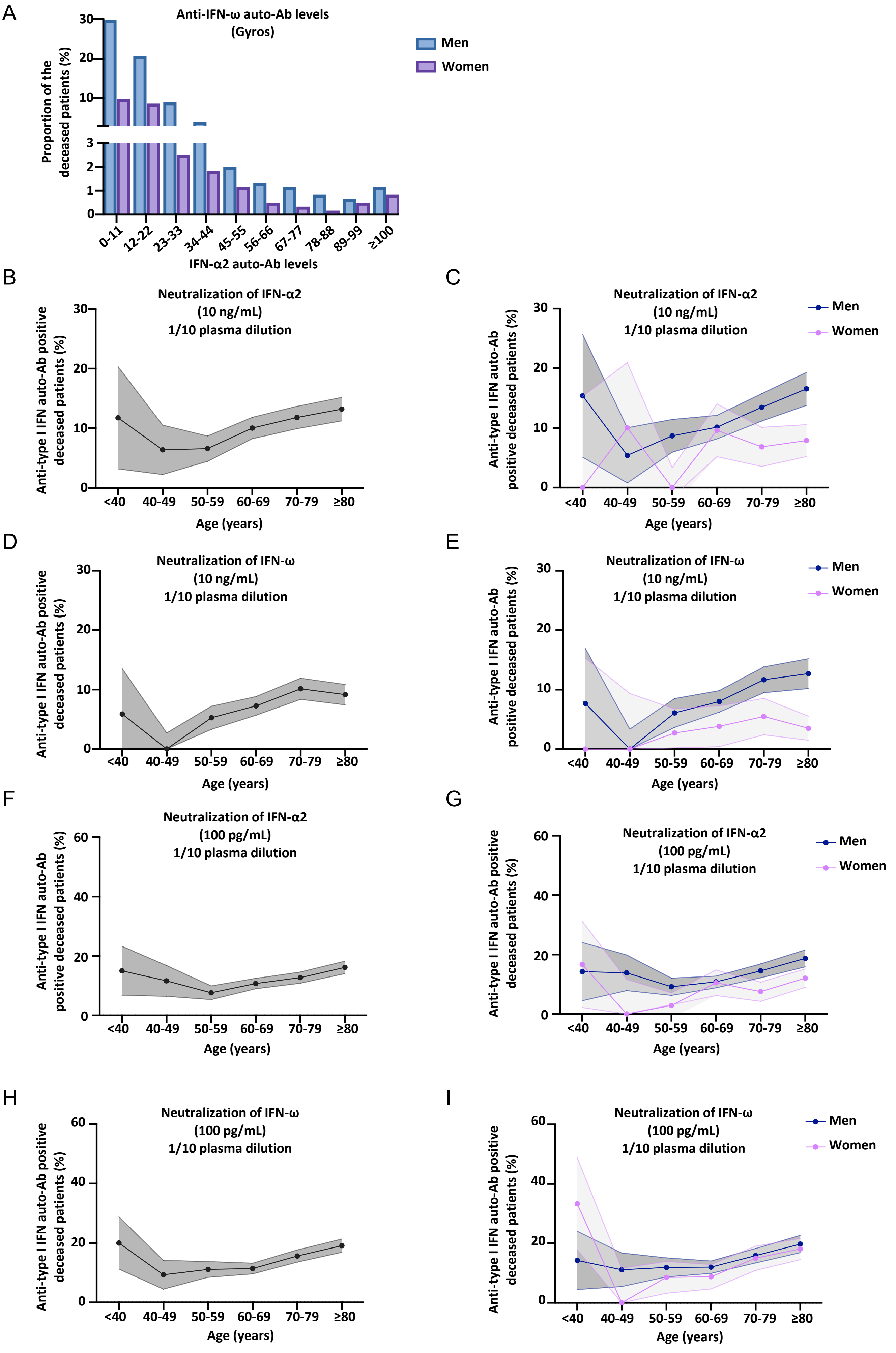
A
Anti-IFN-ω auto-Ab levels
(Gyros)
Men
Women
Proportion of the deceased patients (%)
IFN-α2 auto-Ab levels
0-11
12-22
23-33
34-44
45-55
56-66
67-77
78-88
89-99
≥100
B
Neutralization of IFN-α2
(10 ng/mL)
1/10 plasma dilution
Anti-type I IFN auto-Ab positive deceased patients (%)
Age (years)
C
Neutralization of IFN-α2
(10 ng/mL)
1/10 plasma dilution
Men
Women
Anti-type I IFN auto-Ab positive deceased patients (%)
Age (years)
D
Neutralization of IFN-ω
(10 ng/mL)
1/10 plasma dilution
Anti-type I IFN auto-Ab positive deceased patients (%)
Age (years)
E
Neutralization of IFN-ω
(10 ng/mL)
1/10 plasma dilution
Men
Women
Anti-type I IFN auto-Ab positive deceased patients (%)
Age (years)
F
Neutralization of IFN-α2
(100 pg/mL)
1/10 plasma dilution
Anti-type I IFN auto-Ab positive deceased patients (%)
Age (years)
G
Neutralization of IFN-α2
(100 pg/mL)
1/10 plasma dilution
Men
Women
Anti-type I IFN auto-Ab positive deceased patients (%)
Age (years)
H
Neutralization of IFN-ω
(100 pg/mL)
1/10 plasma dilution
Anti-type I IFN auto-Ab positive deceased patients (%)
Age (years)
I
Neutralization of IFN-ω
(100 pg/mL)
1/10 plasma dilution
Men
Women
Anti-type I IFN auto-Ab positive deceased patients (%)
Age (years)
<40
40-49
50-59
60-69
70-79
≥80

Figure S4

J

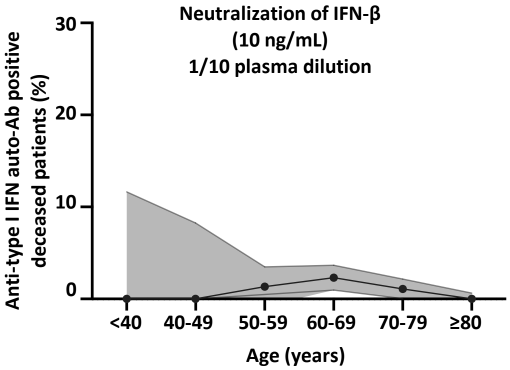

Neutralization of IFN-β
(10 ng/mL)
1/10 plasma dilution
Anti-type I IFN auto-Ab positive deceased patients (%)
30
20
10
0
<40
40-49
50-59
60-69
70-79
≥80
Age (years)

K

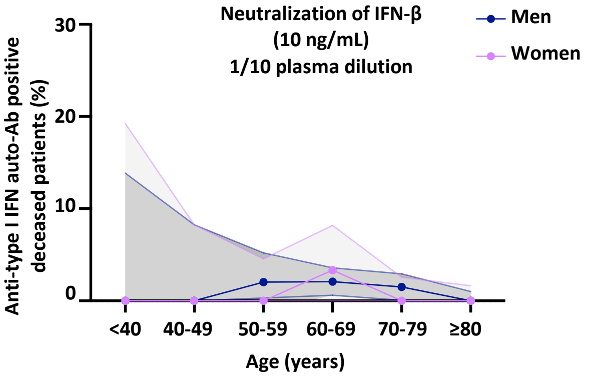

Neutralization of IFN-β
(10 ng/mL)
1/10 plasma dilution
Men
Women
Anti-type I IFN auto-Ab positive deceased patients (%)
30
20
10
0
<40
40-49
50-59
60-69
70-79
≥80
Age (years)

Figure S5

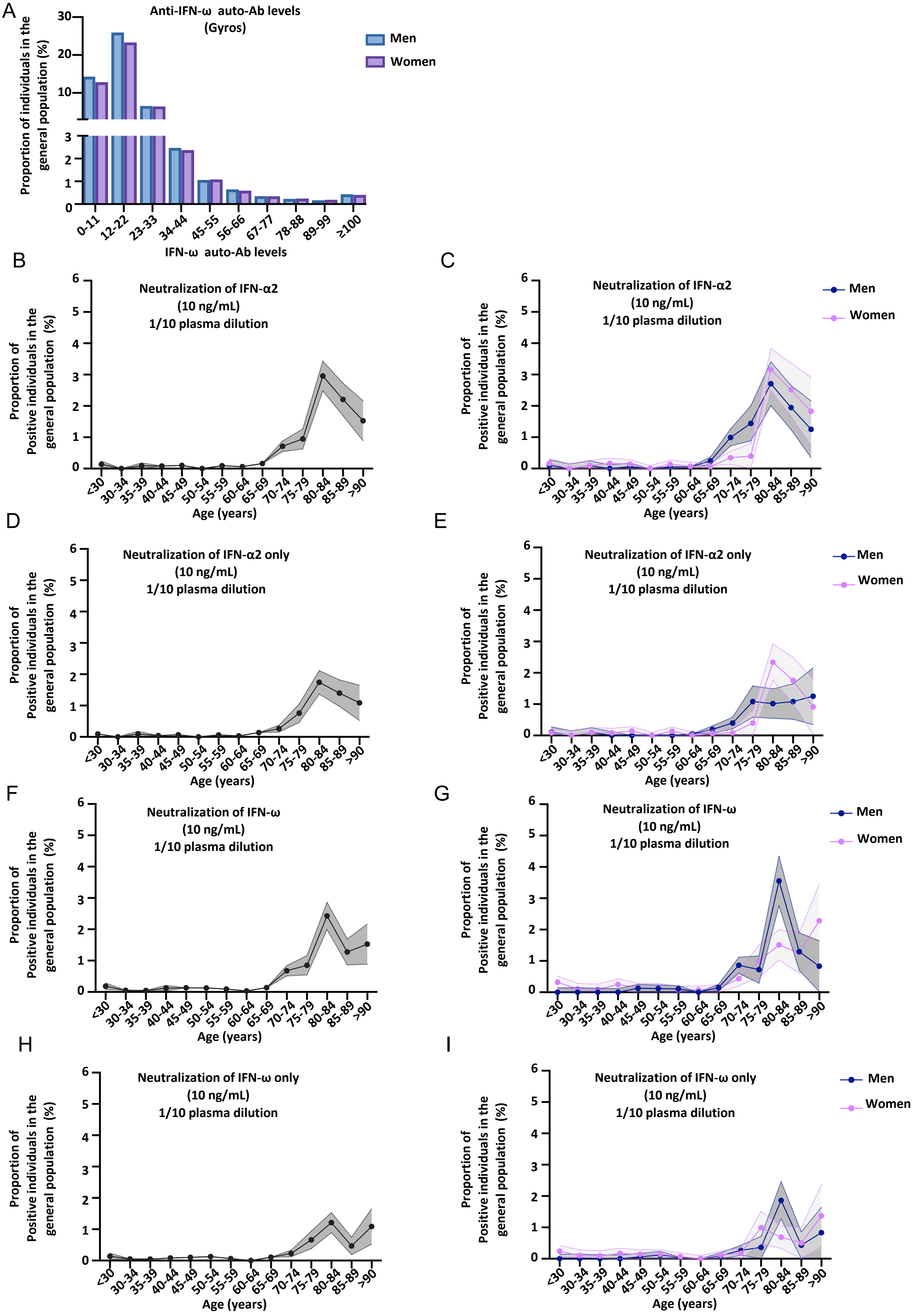
A
Anti-IFN-ω auto-Ab levels
(Gyros)
Men
Women
Proportion of individuals in the general population (%)
IFN-ω auto-Ab levels
0-11
12-22
23-33
34-44
45-55
56-66
67-77
78-88
89-99
≥100
B
Neutralization of IFN-α2
(10 ng/mL)
1/10 plasma dilution
Proportion of Positive individuals in the general population (%)
Age (years)
C
Neutralization of IFN-α2
(10 ng/mL)
1/10 plasma dilution
Men
Women
D
Neutralization of IFN-α2 only
(10 ng/mL)
1/10 plasma dilution
E
Neutralization of IFN-α2 only
(10 ng/mL)
1/10 plasma dilution
Men
Women
F
Neutralization of IFN-ω
(10 ng/mL)
1/10 plasma dilution
G
Neutralization of IFN-ω
(10 ng/mL)
1/10 plasma dilution
Men
Women
H
Neutralization of IFN-ω only
(10 ng/mL)
1/10 plasma dilution
I
Neutralization of IFN-ω only
(10 ng/mL)
1/10 plasma dilution
Men
Women
<30
30-34
35-39
40-44
45-49
50-54
55-59
60-64
65-69
70-74
75-79
80-84
85-89
>90

Figure S5

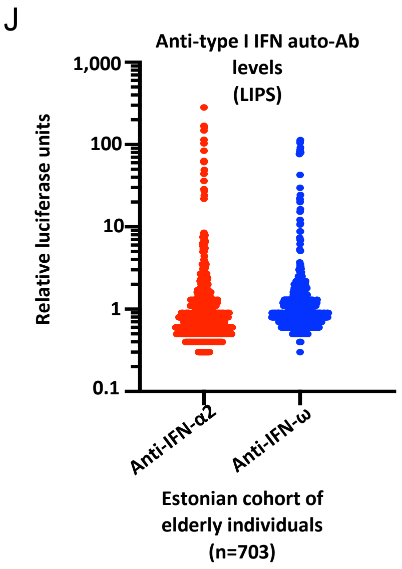
J
Anti-type I IFN auto-Ab levels (LIPS)
Relative luciferase units
1,000
100
10
1
0.1
Anti-IFN-α2
Anti-IFN-ω
Estonian cohort of elderly individuals (n=703)

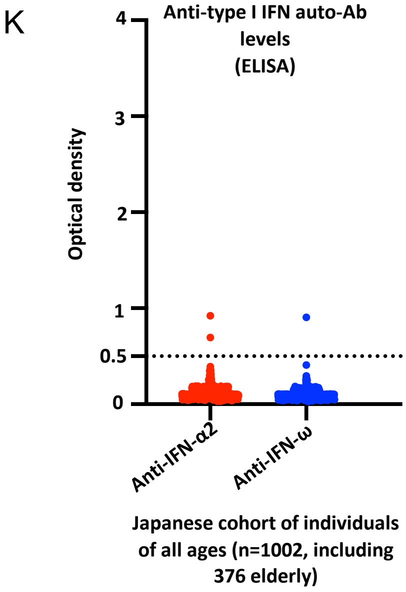
K
Anti-type I IFN auto-Ab levels (ELISA)
Optical density
4
3
2
1
0.5
0
Anti-IFN-α2
Anti-IFN-ω
Japanese cohort of individuals of all ages (n=1002, including 376 elderly)

Figure S6

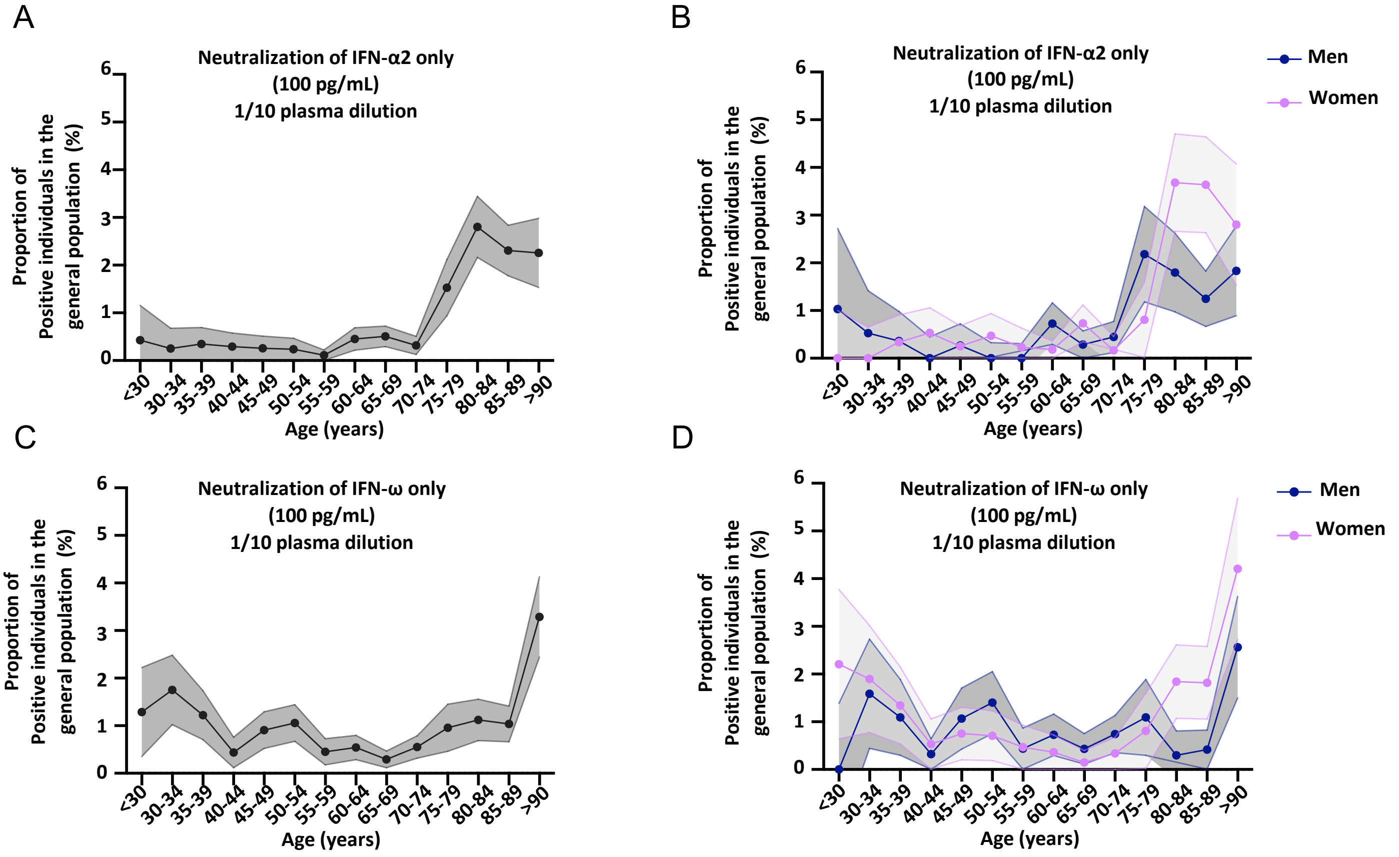

A
Neutralization of IFN-α2 only
(100 pg/mL)
1/10 plasma dilution
Proportion of Positive individuals in the general population (%)
Age (years)
<30
30-34
35-39
40-44
45-49
50-54
55-59
60-64
65-69
70-74
75-79
80-84
85-89
>90
B
Neutralization of IFN-α2 only
(100 pg/mL)
1/10 plasma dilution
Men
Women
C
Neutralization of IFN-ω only
(100 pg/mL)
1/10 plasma dilution
D
Neutralization of IFN-ω only
(100 pg/mL)
1/10 plasma dilution
Men
Women
